# Supplementary material for: Cross-attention enables deep learning on limited omics-imaging-clinical data of 130 lung cancer patients
Source: Cell Rep Methods. 2024 Jul 8;4(7):100817. doi: 10.1016/j.crmeth.2024.100817 (PMC11294841; doi:10.1016/j.crmeth.2024.100817)
Supplement: Document S1. Figures S1–S4, Tables S1 and S2 [file mmc1.pdf]

Cell Reports Methods, Volume *4*

Supplemental information

Cross-attention enables deep learning on limited omics-imaging-clinical data of 130 lung cancer patients

Suraj Verma, Giuseppe Magazzù, Noushin Eftekhari, Thai Lou, Alex Gilhespy, Annalisa Occhipinti, and Claudio Angione

Cross-attention enables deep learning on limited omics-imaging-clinical data of 130 lung cancer patients

Suraj Verma[1], Giuseppe Magazzù[2], Noushin Eftekhari[3], Thai Lou[4], Alex Gilhespy[5], Annalisa Occhipinti[1,6,7], and Claudio Angione *[1,6,7]

[1]School of Computing, Engineering & Digital Technologies, Teesside University, Tees Valley TS1 3BA, UK
[2]York St John University, York YO31 7EX, UK
[3]The Alan Turing Institute, London NW1 2DB, UK
[4]Gateshead Health NHS Foundation Trust, Gateshead NE9 6SX, UK
[5]South Tyneside and Sunderland NHS Foundation Trust, Sunderland SR4 7TP, UK
[6]Centre for Digital Innovation, Teesside University, Campus Heart, Tees Valley TS1 3BX, UK
[7]National Horizons Centre, Darlington DL1 1HG, UK

June 16, 2024

*Corresponding author

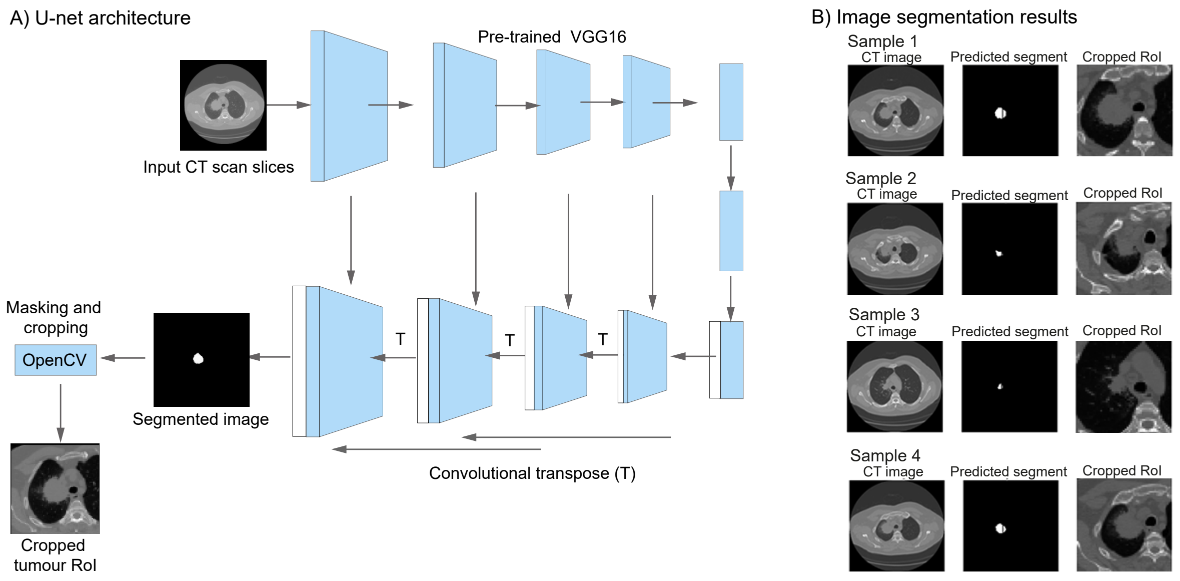

Figure S1: **Preprocessing of CT-scan images to extract regions of interest, related to STAR Method.** (A) A U-net-based architecture was designed using a pre-trained VGG16 model in the contraction path. Transpose convolutional layers were implemented in the expansion path. Using the OpenCV library, the predicted segment was masked with a tumour image and the tumour region was cropped to extract the Region of Interest (RoI). (B) Examples of tumour regions segmented using the U-Net model and RoI extracted using the OpenCV library after the segmentation pipeline.

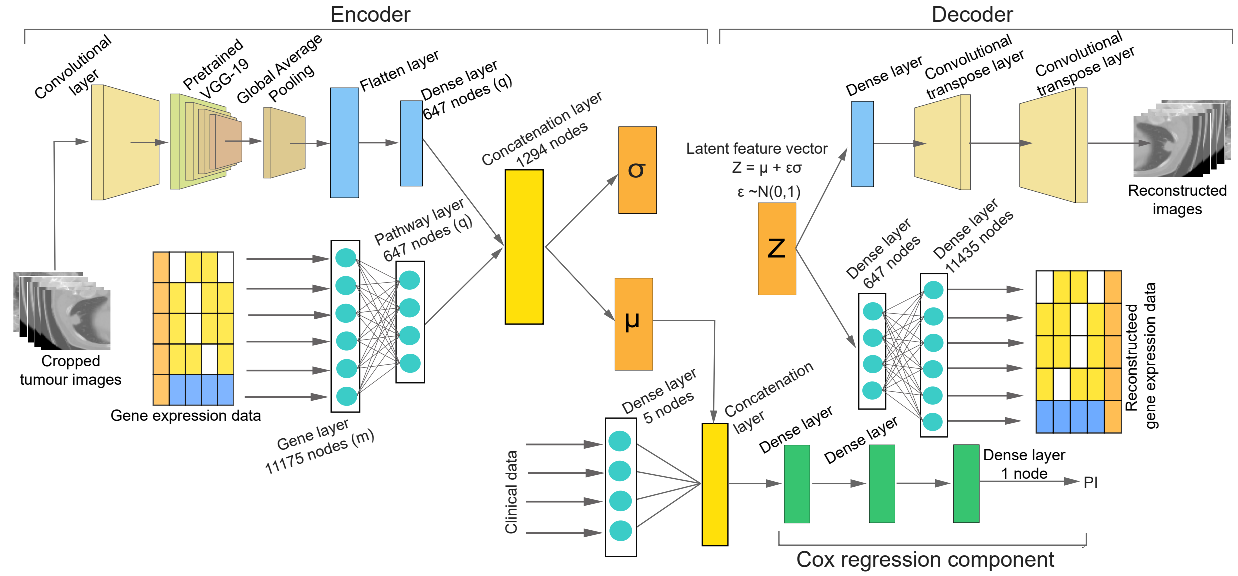

Figure S2: **Baseline-VAE-Cox: Variational Autoencoder-based Cox model, related to Figure 3.** The β-Variational Autoencoder architecture consists of encoder and decoder phases made from a convolutional layer, pre-trained VGG-19 layers, and dense layers for image and gene expression data. The latent vector μ is linked to the Cox regression component, which concatenates the latent vector and clinical features to estimate the Prognostic Index (PI).

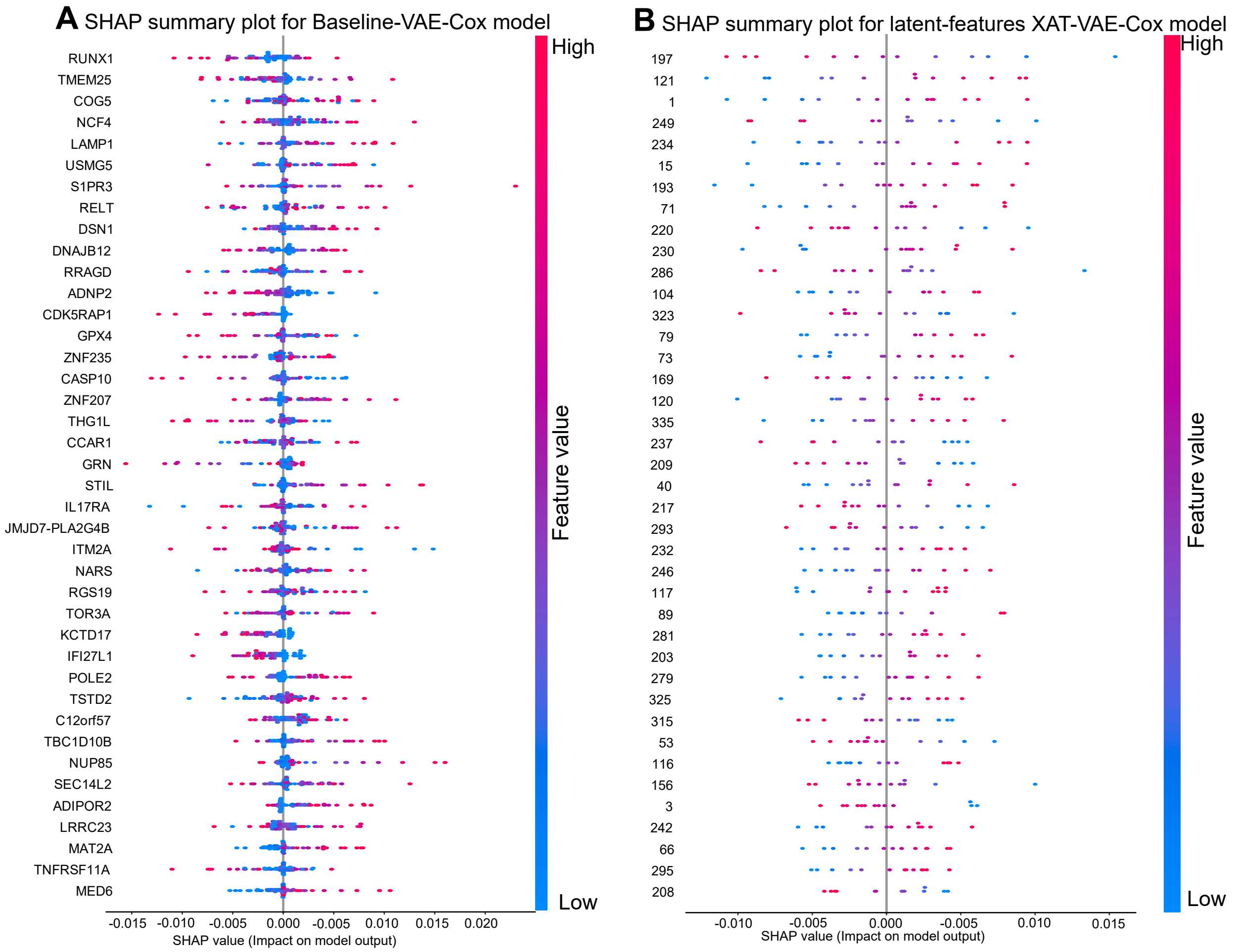

Figure S3: **Feature importance by SHAP summary plot, related to Figure 5** (A) Summary plot for top 40 important genes for Baseline-VAE-Cox model. (B) Summary plot for top 40 latent features μ generated by the integration of imaging and gene expression in the XAT-VAE-Cox model. As the XAT-VAE-Cox model generates a single latent representation from both image and gene modalities, the interpretation of these latent features did not provide any insights into the contribution of each modality towards the estimation of the prognostic index.

Figure S4: **Grad-CAM-highlighted CT scan images validated by the radiologists, related to Figure 5.** The following four figures report the labelling performed by two radiologists, who validated the tumour areas highlighted by the Grad-CAM heatmaps. Each image was evaluated based on three questions. The first question was whether the heatmap matched the tumour region, with a yes or no answer. The second and third questions asked for a rating from 1 to 4 on the quality and clarity of the heatmaps (see Main Manuscript).

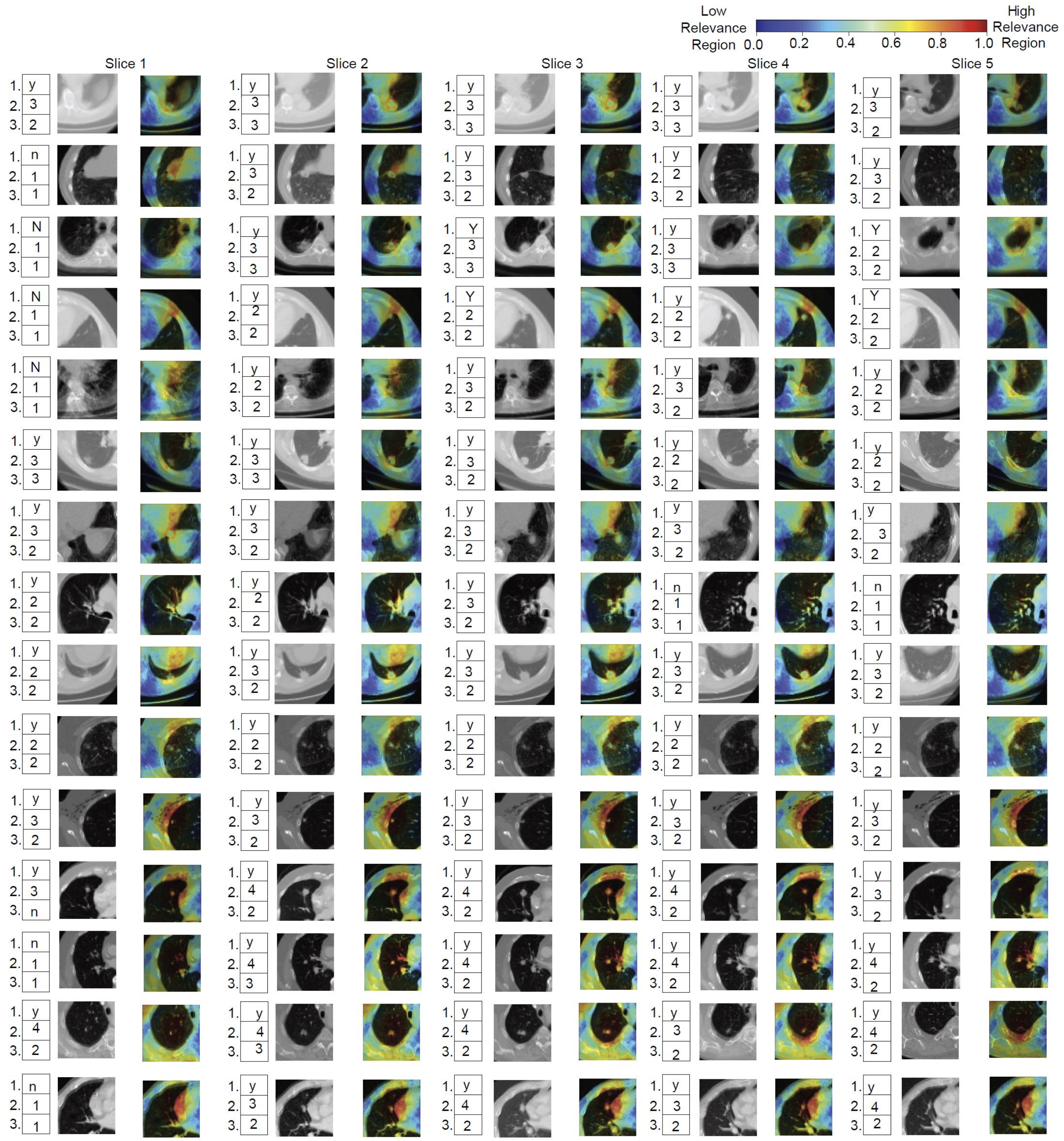

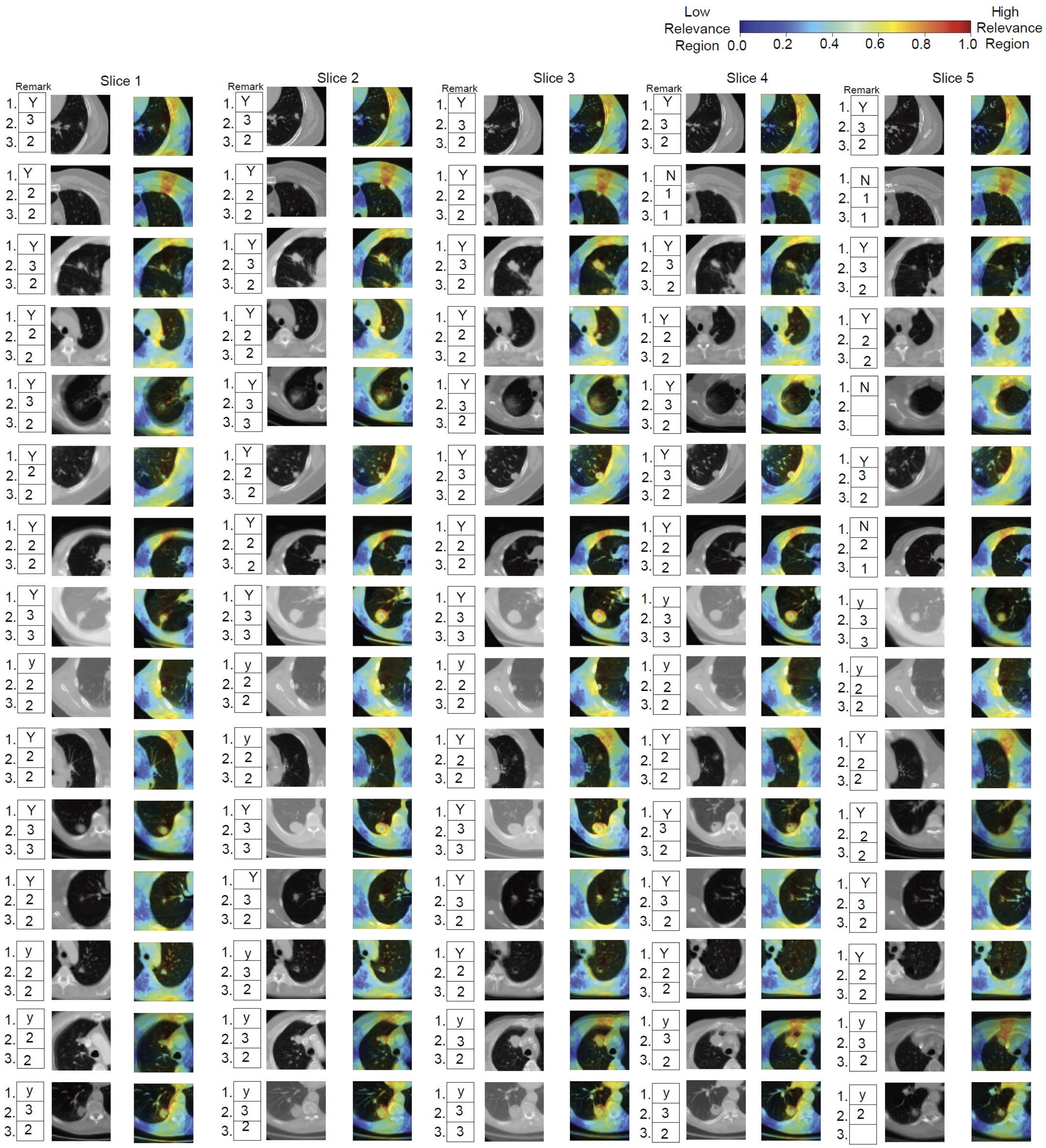
Low Relevance Region
0.0
0.2
0.4
0.6
0.8
1.0
High Relevance Region
Remark
Slice 1
Slice 2
Slice 3
Slice 4
Slice 5

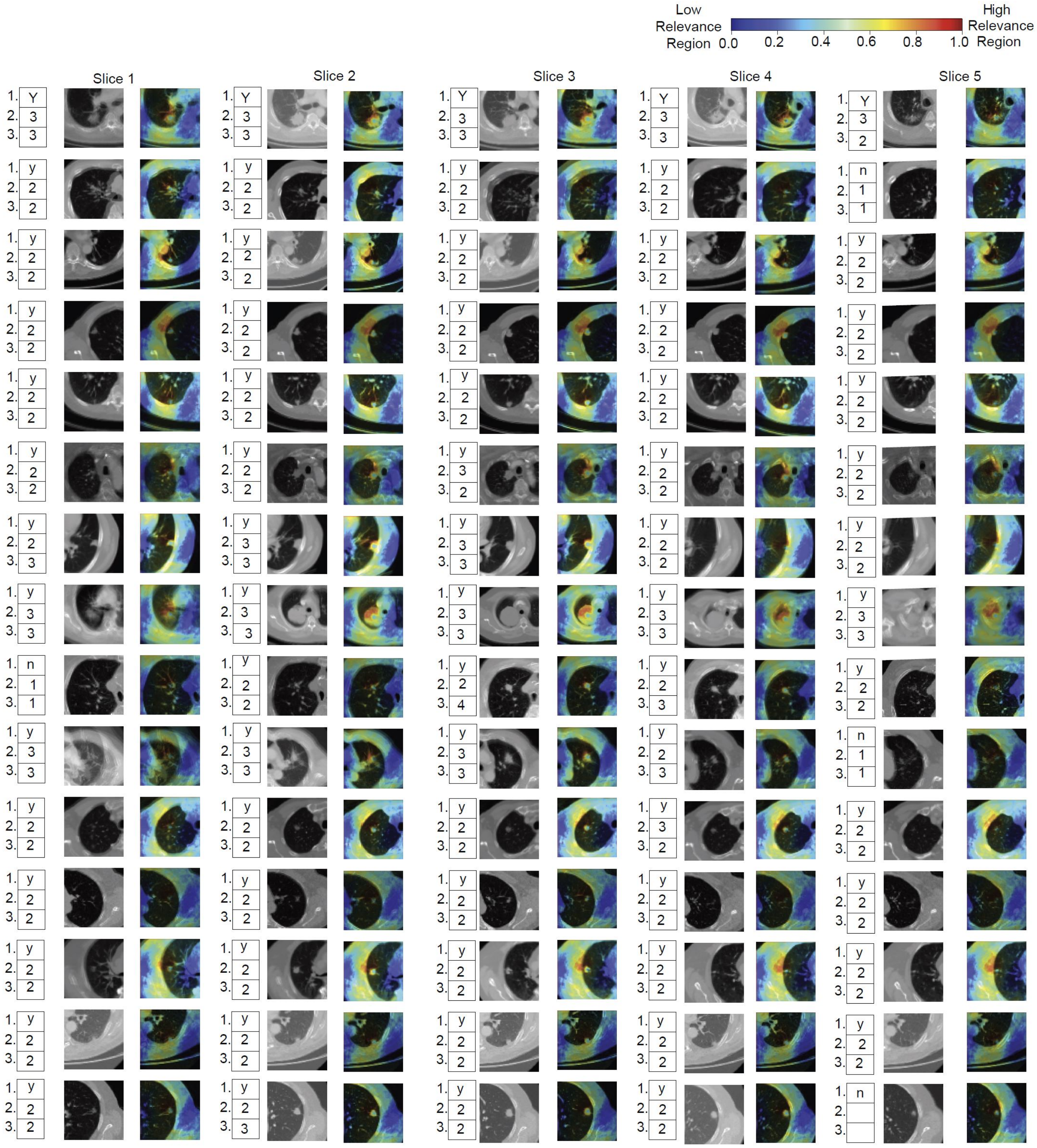
Low Relevance Region
High Relevance Region
0.0 0.2 0.4 0.6 0.8 1.0
Slice 1
Slice 2
Slice 3
Slice 4
Slice 5
1. Y 2. 3 3. 3
1. Y 2. 3 3. 3
1. Y 2. 3 3. 3
1. Y 2. 3 3. 3
1. Y 2. 3 3. 2
1. y 2. 2 3. 2
1. y 2. 2 3. 2
1. y 2. 2 3. 2
1. y 2. 2 3. 2
1. n 2. 1 3. 1
1. y 2. 2 3. 2
1. y 2. 2 3. 2
1. y 2. 2 3. 2
1. y 2. 2 3. 2
1. y 2. 2 3. 2
1. y 2. 2 3. 2
1. y 2. 2 3. 2
1. y 2. 2 3. 2
1. y 2. 2 3. 2
1. y 2. 2 3. 2
1. y 2. 2 3. 2
1. y 2. 2 3. 2
1. y 2. 2 3. 2
1. y 2. 2 3. 2
1. y 2. 2 3. 2
1. y 2. 2 3. 2
1. y 2. 2 3. 2
1. y 2. 3 3. 2
1. y 2. 2 3. 2
1. y 2. 2 3. 2
1. y 2. 2 3. 3
1. y 2. 3 3. 3
1. y 2. 3 3. 3
1. y 2. 2 3. 2
1. y 2. 2 3. 2
1. y 2. 3 3. 3
1. y 2. 3 3. 3
1. y 2. 3 3. 3
1. y 2. 3 3. 3
1. y 2. 3 3. 3
1. n 2. 1 3. 1
1. y 2. 2 3. 2
1. y 2. 2 3. 4
1. y 2. 2 3. 3
1. y 2. 2 3. 2
1. y 2. 3 3. 3
1. y 2. 3 3. 3
1. y 2. 3 3. 3
1. y 2. 2 3. 3
1. n 2. 1 3. 1
1. y 2. 2 3. 2
1. y 2. 2 3. 2
1. y 2. 2 3. 2
1. y 2. 3 3. 2
1. y 2. 2 3. 2
1. y 2. 2 3. 2
1. y 2. 2 3. 2
1. y 2. 2 3. 2
1. y 2. 2 3. 2
1. y 2. 2 3. 2
1. y 2. 2 3. 2
1. y 2. 2 3. 2
1. y 2. 2 3. 2
1. y 2. 2 3. 2
1. y 2. 2 3. 2
1. y 2. 2 3. 2
1. y 2. 2 3. 2
1. y 2. 2 3. 2
1. y 2. 2 3. 2
1. y 2. 2 3. 2
1. y 2. 2 3. 2
1. y 2. 2 3. 3
1. y 2. 2 3. 2
1. y 2. 2 3. 2
1. n 2. 3.

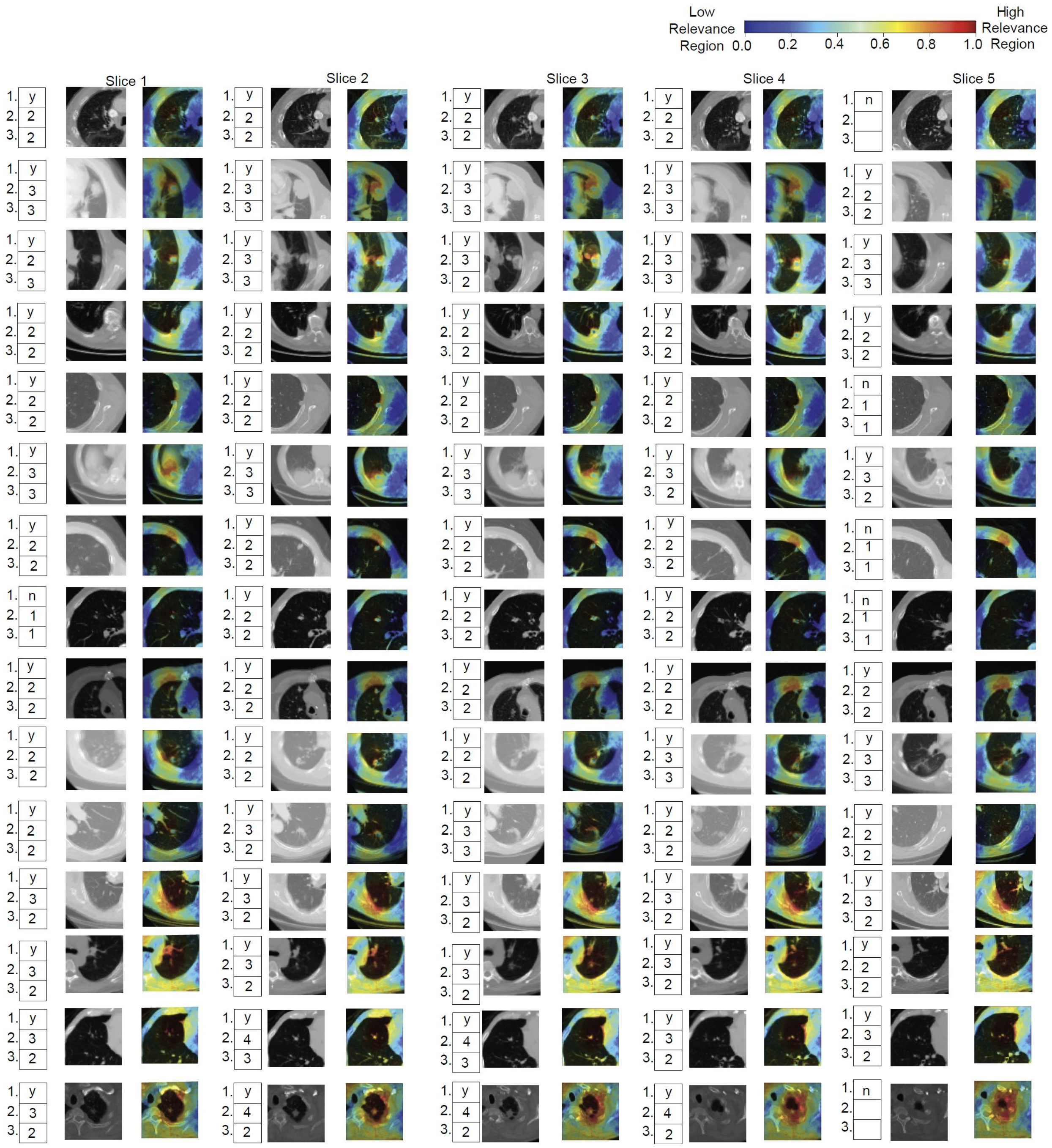
Low Relevance Region
High Relevance Region
0.0
0.2
0.4
0.6
0.8
1.0
Slice 1
Slice 2
Slice 3
Slice 4
Slice 5

Supplementary Tables

Models	Dataset	C-index IPCW (mean ± s.d)	Cumulative dynamic AUC (mean ± s.d)
H-VAE Cox	NSCLC-Radiogenomics	0.74 ± 0.09	0.73 ± 0.09
H-VAE Cox	TCGA-LUAD	0.71 ± 0.08	0.69 ± 0.09
H-VAE Cox	TCGA-LUSC	0.67 ± 0.10	0.68 ± 0.14
XAT-VAE-Cox	NSCLC-Radiogenomics	**0.77 ± 0.06**	**0.76 ± 0.06**
XAT-VAE-Cox	TCGA-LUAD	0.68 ± 0.03	0.69 ± 0.04
XAT-VAE-Cox	TCGA-LUSC	0.68 ± 0.05	0.71 ± 0.05
DeepSurv	NSCLC-Radiogenomics	0.60 ± 0.11	0.59 ± 0.13
DeepSurv	TCGA-LUAD	0.52 ± 0.31	0.49 ± 0.21
DeepSurv	TCGA-LUSC	0.53 ± 0.12	0.52 ± 0.02
DCM	NSCLC-Radiogenomics	0.64 ± 0.12	0.71 ± 0.14
DCM	TCGA-LUAD	0.57 ± 0.14	0.55 ± 0.11
DCM	TCGA-LUSC	0.52 ± 0.16	0.61 ± 0.13

Table S1: **Performance results of the proposed models when evaluated on NSCLC-Radiogenomics, TCGA-LUAD and TCGA-LUSC datasets in terms of C-index IPCW and Cumulative dynamic AUC (mean and standard deviation), related to Figure 4.** For each model, the nested cross-validation was repeated five times and the overall performance was presented in terms of mean and standard deviation. The values in bold show the best performance for each metric. Overall, the optimal performance was obtained by the XAT-VAE-Cox model, with a C-index IPCW of 77% and cumulative dynamic AUC of 76%.

Dataset	C-index (mean ± s.d)	C-index IPCW (mean ± s.d)	Cumulative dynamic AUC (mean ± s.d)
NSCLC-Radiogenomics	0.59 ± 0.08	0.63 ± 0.07	0.61 ± 0.11
TCGA-LUAD	0.58 ± 0.02	0.60 ± 0.02	0.60 ± 0.01
TCGA-LUSC	0.52 ± 0.05	0.58 ± 0.04	0.61 ± 0.05

Table S2: **Performance evaluation of the baseline-VAE-Cox model in terms of C-index, C-index IPCW and Cumulative dynamic AUC, related to Figure 4**
