# Supplementary material for: Cross-attention enables deep learning on limited omics-imaging-clinical data of 130 lung cancer patients
Source: Cell Rep Methods. 2024 Jul 8;4(7):100817. doi: 10.1016/j.crmeth.2024.100817 (PMC11294841; doi:10.1016/j.crmeth.2024.100817)
Supplement: Document S2. Article plus supplemental information [file mmc3.pdf]

Cell Reports Methods

Article

Cross-attention enables deep learning on limited omics-imaging-clinical data of 130 lung cancer patients

Graphical abstract

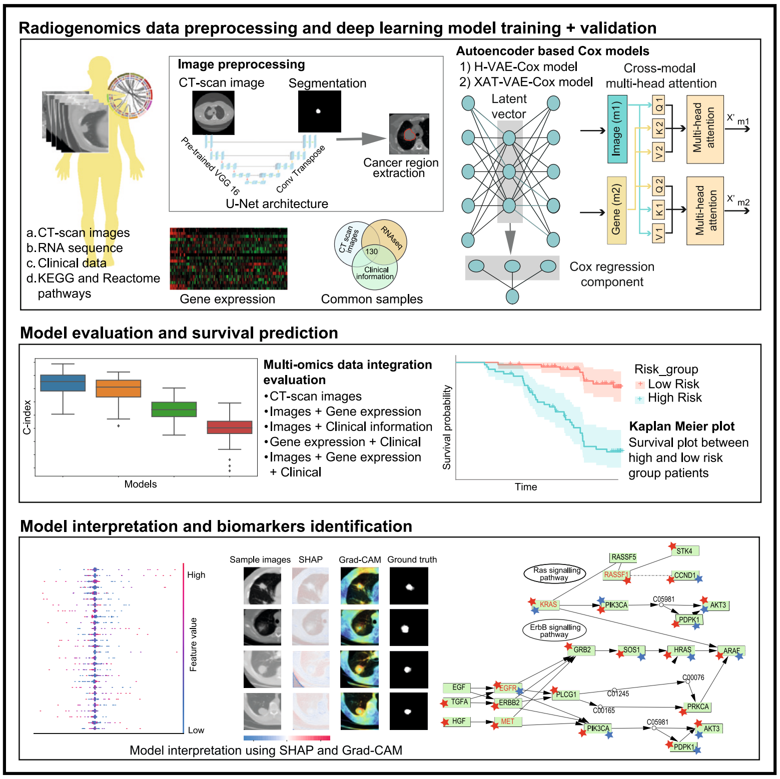

Highlights

- We propose interpretable deep-learning methods for patient survival prediction
- We integrate CT-scan images, gene expression, and clinical data from 130 patients
- Cross-attention improves performance when working with small datasets
- Models identify key gene biomarkers and highlight regions of interest in CT scans

Authors

Suraj Verma, Giuseppe Magazzù, Noushin Eftekhari, Thai Lou, Alex Gilhespy, Annalisa Occhipinti, Claudio Angione

Correspondence

c.angione@tees.ac.uk

In brief

Learning from small biomedical datasets is an open challenge for deep-learning models. Verma et al. propose interpretable and robust architectures for survival prediction of NSCLC patients, integrating CT-scan images, gene expression, and clinical data. A cross-attention mechanism significantly improves performance, correctly identifying genes and CT-scan regions as biomarkers.

Verma et al., 2024, Cell Reports Methods *4*, 100817
July 15, 2024 © 2024 The Author(s). Published by Elsevier Inc.
https://doi.org/10.1016/j.crmeth.2024.100817

Cell Reports Methods

CellPress
OPEN ACCESS

Article

Cross-attention enables deep learning on limited omics-imaging-clinical data of 130 lung cancer patients

Suraj Verma,[1] Giuseppe Magazzù,[2] Noushin Eftekhari,[3] Thai Lou,[4] Alex Gilhespy,[5] Annalisa Occhipinti,[1,6,7] and Claudio Angione[1,6,7,8,*]
[1]School of Computing, Engineering and Digital Technologies, Teesside University, Middlesbrough, UK
[2]York St John University, York, UK
[3]The Alan Turing Institute, London, UK
[4]Gateshead Health NHS Foundation Trust, Gateshead, UK
[5]South Tyneside and Sunderland NHS Foundation Trust, Sunderland, UK
[6]Centre for Digital Innovation, Teesside University, Middlesbrough, UK
[7]National Horizons Centre, Teesside University, Darlington, UK
[8]Lead contact
*Correspondence: c.angione@tees.ac.uk
https://doi.org/10.1016/j.crmeth.2024.100817

MOTIVATION Multimodal deep-learning models can be used to obtain personalized survival predictions. However, the small size of most matched omics-imaging-clinical studies currently poses significant challenges to the development and application of such tools. Furthermore, the lack of interpretability makes it difficult to understand the biological rationale behind the predictions, leading to a lack of trust and reluctance to adopt them in clinical settings. Specifically, the inability to explain how specific features contribute to the predictions limits the potential for new insights and identification of prognostic biomarkers. We propose two biologically interpretable and robust deep-learning architectures for survival prediction of 130 non-small cell lung cancer (NSCLC) patients, integrating patient-specific clinical, transcriptomic, and imaging data. We incorporate KEGG and Reactome pathway information, adding biological knowledge within the learning process. Introducing a cross-attention mechanism in a sparse autoencoder allows extracting prognostic gene biomarkers and molecular pathways that are biologically interpretable even in the presence of small samples and highlights tumor regions successfully validated by two radiologists.

SUMMARY

Deep-learning tools that extract prognostic factors derived from multi-omics data have recently contributed to individualized predictions of survival outcomes. However, the limited size of integrated omics-imaging-clinical datasets poses challenges. Here, we propose two biologically interpretable and robust deep-learning architectures for survival prediction of non-small cell lung cancer (NSCLC) patients, learning simultaneously from computed tomography (CT) scan images, gene expression data, and clinical information. The proposed models integrate patient-specific clinical, transcriptomic, and imaging data and incorporate Kyoto Encyclopedia of Genes and Genomes (KEGG) and Reactome pathway information, adding biological knowledge within the learning process to extract prognostic gene biomarkers and molecular pathways. While both models accurately stratify patients in high- and low-risk groups when trained on a dataset of only 130 patients, introducing a cross-attention mechanism in a sparse autoencoder significantly improves the performance, highlighting tumor regions and NSCLC-related genes as potential biomarkers and thus offering a significant methodological advancement when learning from small imaging-omics-clinical samples.

INTRODUCTION

Lung cancer is one of the most prevalent types of cancer worldwide, having a high incidence rate and low 5-year survival rate.[1,2] More than 85% of lung cancer cases are non-small cell lung cancer (NSCLC), and around one-third of NSCLC cases are identified at a locally advanced stage.[3,4] Even when NSCLC is detected in stages I and II, about a quarter of patients experience

This is an open access article under the CC BY license (http://creativecommons.org/licenses/by/4.0/).

postoperative recurrence, with the majority dying from the recurrence of the disease. The overall 5-year survival of NSCLC patients for stages I, II, and III is 55%, 35%, and 15%, respectively.[5] Furthermore, depending on how far the tumor has spread, the 5-year relative survival rates for those with regional involvement (cancer disseminated outside the lung or lymph nodes) and localized (cancer limited to one lung) NSCLC are 34.5% and 61.4%, respectively.[6] Therefore, NSCLC is one of the leading causes of cancer deaths, which can only be reduced by precise diagnosis, prognosis, and personalized treatments.

To date, studies on personalized medicine have mostly focused on molecular characterization using omics technologies (e.g., transcriptomics, genomics, metabolomics, and proteomics).[7] However, these approaches need tissue samples obtained by invasive biopsy or surgery,[8] and NSCLC patients often have an insufficient amount of tissue that can be sampled at diagnosis.[9] As cancer tumors are heterogeneous lesions, samples taken from a small area of the lesion may not adequately reflect the anatomic, functional, or physiologic characteristics of the entire lesion.[10] On the other hand, imaging techniques hold great potential for tumor characterization as they provide a more general view of the tumor than biopsy samples alone.[11–14] The integration of radiological images and multi-omics data is an emerging field, and a wide range of studies have been carried out for various applications, including radiogenomics data analysis for disease diagnosis, image and gene expression correlation analysis, and survival prediction.[15–26] Recently, several approaches have been proposed to predict patient survival by combining the power of traditional survival analysis methods with various machine-learning techniques, with the aim of predicting event occurrence at a given point in time. Such techniques are best suited for high-dimensional data because of their ability to perform survival analysis using both statistical and machine-learning methods.[27–29]

In learning architectures for survival analysis, despite the recent surge in multimodal data generation, achieving a reliable, precise, and interpretable prognosis remains an open challenge, with existing methods achieving satisfactory but not high accuracy. For instance, Ellen et al.,[24] proposed an autoencoder-based multimodal model for survival prediction of NSCLC (i.e., lung adenocarcinoma [LUAD] and lung squamous cell carcinoma [LUSC]), using microRNA (miRNA), messenger RNA (mRNA), DNA methylation, long non-coding RNA (lncRNA), and clinical data from 732 common samples. For the LUAD dataset (408 samples), the model achieved a C-index of 0.67 ± 0.04 for early integration and late integration of different combinations of data, while, for the LUSC dataset (324 samples), the model achieved a C-index of 0.63 ± 0.02 for early integration and 0.59 ± 0.03 for late integration of different combinations of data. In another paper, Jiang et al.[22] proposed an attention-based model to predict survival for four cancer types: bladder cancer (BLCA), breast cancer (BRCA), colon adenocarcinoma (COAD), and lower-grade glioma (LGG) each with 386, 1,050, 449, and 490 samples, respectively, using whole-slide images. The models achieved a C-index of 0.604, 0.607, 0.636, and 0.714, respectively.

The challenge of achieving high accuracy is due to various reasons, including the small size of imaging-omics datasets, the heterogeneity of multi-dimensional images, the high dimensionality and low sample size of omics data, and the complex non-linearity in biological components. Several dimensionality reduction algorithms, such as mutual information-based feature selection (MIFS), minimum redundancy maximum relevance (mRMR), and normalized mutual information feature selection (NMIFS), are widely used to reduce the dimension of omics data.[30,31] These dimensionality reduction techniques, however, are data driven and may therefore lose biologically significant features. New methods of interpretability can identify cancer-related features and biomarkers that play a significant role in estimating cancer survival and patient-specific survival prediction. Therefore, there is a need to design robust and biologically interpretable deep neural networks for survival analysis using high-dimension and low-sample-size integrated features from radiomic, genomics (collectively called radiogenomics), and clinical information for NSCLC.

Recent research on survival prediction using multimodal deep-learning architectures has demonstrated that integrating multi-omics data using multimodal models enhances survival prediction when compared to single-omic data.[32–39] Several autoencoder-based models have been developed for survival analysis using single- or multi-omics data.[40–46] However, these models do not consider images with other omics data for survival prediction. Furthermore, as these do not incorporate biological pathways-related information within the learning process, they tend to lose biological information while generating latent features. These shortcomings directly affect the reliability and interpretability of such autoencoder-based models.

Here, we propose two sparse variational autoencoder-based methods, namely a hierarchical variational autoencoder-based Cox model (H-VAE-Cox) and a cross-attention-based sparse variational autoencoder Cox (XAT-VAE-Cox) model, for the intermediate integration of multi-dimensional computed tomography (CT) scan images, gene expression, and clinical profiles, incorporating biological knowledge into the models. In particular, a sparse matrix of Kyoto Encyclopedia of Genes and Genomes (KEGG) and Reactome pathway information was used to create the sparsity between the gene and pathway layers of the models, adding important biological knowledge. We show that both the proposed models incorporate patient-specific data and KEGG-Reactome pathway information as additional biological knowledge within the learning process and can extract prognostic gene biomarkers and molecular pathways. Both models accurately stratify patients into risk groups when trained on a small dataset of only 130 patients, therefore representing a new method to learn on typically small datasets with matched imaging, omics, and clinical information for the same patients.

While both approaches accurately stratify patients into risk groups, the best model to be adopted depends on the modeling priorities. Specifically, H-VAE-Cox, being a modular model, requires fewer computational resources. Conversely, XAT-VAE-Cox, with an attention mechanism, can learn from cross-modality information (imaging and gene modalities) and incorporate biological information. XAT-VAE-Cox also considered a larger number of NSCLC-related genes as important genes for survival prediction, providing better overall biological interpretation.

CellPress
OPEN ACCESS

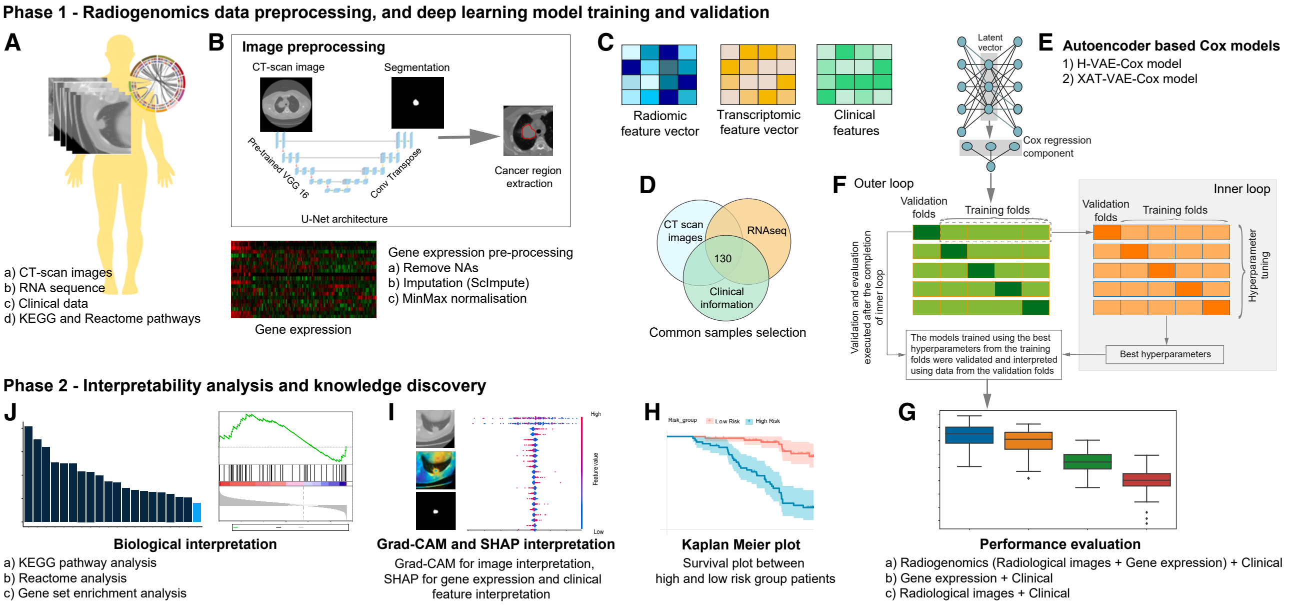

Figure 1. Workflow of our study

(A) Radiogenomics data (CT-scan images and gene expression) along with clinical data were collected from TCIA and GEO.

(B) The collected data were then preprocessed as follows. First, the ROIs, i.e., tumor regions, were segmented using the U-Net model, the null values from gene expression data were removed, and the resulting data were finally normalized.

(C and D) Feature selection was then performed on images, gene expression, and clinical data, and 130 common samples from all the datasets were selected to be fed into the deep-learning models.

(E) The deep-learning models estimate the PI using images, gene expression, and clinical data.

(F) To ensure robustness, the models were trained and validated using a nested cross-validation approach, where the inner loops were used to tune the hyperparameters and the outer loops were used to validate and evaluate the models. The SHAP and biological interpretations in the following steps were performed on the outer-loop validation folds.

(G and H) (G) The results from the models were evaluated using C-index, and KM curves (H) were plotted. A log rank test was performed to measure the classification accuracy of high- and low-risk group patients.

(I and J) (I) The models were interpreted using Grad-CAM and SHAP values and, finally, the significant genes identified in the analysis were biologically interpreted using KEGG and Reactome pathway (J).

The workflow of the proposed framework is shown in Figure 1. First, coupled radiological images, gene expression, data, and clinical information (NSCLC-Radiogenomics dataset) were collected from publicly available datasets The Cancer Imaging Archive (TCIA) and GEO,[47] while The Cancer Genome Atlas (TCGA)-LUAD and TCGA-LUSC datasets were collected from TCGA repository[48,49] (Figure 1A). The data were then preprocessed (Figure 1B), and feature engineering was performed to select the features to be fed to the deep neural network models (Figures 1C and 1D). The sparse autoencoder-based models were designed to incorporate biological knowledge into the model and learn from small-sample-size radiological images, gene expression, and clinical information. The models were trained using a nested cross-validation approach (Figure 1F), where the inner loops were used to tune the hyperparameters and the outer loops were used to validate and evaluate the models' performance (Figures 1G and 1H). We used Shapley additive explanation (SHAP) values,[50] Grad-CAM,[51] and pathway interpretation to interpret these models and identify important prognostic biomarkers and pathways for high-risk categorized patients, with the goal of elucidating the biological significance of the proposed models (Figures 1I and 1J) (see STAR Methods for details).

RESULTS

Our goal was to develop and test biologically interpretable deep neural networks with robust performance on small datasets. To achieve this, we developed two deep-learning architectures, H-VAE-Cox (Figure 2) and XAT-VAE-Cox (Figure 3), for the intermediate integration of multi-dimensional radiological images, high-dimensional gene expression, and clinical data along with biological pathway knowledge for precise survival prediction. The performance was evaluated using the concordance index (C-index).[52]

Firstly, the tumor regions from CT-scan images were segmented using the U-Net architecture (Figure S1A) to extract the region of interest (ROchanI). The tumor location and size were determined via segmentation. A K-fold cross-validation approach was adopted to train, validate, and test the model, while the dice loss[53] and mean intersection over union were used to assess the model performance. The U-Net architecture achieved outstanding performance for tumor segmentation. The K-fold cross-validation resulted in a validation loss of 0.083 ± 0.017 (where 0.083 is the mean and 0.017 is the standard deviation) and a mean intersection over union of 0.901 ± 0.024. The trained model was then used to segment the tumor CT-scan

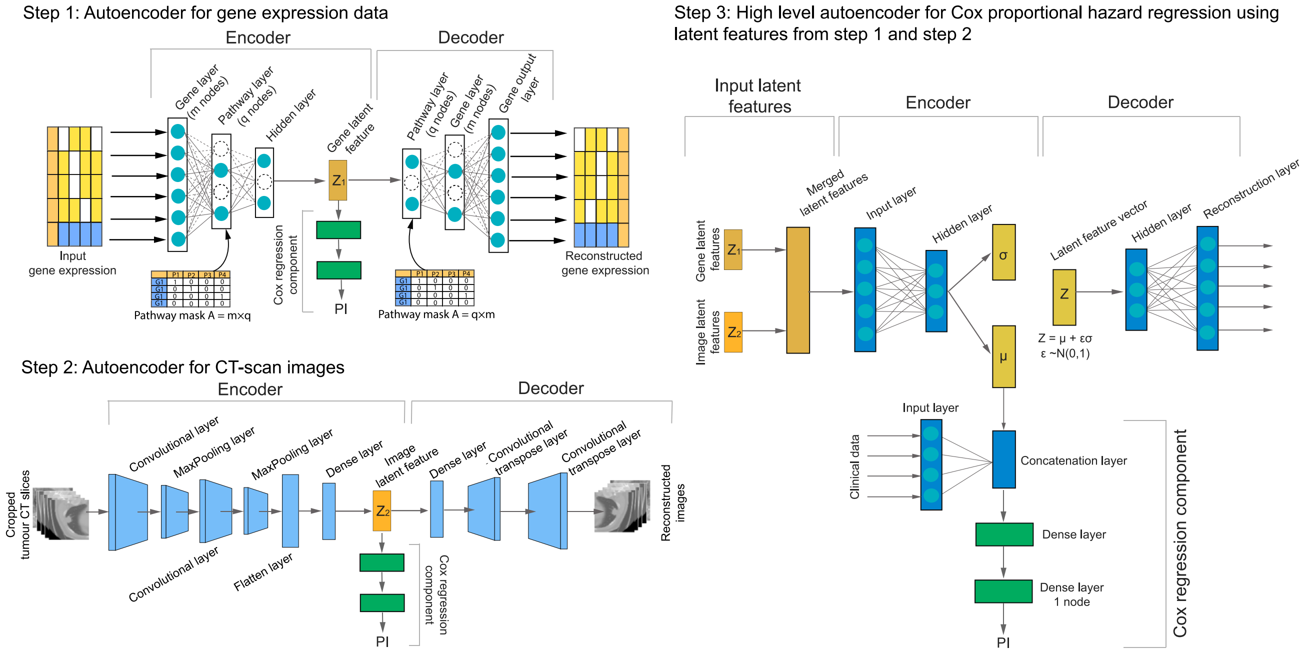

Figure 2. H-VAE-Cox: Hierarchical variational autoencoder-based Cox model

The latent features from the gene sparse autoencoder (step 1) and the image autoencoder (step 2) are fed into a high-level autoencoder (step 3) along with clinical data to estimate the PI. A sparse connection is created between the gene and pathway layers of the gene autoencoder (step 1) where a binary pathway mask matrix is fed into the pathway layer. Steps 1 and 2 are used for two purposes: (1) generate lower-dimensional latent features from each data modality, and (2) estimate the survival prediction for gene expression and image data individually, with the pathway mask injecting biological pathway knowledge into the learning process.

slices that had not yet been segmented, including CT-scan slices from TCGA-LUAD and TCGA-LUSC cohorts. The tumor region was then cropped using the OpenCV library[54] to obtain the tumor region as well as the surrounding information.

After preprocessing and extracting the ROI from CT-scan images, we trained H-VAE-Cox and XAT-VAE-Cox using the cropped tumor images along with preprocessed gene expression and clinical data for survival prediction.

H-VAE-Cox

We implemented H-VAE-Cox using a multimodal architecture and hierarchical integration approaches for combining multiple individual modalities. As depicted in Figure 2, the prognostic index (PI) of NSCLC patients was estimated using CT-scan images, gene expression, and clinical data in three steps. The H-VAE-Cox model is based on a multimodal architecture and hierarchical integration approaches, where lower-level autoencoders are used to generate low-dimensional latent features from images and gene expression data separately. Specifically, independently supervised autoencoders were designed for each data type, where the gene sparse autoencoder generates the latent features from gene expression data and the convolutional neural network (CNN)-based image autoencoder generates the latent features from images.

In order to generate lower-dimensional latent features associated with survival prediction, we designed supervised autoencoders in which a Cox neural network appended to the autoencoder bottleneck layer predicts the PI. Hence, the latent features generated from the supervised autoencoders are closely associated with the survival prediction and are also capable of being reconstructed to represent the original data.

The lower-level autoencoders (Figure 2, step 1 and step 2) are used for two purposes: (1) estimate the PI and perform the survival prediction for each independent data type (i.e., gene expression data and images separately) and (2) generate lower-dimensional latent features from gene expression and image data. These latent features generated from the gene sparse autoencoder and the image autoencoder were then integrated to form an integrated input feature for the high-level β-variational autoencoder (step 3).

The design of the H-VAE-Cox model architecture has the benefit of being a modular architecture in which a separate autoencoder is fitted on each modality independently to create low-dimensional features, ensuring that no information is lost from each modality during dimensionality reduction. In addition, due to its modularity, this architecture can be easily extended to add other omics data by adding independent modalities without having to change the entire architecture. Furthermore, since each lower-level autoencoder is trained independently, H-VAE-Cox requires less computational power compared to XAT-VAE-Cox.

XAT-VAE-Cox

For the second architecture, a cross-attention-based β-variational sparse autoencoder Cox model, XAT-VAE-Cox (Figure 3), was

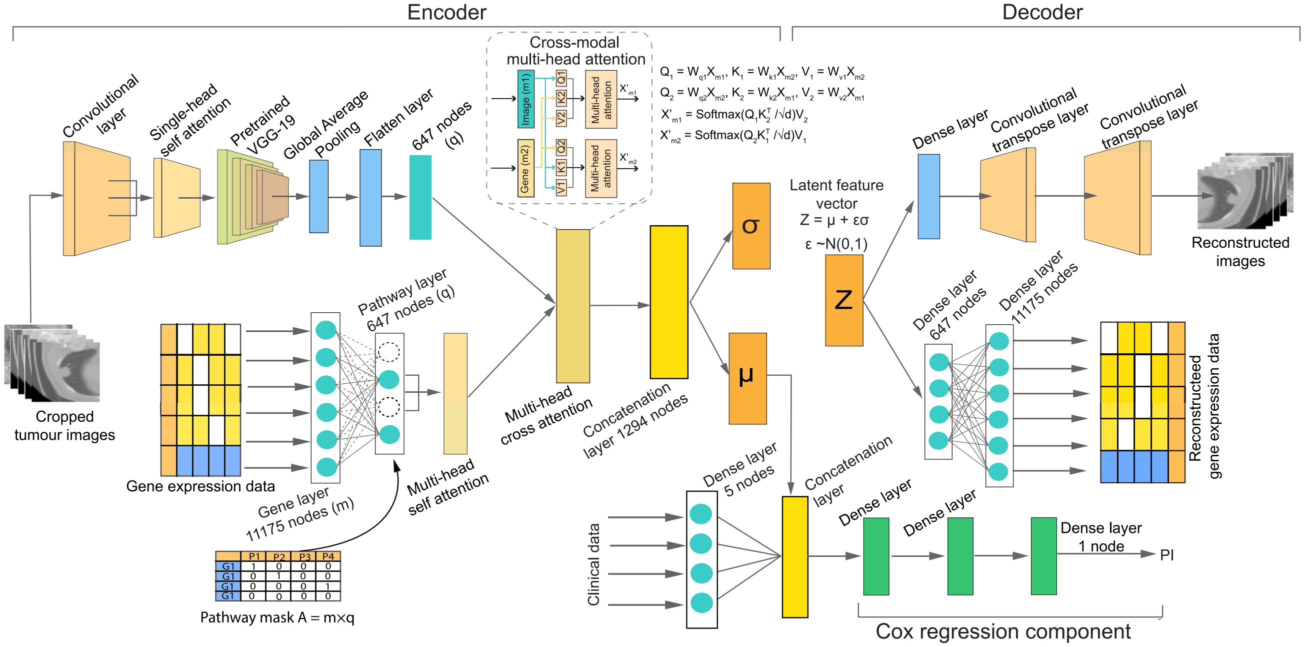

Figure 3. XAT-VAE-Cox: Cross-attention-based sparse variational autoencoder-based Cox model

The β-variational autoencoder architecture consists of encoder and decoder phases made from convolutional layers and dense layers for image and gene expression data. A sparse connection is created between gene and pathway layers, where a binary pathway mask matrix is fed into the pathway layer, adding biological knowledge to the network. The features from image and gene modalities are then fed to the multi-head self-attention layer, followed by the multi-head cross-attention layer to capture the cross-modality features. The latent vector μ is linked to the Cox regression component, which concatenates the latent vector and clinical features to estimate the PI.

designed to integrate CT-scan images and gene expression data using a single framework rather than independent autoencoders for each data type. In this model, both images and gene expression data were fed into a cross-attention-based autoencoder, where each modality was connected to the multi-head self-attention layer, followed by a cross-attention layer, to generate a low-dimensional Gaussian distribution $N(\mu, \sigma)$ of the latent feature z. The attention mechanism enables the network to focus on the most relevant features by assigning varying importance to distinct input features. The self-attention layer after the pathway layer in the gene modality highlights the important genes connected to the pathways, while the self-attention layer in the imaging modality helps the network to focus on the regions of an image that are most informative for PI estimation. The cross-attention mechanism helps the model establish cross-modality communication between the images and gene expression, improving the generation of the latent representation.

The latent vector μ and the clinical data are input to the subsequent Cox regression component. The encoder output vector μ was concatenated to the Cox proportional hazard layer, while the decoder reconstructed the images and gene expression data from a homogeneous latent representation. While training the model, the Cox regression component encouraged the network to develop latent representations capable of not only adequately reconstructing the input sample but also predicting the hazard ratio for survival analysis. The primary advantage of this architecture is that the attention mechanism focuses on the important features (both from genes and images) for the survival estimation, which not only improves the robustness of the model but also helps in biomarker identification. Furthermore, this autoencoder generates a single low-dimensional latent vector from both images and gene expression data, and the decoder of this model can reconstruct images and gene expression from that single latent vector.

Survival prediction with H-VAE-Cox and XAT-VAE-Cox outperforms other models

We used radiological imaging, gene expression, and clinical data to stratify NSCLC patients into risk groups based on the PI estimated by the two proposed models. The risk scores (i.e., PI) from gene expression and images were estimated independently using the two low-level autoencoders for H-VAE-Cox, i.e., the gene sparse autoencoder and the image autoencoder (Figure 2, steps 1 and 2 respectively). Then, the Cox regression component was configured to estimate the PI using clinical data only. In addition, the XAT-VAE-Cox model was also configured for survival prediction using two data modalities (i.e., images with clinical data, and gene expression with clinical data). For each of these experiments, the samples were categorized into two risk groups (high-risk and low-risk) by using the median value of the PI as a threshold (high risk if PI > median, low risk otherwise). The survival distributions of high- and low-risk groups of individuals were compared using the log rank test. Kaplan-Meier (KM) curves were then plotted to visualize the results using the survival package in R.[55]

As illustrated in Figures 4A–4G, KM curves were evaluated for high- and low-risk grouped patients using: (A) clinical data (Cox

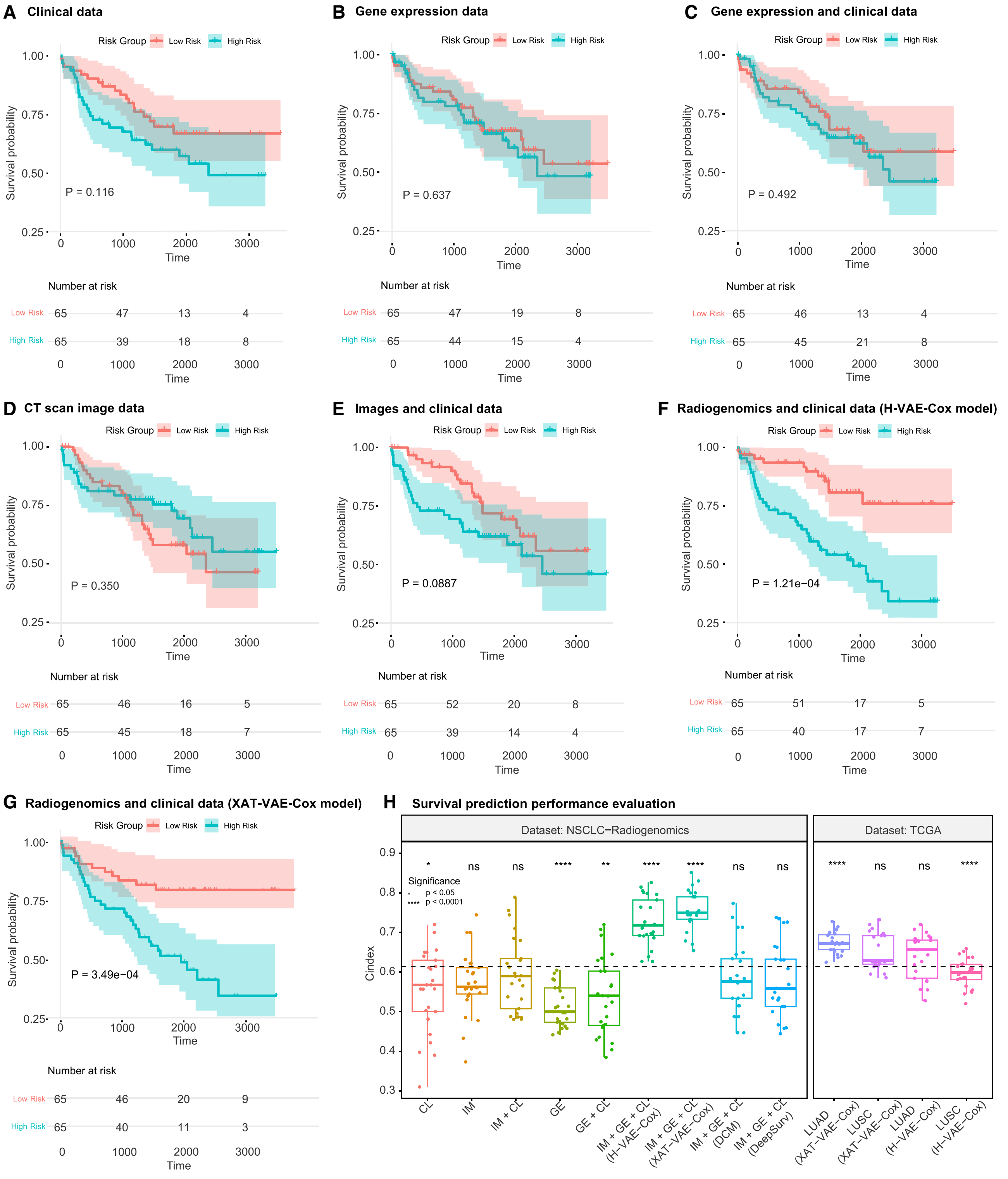

Figure 4. KM curves using radiological images, gene expression, clinical data, and survival risk groups

(A) KM curves for clinical data only (using the Cox regression component).

(B) KM curves for gene expression data (using the gene sparse autoencoder Cox model).

(C) KM curves for gene expression with clinical (using XAT-VAE-Cox).

(D) KM curves for images only (using the image autoencoder Cox model).

(legend continued on next page)

regression component), (B) gene expression data only (gene sparse autoencoder), (C) gene expression and clinical data (XAT-VAE-Cox), (D) CT-scan images only (image autoencoder), (E) images and clinical data (XAT-VAE-Cox), and (F and G) the integrated features from images, gene expression, and clinical data as input for H-VAE-Cox and XAT-VAE-Cox, respectively. When comparing high- and low-risk survival groups using only clinical data (Figure 4A), only gene expression data (Figure 4B), or merging gene expression data with clinical data (Figure 4C), there was no statistically significant difference ($p > 0.05$) between the risk groups. Even if using only imaging data (Figure 4D), or merging images with clinical data (Figure 4E), no statistically significant difference was observed between the risk groups ($p > 0.05$). This suggests that using only clinical information, gene expression, or imaging data is insufficient to precisely stratify patients into risk groups. The most significant p values were associated with the integration of radiogenomics and clinical data through the proposed H-VAE-Cox ($p = 1.21e-4$) and XAT-VAE-Cox ($p = 3.49e-4$), as shown in Figures 4F and 4G, respectively. Overall, the KM curves show that employing the integrated features from images, gene expression, and clinical data using both H-VAE-Cox and XAT-VAE-Cox outperforms all the other models in terms of both survival prediction and patient risk group stratification.

The performance of the integration of three data modalities (imaging, gene expression, and clinical data) by the H-VAE-Cox and XAT-VAE-Cox models was then compared to that of the single and two data modalities (Figure 4H). The C-index estimated when using only clinical data for the Cox regression component was 0.55 ± 0.10, while the C-index estimated when using only gene expression data for the low-level gene sparse autoencoder was 0.51 ± 0.05. Similarly, the C-index estimated when using only image data for the image autoencoder was 0.57 ± 0.08. Then, two data modalities (i.e., gene expression with clinical data and images with clinical data) were integrated to estimate the PI. The model achieved a C-index of 0.54 ± 0.09 when using only gene expression with clinical data and a C-index of 0.59 ± 0.09 when integrating images with clinical data.

We then asked whether we could elucidate the advantage of integrating image, gene expression, and clinical data for the survival prediction task. To this end, three data modalities were integrated to estimate the PI. A significant improvement in the survival prediction was observed by integrating the features from images, gene expression, and clinical data in the H-VAE-Cox model (Figure 2 step 3), with a C-index of 0.73 ± 0.06. Moreover, the XAT-VAE-Cox model (Figure 3) estimated the PI from images, gene expression, and clinical data more precisely, with a C-index of 0.76 ± 0.05. These results confirm that compared to single or two data modalities, the integration of three data modalities (CT-scan images, gene expression, and clinical data) improves survival prediction. Figure 4H illustrates the performance of H-VAE-Cox and XAT-VAE-Cox using CT-scan images, gene expression, and clinical data. The statistical test assesses the difference between the performance of the models across all the possible combinations of input data, showing that there is a significantly improved performance by the H-VAE-Cox and XAT-VAE-Cox models compared to other models.

To compare our proposed approach with existing techniques, in Figure 4H we compare the performance of both the proposed architectures with DeepSurv[35] using the PyCox library[56] and deep Cox mixture (DCM).[38] While these models were originally designed for low-dimensional high-sample size unimodality data, the multimodal CT-scan images, gene expression, and clinical data needed to be preprocessed and transformed into lower dimensions prior to training these models. Therefore, the latent features from the gene sparse autoencoder and image autoencoder (Figure 2, step 1 and step 2, respectively) and clinical information were fed into the DeepSurv and DCM models. In particular, the latent representation, each of size 500 features, generated from these autoencoders, along with clinical features, were concatenated to form an input feature of size 1,006. This concatenated feature was then fed into the DeepSurv and DCM models to predict the survival of NSCLC patients. To ensure consistency and robustness, each model was trained five times using a nested cross-validation approach. The DeepSurv model obtained a C-index of 0.58 ± 0.09, 0.50 ± 0.12, and 0.51 ± 0.18 on NSCLC-Radiogenomics, TCGA-LUAD, and TCGA-LUSC datasets, respectively, while the DCM model obtained a C-index of 0.59 ± 0.095, 0.54 ± 0.05, and 0.51 ± 0.11 on NSCLC-Radiogenomics, TCGA-LUAD, and TCGA-LUSC datasets, respectively. When compared to the DeepSurv and DCM models, both our proposed autoencoder-based architectures achieved a significantly higher survival prediction accuracy using images, gene expression, and clinical information.

We then asked whether the proposed models are robust when applied to unseen external datasets and are therefore suitable for use within entirely new prediction tasks. The models trained on 130 samples from the NSCLC-Radiogenomics dataset were therefore evaluated on two cohorts from the TCGA repository (TCGA-LUAD and TCGA-LUSC). It was observed that the attention-based XAT-VAE-Cox model trained on a small sample was robust to make predictions on unseen TCGA-LUAD and TCGA-LUSC datasets (0.68 ± 0.03 and 0.65 ± 0.05, respectively) compared to H-VAE-Cox model on TCGA-LUAD and TCGA-LUSC datasets (0.64 ± 0.06 and 0.60 ± 0.04). The performance of the proposed models on NSCLC-Radiogenomics data, TCGA datasets, and the performance of DeepSurv and DCM models on NSCLC-Radiogenomics data was also evaluated using additional two metrics, concordance index inverse probability of censoring weighting (C-index IPCW), and cumulative dynamic area under the curve (AUC), as shown in Table S1. The evaluation using these metrics further revealed the robustness

(E) KM curves for images and clinical data (using XAT-VAE-Cox).

(F and G) KM curves for XAT-VAE-Cox and H-VAE-Cox using images, gene expression, and clinical features. Radiogenomics (images + gene expression) along with clinical features show significant survival differences ($p < 0.05$) between the risk groups.

(H) Statistical and experimental results of H-VAE-Cox and XAT-VAE-Cox in terms of C-index. (IM, imaging; GE, gene expression; CL, clinical data). Statistical significance is denoted by * for adjusted $p < 0.05$, ** for adjusted $p < 0.01$, *** for adjusted $p < 0.001$ and ns for non significant.

of the proposed approach, with the XAT-VAE-Cox model outperforming all other models.

Model interpretation

The proposed H-VAE-Cox and XAT-VAE-Cox models integrate the features from images, gene expression, and clinical data to estimate patient-specific PIs. As a result, it is critical to interpret both models to identify the cancer-related features that play a key role in survival prediction and to identify the best model to choose in each scenario. SHAP values[50] were used for interpretation due to their several properties, which made them suitable for our investigation. First and foremost, SHAP values are model agnostic. This means they can be applied to any model, which was critical for our methods based on custom-designed architectures. Furthermore, SHAP values exhibit properties of local accuracy, missingness, and consistency that are not simultaneously present in other explainability approaches.

We computed SHAP values for both the proposed models (H-VAE-Cox and XAT-VAE-Cox) focusing on high-risk categorized patients. In particular, we used the GradientExplainer function from the SHAP library with high-risk samples. Since our models used three types of input data (images, gene expression, and clinical data) to estimate the PI, it was important to identify relevant features from each data type. The low-dimensional latent features encoded by the gene sparse autoencoder and image autoencoder, along with clinical data, were input into the SHAP Explainer for H-VAE-Cox, while the images, gene expression, and clinical data were input into the SHAP Explainer for XAT-VAE-Cox. The SHAP representations for both models for the three input data modalities were then analyzed to understand the impact of the input features on the model predictions.

As the latent features generated from low-level autoencoders were fed into the high-level autoencoder-based Cox model (Figure 2 step 3) for H-VAE-Cox, the SHAP interpretation of the high-level autoencoder-based Cox model identified the important gene and image latent features (Figure 5A). We noted that, to explore the important features and identify the markers from each omic data influencing the prediction, the SHAP value generated for H-VAE-Cox had to be decoded by the gene sparse autoencoder and image autoencoder in order to generate SHAP representations for the original gene expression and multidimensional images. Hence, for further biological interpretation and identification of significant genes and ROI for high-risk survival prediction, the SHAP values generated from the high-level autoencoder-based Cox model were decoded using the low-level autoencoders (gene sparse autoencoder and image autoencoder; steps 1 and 2 in Figure 2).

The SHAP representation of the gene sparse autoencoder identified the important genes impacting the prediction for high-risk patients (Figure 5B), which was further analyzed to understand if these genes have any biological significance for NSCLC prognosis. Similarly, the SHAP representation from the image autoencoder identified the important regions in images influencing the prediction. The SHAP importance was overlaid over the original images to identify regions in CT-scan images significant for the model output (Figure 5D). The SHAP image plots clearly illustrated that the tumor region and the areas near the tumor regions were identified as the important regions in the CT-scan images for the model prediction.

Unlike H-VAE-Cox, the XAT-VAE-Cox model directly used the images, gene expression, and clinical data to train the model. As a result, the SHAP interpretation for XAT-VAE-Cox was able to use the images, gene expression, and clinical data directly and identify the important features from each omic dataset. The SHAP representation for the gene expression data identified the important genes influencing the model prediction (Figure 5C), which was also further analyzed to find the biological relevance with NSCLC. The list of genes sorted by SHAP values for both models is provided in Tables S3 and S4. Importantly, the tumor regions in the radiological images also contributed toward the estimation of PI, as demonstrated by the SHAP importance overlayed over images (Figure 5E). The SHAP interpretation of the clinical feature for both models highlighted recurrence status as an important clinical feature for high-risk grouped patients (Figures 5F and 5G).

To quantify the contribution of each data modality for both the proposed models, the multimodality score was estimated based on SHAP values (see experimental design and model evaluation). Specifically, the multimodality score determines the contribution of each modality toward the estimation of PI. It was found that the features from the images or the gene expression data did not overshadow each other, as the multimodality score for the H-VAE-Cox model was estimated as 0.36, 0.16, and 0.48 for images, gene expression, and clinical data respectively. Similarly, the multimodality score for the XAT-VAE-Cox model was estimated as 0.25, 0.3, and 0.45 for images, gene expression, and clinical data, respectively.

The interpretability of the imaging modality in the proposed multimodal models was further improved by applying Grad-CAM, where the activation map was visualized in the last CNN layer of the image autoencoder for the H-VAE-Cox model, and in the self-attention layer of the imaging modality in the XAT-VAE-Cox model. As shown in Figures 5D and 5E, this analysis highlighted the core tumor regions as important features for the estimation of the PI. The highlighted regions in the images were further validated by comparing the region with the segmentation ground truth. It was observed that the Grad-CAM-based interpretation of the imaging modality in the XAT-VAE-Cox model highlighted the tumor region more precisely, compared to the H-VAE-Cox model.

Clinical validation of the Grad-CAM-based interpretation

The Grad-CAM-based interpretation demonstrated that the cross-attention mechanism in the XAT-VAE-Cox model better learned the cross-modality interaction between CT-scan images and gene expression, compared to the H-VAE-Cox model, where each modality was trained independently to generate a latent representation. Thus, the heatmap generated by the Grad-CAM-based interpretation of the XAT-VAE-Cox model for the imaging modality was validated by the radiologists in our team. Specifically, they examined sample images from 60 high-risk-categorized patients and confirmed that the XAT-VAE-Cox model effectively identified key areas in the images.

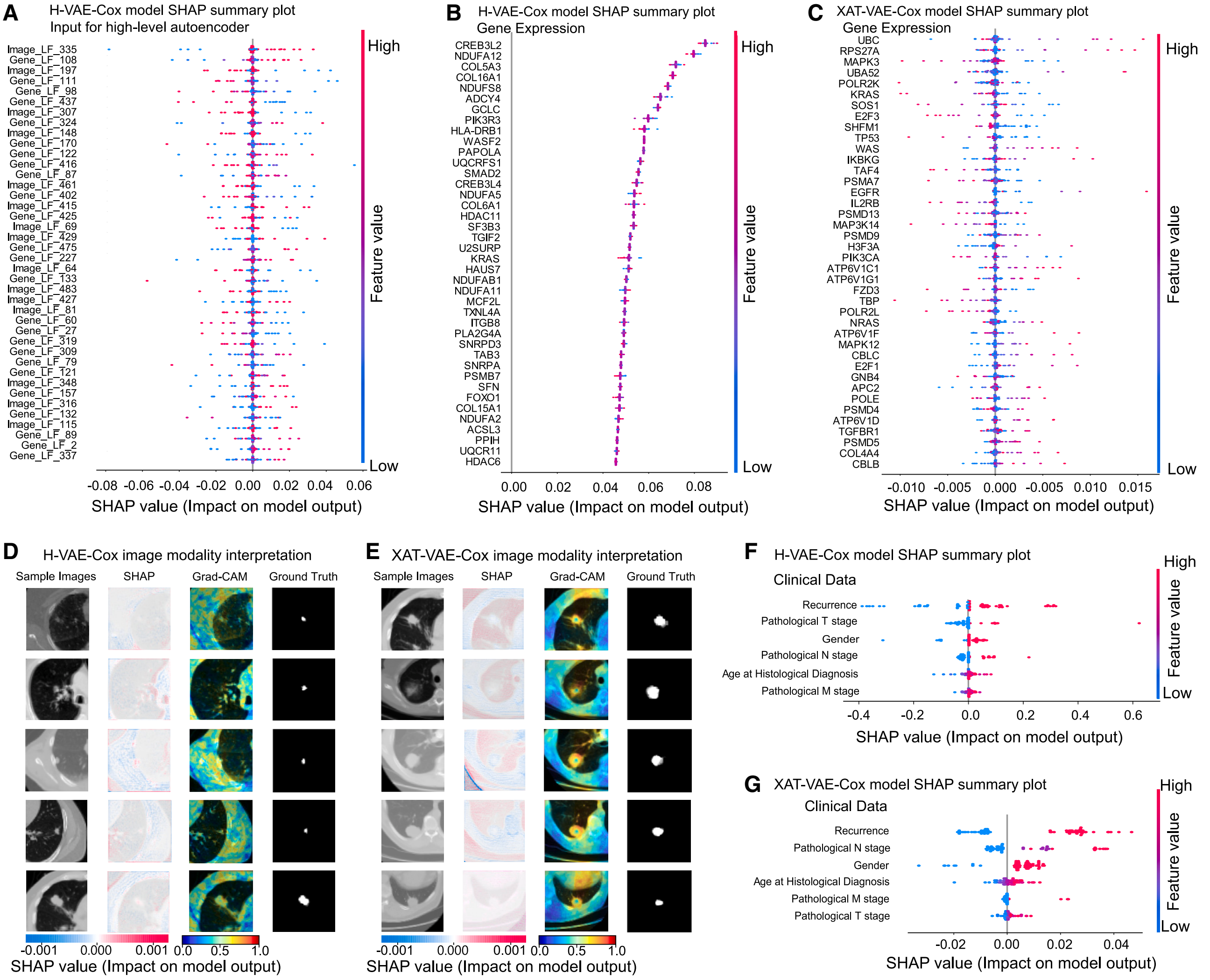

H Summary of radiologists' response for Grad-CAM-highlighted images

Grad-CAM evaluation criterion	Score (Count of images) 1	2	3	4	
1. For each specific scan, are the highlighted regions tumour regions?	Yes (278 images)				No (22 images)
2. For each specific scan, how well can the model identify tumour regions?	-	139	122	17	-
3. For each specific scan, are all the highlighted regions related to tumour regions?	-	224	53	1	-

I Sample Grad-CAM-highlighted images, scored by radiologists

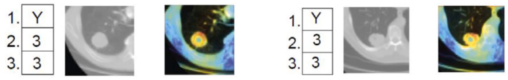

Figure 5. Model interpretation using SHAP values and Grad-CAM

(A–G) Summary plots for SHAP values are used to interpret the H-VAE-Cox and XAT-VAE-Cox models. Each dot represents an instance of the dataset (i.e., a patient in the high-risk group). The x axis shows the SHAP value, while the y axis shows the features ranked by their contribution to the model output, as determined by the average of Shapley values. Important features are positioned higher on the y axis. Instances with high-value features are in red, while instances with low-value features are in blue. The summary plot in (A) illustrates the SHAP importance of gene and image latent features fed into the high-level autoencoder in H-VAE-Cox. (B) and (C) report the summary plots for gene expression for H-VAE-Cox and XAT-VAE-Cox, respectively, used to identify the most significant genes for survival prediction. In (B), SHAP values for genes were obtained by decoding the SHAP value for gene latent features in H-VAE-Cox (A). (D) and (E) present the SHAP image plot, Grad-CAM visualization, and tumor ground truth to interpret the H-VAE-Cox and XAT-VAE-Cox models' input images; in (D), SHAP values for images were obtained by decoding the SHAP value for image latent features in H-VAE-Cox (A). The x axis shows the SHAP value, while each row represents a sample image. The impact of the regions of the images on the model output for the individual patient is depicted by the color of dots plotted over the images. The red color represents important regions with a high impact on the model output, while the blue color represents less important regions. The Grad-CAM heatmap highlights the regions in the images that contribute to the estimation of PI. When comparing the highlighted region with the tumor ground truth, XAT-VAE-Cox is more accurate than H-VAE-Cox in emphasizing the tumor region. (F) and (G) present the summary plots used to identify the clinical features with the highest impact on the model output for both H-VAE-Cox and XAT-VAE-Cox.

(H) Summary of responses from radiologists for Grad-CAM-highlighted images, where the radiologists validated the images based on three questions (evaluation criterion). Out of 300 images, Grad-CAM highlighted tumor regions for 278 images, while for the remaining 22 images the Grad-CAM-highlighted regions were not tumorous.

(I) Sample of Grad-CAM-highlighted images validated by the radiologists. Each image was scored based on the questionnaire as shown in (H).

The images were evaluated and scored based on the following three questions.

- For each scan, are the highlighted regions tumor regions? (Yes or No).
- For each scan, how well can the model identify tumor regions? (Score 1–4) Scores: (1), tumor regions are not identified at all; (2) tumor regions are somewhere identified; (3) most of the tumor regions are correctly identified; (4) all the tumor regions are correctly identified.
- For each scan, are all the highlighted regions (yellow/red) related to tumor regions? Score 1 to 4: (1) highlighted regions (yellow/red) do not make sense; (2) only some of the highlighted (yellow/red) regions are correct; (3) most of the highlighted regions (yellow/red) are related to tumors; (4) all the highlighted regions (yellow/red) are related to the tumor.

As summarized in Figure 5H, among 300 images, Grad-CAM highlighted tumor lesions in 278 images. It was interesting to observe that Grad-CAM highlighted most of the tumor regions correctly in 122 images. Furthermore, in 139 images, tumor regions along with nearby regions were correctly highlighted. Interestingly, for 17 images, all the tumor regions were correctly highlighted by the Grad-CAM-based interpretation. In response to the third question, it was observed that, for 224 images, Grad-CAM highlighted the tumor region along with nearby regions, vessels, heart, and even chest wall, while, for 53 images, most of the highlighted regions (yellow/red) are related to tumors.

It was also noted that the periphery of the tumor was highlighted, which is important for radiologists as it helps find the tumor contour (Figure 5I). However, some regions adjacent to the lesion were also highlighted, which may not be of interest. To refine the image analysis and resolve these issues, we believe a combined analysis of Grad-CAM and tumor segmentation should be used. Moreover, some samples had movement or breathing artifacts in the CT-scan images, which hindered the ability to highlight tumor areas, resulting in false-positive cases. Such images could also impair the radiologist's judgment and, in such cases, the radiologists would suggest re-imaging or using alternative imaging techniques, such as positron emission tomography (PET) scan.

It was further observed that some images showed lesions that could be benign or indeterminate, requiring follow-up examinations to monitor their growth. Overall, albeit with some false-positive cases, the radiologists strongly agreed that the Grad-CAM-highlighted regions were of interest and would constitute valuable support to their decision-making process. They also confirmed that the model correctly identified the lesions in 93% of the images, suggesting that the model learned to focus on relevant regions. The Grad-CAM-highlighted CT-scan images validated by the radiologists are provided as supplemental information.

Biological interpretation

In order to further biologically interpret the proposed models and explore the significant biological processes characterizing high-risk patients, the top 40 important genes identified by the SHAP interpretation of both models (Figures 5B and 5C) were investigated for KEGG pathways and Gene Ontology (GO) (Figure 6). Then, the study was extended by selecting the top 15% (i.e., 1,714) genes, sorted by SHAP value, to perform a KEGG and Reactome pathway analysis. Additionally, we performed gene set enrichment analysis (GSEA) for two KEGG pathways (i.e., "NSCLC KEGG pathway" and "pathways for cancer") using the gene expression dataset and the high- and low-risk survival groups estimated by the models (Figure 7).

Identification of potential biomarkers through enrichment analysis of SHAP-identified genes

The KEGG pathways and functional GO in the context of NSCLC were investigated by analyzing the top 40 genes from both models, as determined by the SHAP interpretation (Figures 5B and 5C).

To assess the relevance of these markers in predicting a poor prognosis for NSCLC, a KEGG pathway analysis was conducted using DAVID (https://david.ncifcrf.gov/) (Tables S5 and S6). Firstly, the pathway analysis was performed for the H-VAE-Cox model (Figure 6A), where the results revealed that 12 genes (GCLC, NDUFS8, NDUFA11, NDUFA5, NDUFA12, NDUFAB1, NDUFA2, ADCY4, PLA2G4A, UQCRFS1, UQCR11, and ACSL3) were actively involved in the metabolic pathway, while eight genes (NDUFS8, NDUFA11, NDUFA5, NDUFA12, NDUFAB1, NDUFA2, UQCRFS1, and UQCR11) were associated with oxidative phosphorylation pathway. Mitochondrial oxidative phosphorylation, i.e., aerobic mitochondrial respiration, plays a crucial role in providing energy to cancer cells, including NSCLC. The markers of mitochondrial biogenesis and components of oxidative phosphorylation complexes are important biomarkers for lower survival in NSCLC patients.[57]

Furthermore, six genes (CREB3L4, COL6A1, CREB3L2, ITGB8, PIK3R3, and KRAS) were found to play a role in the PI3K-Akt signaling pathway. Dysregulation of PI3K-Akt signaling pathway activates cellular stimuli and regulates fundamental cellular functions such as transcription, translation, proliferation, growth, and survival of NSCLC.[58] Moreover, three genes (PIK3R3, PLA2G4A, and KRAS) were also linked to the vascular endothelial growth factor (VEGF) signaling pathway, which plays a crucial role in angiogenesis. Angiogenesis is essential for tumor growth and metastasis, and the VEGF signaling pathway is one of the most important pathways involved in this process. In particular, the PIK3R3 gene encodes a regulatory subunit of phosphatidylinositol 3-kinase (PI3K), which is a key mediator of the VEGF signaling pathway and PI3K-Akt signaling pathway. PLA2G4A encodes an enzyme that catalyzes the hydrolysis of membrane phospholipids to release arachidonic acid (AA). AA metabolism has been implicated in various cellular processes, including inflammation and cancer progression. KRAS encodes a protein involved in cell signaling pathways, including the VEGF and PI3K-Akt signaling pathways. Mutations in KRAS have been associated with various cancers, including NSCLC.[59,60] Taken together, the KEGG pathway analysis of important genes identified by SHAP interpretation of the H-VAE-Cox model demonstrated their relevance in the poor prognosis of NSCLC.

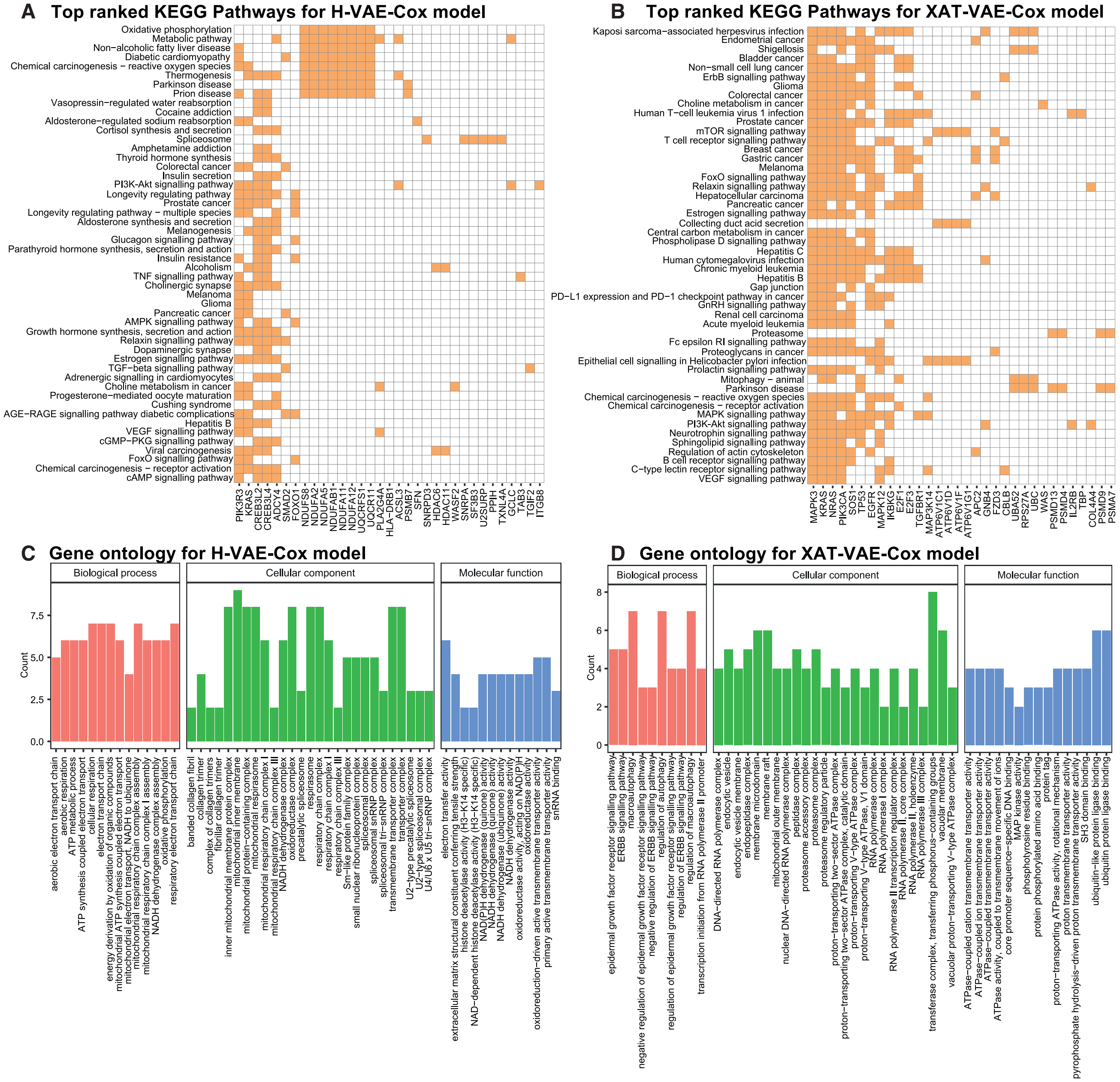

Figure 6. KEGG pathways and functional GO analysis for the top 40 genes identified by the SHAP interpretation of H-VAE-Cox and XAT-VAE-Cox

(A and B) Top 50 KEGG pathways for the important genes identified by H-VAE-Cox and XAT-VAE-Cox models, respectively, where rows represent the KEGG pathways and columns represent the genes. The brown color on the heatmap illustrates the association of genes with the KEGG pathway.

(C and D) GO of top 40 genes from SHAP interpretation of H-VAE-Cox and XAT-VAE-Cox models. The important genes were significantly enhanced in biological process (BP), cellular component (CC), and molecular function (MF) with adjusted $p < 0.005$.

Then, a KEGG pathway analysis was performed on the top 40 genes identified by the SHAP interpretation of the XAT-VAE-Cox model (Figure 6B). Out of 40 genes, nine genes (NRAS, PIK3CA, E2F1, KRAS, E2F3, SOS1, TP53, EGFR, and MAPK3) were involved in the NSCLC pathway. This pathway is a complex network of genes and signaling pathways involved in the development and progression of NSCLC.[61] Mutations in these genes can lead to the activation of various signaling pathways such as PI3K-Akt, VEGF, and MAPK. NRAS and KRAS are members of the RAS family of oncogenes that play a crucial role in regulating cell growth and differentiation.

The SHAP interpretation revealed 10 genes (NRAS, KRAS, IKBKG, SOS1, TP53, MAP3K14, EGFR, TGFBR1, MAPK12, and MAPK3) that have been associated with activation of the

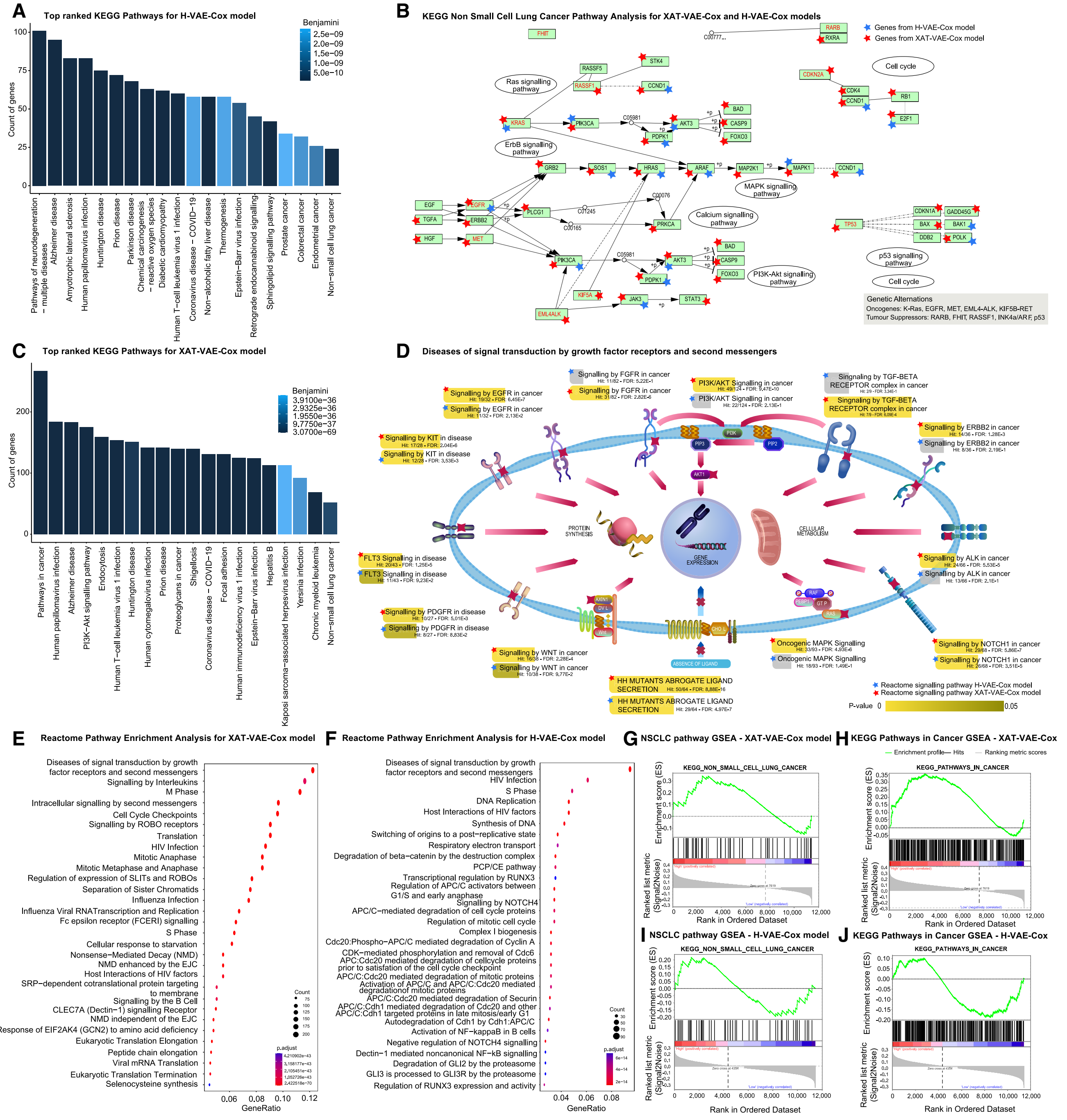

Figure 7. KEGG and Reactome pathway analysis for H-VAE-Cox and XAT-VAE-Cox using the top 15% significant genes sorted by SHAP values

(A–C) The top 20 significant KEGG pathways were identified using H-VAE-Cox and XAT-VAE-Cox, respectively, where Benjamini values were used to assess the significance of each pathway.

(B) The NSCLC KEGG pathway was further analyzed using the top 15% significant genes identified by H-VAE-Cox and XAT-VAE-Cox. Gene names in red color indicate oncogenes and tumor suppressor genes significant for NSCLC. Blue stars represent significant genes identified by H-VAE-Cox, and red stars represent significant genes selected by XAT-VAE-Cox.

(D) Significance of signaling pathways in the "diseases of signal transduction via growth factor receptors and second messengers" Reactome pathway for XAT-VAE-Cox and H-VAE-Cox.

(legend continued on next page)

MAPK signaling pathway. These mutations have been linked to the activation of the MAPK signaling pathway, which plays a role in regulating the growth and differentiation of NSCLC cells.[62] E2F1 and E2F3 are transcription factors that play a role in regulating cell cycle progression. The overexpression of these genes has been associated with the development of NSCLC.[63] SOS1 is a guanine nucleotide exchange factor that plays a role in activating RAS proteins. Mutations in this gene have been linked to the activation of the RAS-MAPK signaling pathway.[64] TP53 is a tumor suppressor gene that helps regulate cell cycle progression and apoptosis. EGFR is a receptor tyrosine kinase that regulates cell growth and differentiation. MAPK3 is a member of the MAP kinase family that also contributed to regulating cell growth.[65,66]

Furthermore, 16 genes (APC2, FZD3, EGFR, TGFBR1, NRAS, PIK3CA, COL4A4, IL2RB, E2F1, GNB4, KRAS, E2F3, IKBKG, SOS1, TP53, and MAPK3) are involved in pathways in cancer, while 11 genes (NRAS, PIK3CA, COL4A4, IL2RB, GNB4, KRAS, IKBKG, SOS1, TP53, EGFR, and MAPK3) are involved in PI3K-Akt signaling pathway and five genes (NRAS, PIK3CA, KRAS, MAPK12, and MAPK3) are involved in VEGF signaling pathway, demonstrating that the important genes identified by the SHAP interpretation of XAT-VAE-Cox model are linked with poor prognosis of NSCLC.

Figure 6C presents the GO analysis, performed using the clusterprofiler R package,[67] of the top 40 genes from the SHAP interpretation of H-VAE-Cox model. The pathways by GO analysis were found to be related to the process of cellular respiration and oxidative phosphorylation. Oxidative phosphorylation consists of two components: the electron transport chain and ATP synthesis, which were also found to be enriched by GO analysis.[68] It was observed that the significant pathways identified by GO for biological processes are either part of or related to the electron transport chain or ATP synthesis. For instance, aerobic respiration, aerobic electron transport chain, and respiratory electron transport chain were found to be enriched in NSCLC. Similarly, other enriched pathways such as the ATP metabolic process and energy derivation by oxidation of organic compounds are general terms for the production of ATP by cellular respiration. Mitochondrial respiratory chain complex assembly and mitochondrial respiratory chain complex I assembly were also found to be enriched.[69] The relevance of these pathways in NSCLC is that they are essential for the survival and proliferation of NSCLC cells, as they provide them with the energy they need to grow and divide. These pathways are also potential targets for cancer therapy, as disrupting them could impair the energy metabolism of cancer cells and induce cell death.

Furthermore, the mitochondrial pathways related to cellular components were also found to be enriched. The mitochondrial respiratory chain complexes I and III, the NADH dehydrogenase complex, the oxidoreductase complex, and the respirasome are involved in the oxidative phosphorylation (OXPHOS) process, which generates ATP and reactive oxygen species (ROS) in the mitochondria. NSCLC cells can switch between OXPHOS and glycolysis depending on the availability of oxygen and nutrients, and this metabolic flexibility confers them an advantage in survival and adaptation.[70] Similarly, collagen pathways were also enriched, where collagen is the main component of the extracellular matrix (ECM) providing structural and biochemical support to the cells. Collagen plays a role in modulating the signaling and behavior of NSCLC cells, such as proliferation, migration, invasion, and angiogenesis.[71] Therefore, collagen pathways are relevant for the poor prognosis of NSCLC and may be targeted by anti-fibrotic or anti-angiogenic agents.

Similarly, Figure 6D demonstrates the GO analysis of the top 40 important genes from SHAP interpretation of the XAT-VAE-Cox model. The pathways identified enriched by GO analysis were found to be related to the regulation of cell growth, survival, differentiation, and death in NSCLC. The alterations in these biological processes are often associated with mutations or overexpression of the EGFR or the ErbB family of receptor tyrosine kinases (RTKs), which include EGFR, ErbB2, ErbB3, and ErbB4. For instance, the enriched EGFR signaling pathway regulates diverse cellular functions related to survival, growth, proliferation, and differentiation.[72] Similarly, the ErbB signaling pathway was also found to be enriched, which is activated by the binding of various ligands to the ErbB family of RTKs, which form homo- or heterodimers with each other. ErbB signaling is also frequently altered in NSCLC due to ErbB2 amplification or overexpression.[73] Macroautophagy is another significantly enriched pathway that is found to have a dual role in NSCLC, as it can either promote cell survival and adaptation under stress conditions or induce cell death and senescence under excessive or prolonged stress.[74] Another enriched pathway is the negative regulation of the EGFR signaling pathway. The EGFR signaling pathway is often dysregulated in NSCLC, leading to increased tumor growth and resistance to therapy. Therefore, negative regulation of the EGFR signaling pathway is considered a potential therapeutic strategy for NSCLC.[75]

The GO analysis also identified enriched cellular component-related pathways. For instance, dysregulation of DNA-directed RNA polymerase complex and RNA polymerase complex in NSCLC may alter gene expression and regulation. The mutations in the RNA polymerase II core complex can affect the transcription of tumor suppressor genes or oncogenes.[76] The GO analysis identified endocytic vesicle and endocytic vesicle membranes dysregulated in NSCLC, which are involved in receptor-mediated signaling.[77] Similarly, the enriched endopeptidase complex and peptidase complex are found to be altered in NSCLC, which play a role in protein degradation and processing.[78] The enriched mitochondrial outer membrane is involved in various cellular processes, such as apoptosis, metabolism, or oxidative stress, which can be altered in NSCLC.[79] Transferase complex is involved in various signaling pathways, such as the PI3K-Akt signaling pathway or the MAPK signaling pathway, which can be dysregulated in NSCLC.[80]

(E and F) Highly enriched Reactome pathways for XAT-VAE-Cox and H-VAE-Cox, where the Reactome pathways are displayed on the y axis and the gene ratio is represented on the x axis.
(G and H) GSEA results for XAT-VAE-Cox for ‘‘non-small-cell lung cancer’’ and ‘‘pathways in cancer’’ KEGG pathways.
(I and J) GSEA results for XAT-VAE-Cox for ‘‘Non-small-cell lung cancer’’ and ‘‘pathways in cancer’’ KEGG pathways.

The top genes were also found to be enriched in molecular functions associated with NSCLC. For instance, ATPase-coupled cation transmembrane transporter activity, ATPase-coupled ion transmembrane transporter activity, and ATPase-coupled transmembrane transporter activity are molecular functions that describe the ability of some proteins to use the energy of ATP hydrolysis to transport cations or ions across membranes. They are involved in maintaining the ion homeostasis and the electrochemical gradient of various cellular compartments, such as the cytosol, the mitochondria, the lysosomes, or the vacuoles, which can be altered in NSCLC.[81] The dysregulated activity of MAP kinase activity in NSCLC alters various cellular processes, such as cell proliferation, differentiation, survival, migration, and invasion.[82] Therefore, understanding these pathways could help to develop new strategies to treat NSCLC.

KEGG and Reactome pathway analysis for the top 15% genes

The study was subsequently extended by selecting the top 15% important genes identified by SHAP interpretation. For each model, significant pathways with p value or Benjamini value (i.e., adjusted p values) < 0.05 were examined using DAVID (https://david.ncifcrf.gov/). Figures 7A–7C demonstrates the top 20 significant KEGG pathways, while the complete list of significant KEGG pathways is provided in Tables S7 and S8. As illustrated in Figures 7A–7C, XAT-VAE-Cox identified a larger number of genes involved in the significantly enriched KEGG pathways compared to the H-VAE-Cox. Moreover, out of the top 1,714 important genes sorted by SHAP importance, 829 genes from H-VAE-Cox and 1,501 genes from XAT-VAE-Cox were associated with the KEGG pathways. Furthermore, the Benjamini values for the KEGG pathways linked with significant genes by H-VAE-Cox were higher than those obtained with XAT-VAE-Cox. While both models identified the significant genes and biological pathways responsible for NSCLC, our results suggest that XAT-VAE-Cox was able to learn more biological pathway knowledge than H-VAE-Cox.

Among the significantly enriched pathways, several pathways linked with NSCLC were identified. Dysregulation in the cell cycle is a characteristic of cancerous cells.[83] Cell cycle checkpoints regulate the mechanism of apoptosis or natural cell death,[84] and most tumor cells are resistant to apoptosis or natural cell death.[85] Cell cycle regulators, including KRAS, EGFR, and BRAF, are involved in several significant molecular pathways in NSCLC.[86] 31 genes from the H-VAE-Cox model and 90 genes from the XAT-VAE-Cox model were associated with the cell cycle pathway with Benjamini values $3.35e-5$ and $3.8e-28$, respectively. Another significant pathway identified was the PI3K-AKT pathway, where 74 genes from the H-VAE-Cox model and 175 genes from the XAT-VAE-Cox model were associated with this pathway, with Benjamini values $2.44e-08$ and $3.15e-42$, respectively. PI3K-AKT pathway is a transduction pathway that plays an essential role in cell growth, metabolism, proliferation, and survival.[87] Studies have shown that alterations in this pathway are likely to decrease the survival rate in NSCLC patients.[88] Previous studies have established the PI3K-AKT pathway as an interesting target for cancer therapy.[89–91]

Several studies have assessed the efficacy of proteoglycans as a significant biomarker for NSCLC.[92] The "proteoglycans in cancer" pathway is responsible for the regulation of various cellular processes such as adhesion, proliferation, differentiation, survival, and death.[93] 42 genes from the H-VAE-Cox model and 140 genes from the XAT-VAE-Cox model were associated with the proteoglycans in cancer pathway, with Benjamini values $7.16e-5$ and $7.47e-57$, respectively. Tumor-necrosis factor (TNF) signaling pathway plays an essential role in apoptosis, cellular differentiation, survival, and proliferation.[94] TNF pathway was associated with 27 genes from the H-VAE-Cox model and 64 genes from the XAT-VAE-Cox model, with Benjamini values of $1.43e-2$ and $1.636e-19$, respectively. The TNF pathway is found to be more significant in the XAT-VAE-Cox model than in the H-VAE-Cox model. Previous studies have established the TNF signaling pathway as a significant biomarker for NSCLC therapy.[95] It has been observed that the genes related to apoptosis in the TNF signaling pathway are linked with the survival of NSCLC patients.[96]

The focal adhesion pathway is linked to focal adhesion kinase (FAK). This is a cytoplasmic tyrosine kinase that is crucial for cellular signaling. Overexpression and activation of FAK have been associated with tumor progression and metastasis.[97] Studies have observed the upregulation of FAK in NSCLC patients[98] and its relationship with the metastasis of NSCLC.[99] The focal adhesion pathway was associated with 51 genes from the H-VAE-Cox model and 131 genes from the XAT-VAE-Cox model, with Benjamini values of $1.58e-8$ and $6.57e-49$, respectively. The comparison of the enriched pathways for the H-VAE-Cox and the XAT-VAE-Cox models revealed that the significant prognostic genes identified by the SHAP interpretation of the XAT-VAE-Cox model contained more NSCLC-related biological knowledge than the prognostic genes identified by the H-VAE-Cox model. Figures 7A and 7C depict the bar graphs for the count of genes involved in the top 20 pathways, where the significance of each pathway was determined using the Benjamini value (i.e., adjusted p values).

In addition to the above pathways, we thoroughly investigated the NSCLC KEGG pathway. The H-VAE-Cox and XAT-VAE-Cox models identified 24 and 51 genes associated with the NSCLC pathway, respectively, as shown in Figure 7B. The mutation of KRAS, EGFR, TRIM59, P53, cyclines, P16INK4, P14ARF, survivin, VEGF, and telomerase are considered potentially clinically useful as prognostic biomarkers and several studies have demonstrated their negative correlation with survival time.[100,101] Most oncogenes and tumor suppressor genes for the NSCLC pathway (e.g., EGFR, KRAS, P53, CDKN2A, and PIK3CA) were identified as important genes by both models for high-risk patients (i.e., patients with low survival rates).

We then biologically interpreted those selected genes using Reactome pathways (Figures 7D–7F). ReactomePA[102] was used for enrichment analysis and to identify significant biological processes via hypergeometric testing. Reactome pathways such as "diseases of signal transduction by growth factor receptors and second messengers," "signaling by interleukins," "M phase," "cell cycle checkpoints," "signaling by ROBO receptors," and "regulation of expression of SLITs and ROBOs" were identified as the most significant pathways with low

adjusted p value and high gene ratio. The gene ratio of the diseases of signal transduction by growth factor receptors and second messengers pathway was the highest for both models. This pathway is a hierarchical pathway with signaling pathways as children pathways; therefore, we further investigated the children pathways and, for both the models, cancer-causing signaling pathways were significantly enriched.

Figures 7E and 7F illustrate the top 30 significant Reactome pathways identified by the ReactomePA package for the XAT-VAE-Cox and H-VAE-Cox models, respectively. Reactome pathways such as diseases of signal transduction by growth factor receptors and second messengers, signaling by interleukins, M phase, cell cycle checkpoints, signaling by ROBO receptors, and regulation of expression of SLITs and ROBOs were identified as the most significant pathways with low adjusted p value and high gene ratio.

As the gene ratio of the diseases of signal transduction by growth factor receptors and second messengers pathway was the highest for both models, we further investigated this particular pathway using the reactome.org Website to identify significant disease-related Reactome signaling pathways. Figure 7D illustrates the significance of signaling pathways associated with the diseases of signal transduction by growth factor receptors and second messengers pathway for the H-VAE-Cox and XAT-VAE-Cox models. Signaling by EGFR in cancer is one of the significant signaling pathways, with 19 genes from the XAT-VAE-Cox model and 11 genes from the H-VAE-Cox model overlapping with the background gene list. EGFR is a tyrosine kinase (TK) receptor that is activated when it binds to the epidermal growth factor and other growth factor ligands, activating several downstream pathways such as RAS/MAPK, PI3K/Akt, and STAT, which regulate various cellular processes, including DNA synthesis and proliferation. EGFR signaling is frequently disrupted in cancer, including NSCLC.[103] Specifically, in approximately 50% of NSCLC cases, EGFR expression is found activated.[104]

Similarly, the PI3K/AKT signaling in cancer pathway is another significant pathway identified with 49 genes from the XAT-VAE-Cox model and 22 genes from the H-VAE-Cox model overlapping with the background gene list. The PI3K/Akt/mTOR signaling pathway is critical in the control of cellular development and metabolism. This pathway has been involved in both carcinogenesis and disease progression in NSCLC.[91] The “signaling by KIT in disease” signal pathway activated by onco-miRNA, miR-1260b, and mediated by YY1 regulates cell proliferation and apoptosis in NSCLC was also identified as a significant pathway, with 175 genes from the XAT-VAE-Cox model and 74 genes from the H-VAE-Cox model overlapping with the background gene list.[105] The genes selected from the XAT-VAE-Cox model were found to be more significant for cancer-related pathways compared to the genes from the H-VAE-Cox model, suggesting that the XAT-VAE-Cox model was able to learn more about cancer-related biological processes than the H-VAE-Cox model.

Gene set enrichment analysis

To explore the biological basis of high-risk and low-risk patients, we then performed a GSEA.[106] The PI estimated by H-VAE-Cox and XAT-VAE-Cox models were split into two groups, where samples having a PI value greater than the median value were categorized as high risk and samples having PI less than or equal to the median value were categorized as low risk. The samples categorized into high- and low-risk groups were used as differentiating phenotypes for GSEA. The enrichment score was calculated based on the KEGG pathway for the NSCLC pathway and pathways in cancer downloaded from the Molecular Signatures Database (MSigDB). The gene set enrichment score reflects the degree to which a gene set is overrepresented at the top or bottom of a ranked list of genes. GSEA calculates the enrichment score by walking down the ranked list of genes, increasing a running-sum statistic when a gene is in the gene set and decreasing it when it is not. The magnitude of the increment depends on the association of the gene with the phenotype (high-risk and low-risk groups). The enrichment score (ES) quantifies the association of the rank of genes with pathways, and it is validated with a false discovery rate (FDR) as corrected for multiple comparisons. The enrichment plots for the NSCLC pathway and pathways in cancer for the XAT-VAE-Cox model are reported in Figures 7G and 7H, respectively. Figures 7I and 7J report the enrichment plots for both pathways when using the H-VAE-Cox model. The XAT-VAE-Cox model ES was higher than the H-VAE-Cox model score for both pathways (i.e., NSCLC pathway and pathways in cancer), indicating that the XAT-VAE-Cox model estimation was highly associated with those pathways.

Baseline VAE-Cox model

In order to investigate the impact of sparsity on the performance of the proposed models for small sample CT-scan images, gene expression, and clinical data, a baseline variational autoencoder was designed by modifying the XAT-VAE-Cox model. Specifically, the baseline variational autoencoder was designed by removing the attention mechanism and replacing the sparse connection between the gene and pathway layers with a dense layer, as shown in Figure S2. The image modality in the encoder was constructed using pre-trained VGG-19 layers, while the gene modality was constructed using dense layers. The latent vector μ and clinical data were input to the subsequent Cox regression component. The encoder's output vector μ was concatenated with the clinical layer, followed by the Cox proportional hazard layer, while the decoder could reconstruct the images and gene expression data from a homogeneous latent representation.

The baseline-VAE-Cox model did not incorporate any pathway-related information or attention mechanism, providing a baseline performance for the survival prediction model without prior biological knowledge or feature selection. The model was trained using nested cross-fold validation, where the inner loop was used to tune the hyperparameters, while the outer loop was used to evaluate the model performance. The trained model was then evaluated on unseen datasets, namely TCGA-LUAD and TCGA-LUSC. Five independent experiments were conducted, and the model predicted the survival of NSCLC patients with a C-index of 0.59 ± 0.08, 0.58 ± 0.02, and 0.52 ± 0.05 on NSCLC-Radiogenomics, TCGA-LUAD, and TCGA-LUSC datasets, respectively (Table S2).

To further investigate the advantage of the sparsity in the proposed models on identifying the potential prognostic biomarker genes and associated pathways, we performed the SHAP interpretation for the baseline-VAE-Cox model and investigated the important genes identified by this model. Figure S3A depicts the top 40 genes identified as important by the SHAP interpretation of the Baseline-VAE-Cox model. When the top 40 genes from the SHAP summary plot were analyzed for KEGG pathways using DAVID, it was found that none of the KEGG pathways were enriched. Then the study was extended by selecting the top 15% (i.e., 1,714) genes for KEGG pathways analysis. It was found that, out of 1,714 genes, only 730 genes were included in KEGG pathways; however, only two pathways: Lysosome (Benjamini value = 0.048926) and cell cycle (Benjamini value = 0.048926) pathways were enriched.

Hence, this experiment demonstrates that the sparsity in the proposed variational autoencoder not only improves the predictive performance of the model but also helps identify prognostic biomarkers in high-risk NSCLC patients. Moreover, the XAT-VAE-Cox model was able to identify biologically relevant pathways and genes that were associated with the survival outcome of the patients, compared to the baseline-VAE-Cox model. Therefore, we conclude that the sparsity in the autoencoder, the incorporation of pathway information, and the attention mechanism are beneficial for improving the accuracy of survival analysis of cancer patients when using multimodal data.

DISCUSSION

Radiogenomics is an emerging field of research that combines radiological images and gene expression data to extract meaningful information for cancer diagnosis and prognosis, therefore supporting decision making and precision medicine.[107] However, the integration of such heterogeneous data is complex and challenging. Several data integration strategies like early integration, intermediate integration, and late integration have been attempted for multi-omics data integration for cancer diagnosis and prognosis.[108,109] Because of the heterogeneity of the data types (multi-dimensional images and high-dimensional gene expression), an early integration approach is often infeasible. Hence, an intermediate integration strategy to integrate radiological images, gene expression data, and clinical information for NSCLC survival prediction was proposed here. Furthermore, the small sample size for radiological images and gene expression posed the challenge of designing a robust and efficient prediction model from only 130 samples. As observed in the experiment conducted by Subramanian et al.[110] on the data used in this paper, developing a robust and efficient deep-learning architecture for survival prediction using a small sample size remains an open challenge. This is of high importance in the clinical context, as several studies involve a very small number of patients.

We here addressed these challenges by proposing two sparse autoencoder-based Cox architectures (H-VAE-Cox and XAT-VAE-Cox). In particular, a sparse connection was created between gene and pathway layers in both architectures, where a pathway mask based on KEGG and Reactome information was used to create the sparsity between the layers, adding further biological knowledge within the learning process and allowing more comprehensive interpretation (see sparse connection between the gene and pathway layers). We used both architectures for the intermediate integration of heterogeneous data (i.e., CT-scan images, gene expression, and clinical data) to estimate patients' PI. To ensure that the proposed models are robust (i.e., they are not overfitting or underfitting), we adopted a nested cross-validation approach to train and validate them. Specifically, the inner loops were used to tune the hyperparameters and train the model with the identified best hyperparameter, while the outer loops were used to validate the trained model.

The models trained on 130 samples were further evaluated on additional cohort datasets: TCGA-LUAD and TCGA-LUSC. To ensure the robustness of the models, the TCGA-LUAD and TCGA-LUSC datasets were never used for tuning the hyperparameters or training the models but only for the final evaluation of the proposed models. While both H-VAE-Cox and XAT-VAE-Cox models increased their accuracy when integrating imaging, gene expression, and clinical data, compared to using a single or two data modalities, the attention-based XAT-VAE-Cox model trained on small sample size was more robust in making predictions on the unseen TCGA-LUAD and TCGA-LUSC datasets (0.68 ± 0.03 and 0.65 ± 0.05, respectively) compared to the H-VAE-Cox model (0.64 ± 0.06 and 0.60 ± 0.04).

To assess the proposed architectures, we examined the predictive performance of single omics and multiple combinations of omics data. We observed that, compared to single-omics data, the integration of multi-omics data improves survival prediction. For instance, (1) the combination of image and clinical data outperforms image-only data; (2) the combination of gene expression and clinical data outperforms gene expression data; and (3) the integration of radiological images, gene expression, and clinical data significantly improves survival prediction. It was observed that the model trained using only clinical data stratified high- and low-risk group patients with a p value of 0.116 and estimated the PI with a C-index of 0.55 ± 0.10. Importantly, this suggests that only clinical information is not sufficient to precisely stratify patients into risk groups. Hence, the combination of other omics datasets along with clinical data significantly improves the survival estimation.

Having achieved more accurate survival prediction using integrated multi-omics data, it is crucial to investigate whether the models are considering biologically significant features as important features in deriving the prediction. Therefore, to interpret the proposed models and investigate significant genes and biological processes associated with high-risk-group patients, the trained models were interpreted using SHAP values. We identified important genes, clinical features, and regions in radiological images for the PI prediction (see Figure 5). We observed that specific tumor regions are considered important features for survival prediction. Similarly, both models identified disease recurrence as the most important clinical feature for high-risk patients.

To further biologically interpret the results and assess the biological knowledge learned during the training phase, the top 40 important genes identified by the SHAP interpretation of both models were investigated for KEGG pathways and functional

CellPress
OPEN ACCESS

GO (Figure 6). The study was then expanded by selecting the top 15% (i.e., 1,714) genes sorted by SHAP value for the KEGG and Reactome pathway analysis. The results showed that the significant prognostic genes are highly associated with cancer-related KEGG pathways. Notably, XAT-VAE-Cox selected a larger number of genes associated with top-ranked cancer-related KEGG pathways compared to H-VAE-Cox. The significant pathways determined by the Benjamini value were found to be more related to NSCLC-causing pathways for XAT-VAE-Cox compared to H-VAE-Cox (Figures 6 and 7A–7C). The NSCLC KEGG pathway and pathways in cancer were enriched when performing GSEA with positive ESs (Figures 7G–7J).

Similarly, the Reactome pathway analysis (Figures 7D–7F) performed on the selected genes, identified cancer-related pathways with high gene ratios, including the diseases of signal transduction by growth factor receptors and second messengers pathway, which had the highest gene ratio and the lowest p value for both models. The cancer-related signaling pathways under diseases of signal transduction by growth factor receptors and second messengers (e.g., signaling by EGFR in cancer, signaling by FGFR in cancer, PI3K/AKT signaling in cancer, signaling by ALK in cancer, signaling by NOTCH1 in cancer, oncogenic MAPK signaling, etc.) were identified as significant pathways from the model-selected genes (Figure 7D). When compared to H-VAE-Cox, the significant prognostic genes identified by XAT-VAE-Cox had a larger number of overlaps with background gene lists for these signaling pathways. Thus, the proposed models were able to learn cancer-causing biological knowledge, while the top-ranked genes identified by XAT-VAE-Cox were more relevant in cancer-related biological processes, as shown by the biological interpretation analysis.

In summary, our results suggest that the integration of radiological images with gene expression data and clinical data in a deep neural network framework can improve survival prediction in the presence of a small dataset. The integration of gene expression data and clinical data improved the predictive performance compared to using only gene expression data. Similarly, the integration of images and clinical data performed better compared to only images and, most importantly, the integration of gene expression, images, and clinical data outperformed all previous models, with the highest C-index and lowest p value. Furthermore, when compared to DeepSurv and DCM models, both our proposed models achieved a more accurate survival prediction using low-sample-size images, gene expression, and clinical data. We note that, while both the H-VAE-Cox and XAT-VAE-Cox models perform well in terms of survival prediction, XAT-VAE-Cox can learn more biological knowledge and identify significant cancer-related genes and biological pathways. We envision that the integration of radiomic features with multi-omics (transcriptomics, proteomics, epigenomics, and metabolic) data will further improve the performance of our models and enhance our understanding of significant genes and biological processes associated with cancer.

Limitations of the study

While both proposed models accurately stratify patients into risk groups when trained on a dataset of only 130 patients, the best model to be adopted depends on the modeling priorities. H-VAE-Cox, being a modular model, requires fewer computational resources, and can readily incorporate additional modalities, but it has limited interpretability. In particular, H-VAE-Cox is not able to precisely highlight the tumor regions in the high-risk categorized CT-scan images. Conversely, XAT-VAE-Cox, with a built-in attention mechanism, is better able to learn from cross-modality information, making the model more interpretable. However, as all the data modalities are integrated within a single framework, it requires higher computational resources. In cases where high computational resources are not available and the interpretability of the model is less important, H-VAE-Cox could be adopted.

STAR★METHODS

Detailed methods are provided in the online version of this paper and include the following:

- KEY RESOURCES TABLE
- RESOURCE AVAILABILITY
  - Lead contact
  - Materials availability
  - Data and code availability
- METHOD DETAILS
  - Data collection and preprocessing
  - Data for external validation
  - H-VAE-Cox: Hierarchical Variational Autoencoder-based Cox model
  - Encoder
  - Decoder
  - Cox regression component
  - XAT-VAE-Cox: Cross-attention-based sparse Variational Autoencoder Cox model
  - Sparse connection between the gene and pathway layers
  - Attention mechanism
  - Experimental design and model evaluation
  - Model interpretation
  - Model tuning and hyperparameter optimisation
- QUANTIFICATION AND STATISTICAL ANALYSIS

SUPPLEMENTAL INFORMATION

Supplemental information can be found online at https://doi.org/10.1016/j.crmeth.2024.100817.

ACKNOWLEDGMENTS

C.A. acknowledges a Network Development Award and Turing Network Funding from The Alan Turing Institute (grants TNDC2-100022 and D-ELA-013). A.O. acknowledges grants from Earlier.org, the National Biofilms Innovation Centre (NBIC), and EPSRC (grant EP/Y001613/1).

AUTHOR CONTRIBUTIONS

S.V., methodology, software, investigation, and writing – original draft; G.M. and N.E., methodology, validation, and resources; T.L. and A.G., validation; A.O., conceptualization, methodology, supervision, writing – review & editing, and project administration; C.A., conceptualization, methodology, writing – review & editing, supervision, project administration, and funding acquisition.

DECLARATION OF INTERESTS

The authors declare no competing interests.

Received: August 29, 2023
Revised: April 18, 2024
Accepted: June 17, 2024
Published: July 8, 2024

REFERENCES

1. Thandra, K.C., Barsouk, A., Saginala, K., Aluru, J.S., and Barsouk, A. (2021). Epidemiology of lung cancer. Contemp. Oncol. *25*, 45–52.
2. Chi, S.A., Yu, H., Choi, Y.-L., Park, S., Sun, J.-M., Lee, S.-H., Ahn, J.S., Ahn, M.-J., Choi, D.-H., Kim, K., et al. (2023). Trends in survival rates of non–small cell lung cancer with use of molecular testing and targeted therapy in korea, 2010-2020. JAMA Netw. Open *6*, e232002.
3. Min, H.-Y., and Lee, H.-Y. (2021). Mechanisms of resistance to chemotherapy in non-small cell lung cancer. Arch Pharm. Res. (Seoul) *44*, 146–164.
4. Bagcchi, S. (2017). Lung cancer survival only increases by a small amount despite recent treatment advances. Lancet Respir. Med. *5*, 169.
5. Patel, A.J., Tan, T.-M., Richter, A.G., Naidu, B., Blackburn, J.M., and Middleton, G.W. (2022). A highly predictive autoantibody-based biomarker panel for prognosis in early-stage nsclc with potential therapeutic implications. Br. J. Cancer *126*, 238–246.
6. Lou, F., Huang, J., Sima, C.S., Dycoco, J., Rusch, V., and Bach, P.B. (2013). Patterns of recurrence and second primary lung cancer in early-stage lung cancer survivors followed with routine computed tomography surveillance. J. Thorac. Cardiovasc. Surg. *145*, 75–82.
7. Angione, C. (2019). Human systems biology and metabolic modelling: A review—from disease metabolism to precision medicine. BioMed Res. Int. *2019*, 8304260.
8. Lo Gullo, R., Daimiel, I., Morris, E.A., and Pinker, K. (2020). Combining molecular and imaging metrics in cancer: Radiogenomics. Insights Imaging *11*, 1–17.
9. Peters, S., Dziadziuszko, R., Morabito, A., Felip, E., Gadgeel, S.M., Cheema, P., Cobo, M., Andric, Z., Barrios, C.H., Yamaguchi, M., et al. (2022). Atezolizumab versus chemotherapy in advanced or metastatic nsclc with high blood-based tumor mutational burden: Primary analysis of bfast cohort c randomized phase 3 trial. Nat. Med. *28*, 1831–1839.
10. Tomaszewski, J.J., Uzzo, R.G., and Smaldone, M.C. (2014). Heterogeneity and renal mass biopsy: A review of its role and reliability. Cancer Biol. Med. *11*, 162–172.
11. Liu, L., Sun, H., and Li, F. (2023). A lie group kernel learning method for medical image classification. Pattern Recogn. *142*, 109735.
12. Aerts, H.J.W.L., Velazquez, E.R., Leijenaar, R.T.H., Parmar, C., Grossmann, P., Carvalho, S., Bussink, J., Monshouwer, R., Haibe-Kains, B., Rietveld, D., et al. (2014). Decoding tumour phenotype by noninvasive imaging using a quantitative radiomics approach. Nat. Commun. *5*, 4006–4009.
13. Liu, Z., Wu, F., Wang, Y., Yang, M., and Pan, X. (2023). Fedcl: Federated contrastive learning for multi-center medical image classification. Pattern Recogn. *143*, 109739.
14. Baek, S., He, Y., Allen, B.G., Buatti, J.M., Smith, B.J., Tong, L., Sun, Z., Wu, J., Diehn, M., Loo, B.W., et al. (2019). Deep segmentation networks predict survival of non-small cell lung cancer. Sci. Rep. *9*, 17286.
15. Conway, J., Pouryahya, M., Gindin, Y., Pan, D.Z., Carrasco-Zevallos, O.M., Mountain, V., Subramanian, G.M., Montalto, M.C., Resnick, M., Beck, A.H., et al. (2023). Integration of deep learning-based histopathology and transcriptomics reveals key genes associated with fibrogenesis in patients with advanced nash. Cell Rep. Med. *4*, 101016.
16. Steyaert, S., Pizurica, M., Nagaraj, D., Khandelwal, P., Hernandez-Boussard, T., Gentles, A.J., and Gevaert, O. (2023). Multimodal data fusion for cancer biomarker discovery with deep learning. Nat. Mach. Intell. *5*, 351–362.
17. Hong, R., Liu, W., DeLair, D., Razavian, N., and Fenyö, D. (2021). Predicting endometrial cancer subtypes and molecular features from histopathology images using multi-resolution deep learning models. Cell Rep. Med. *2*, 100400.
18. Magazzù, G., Zampieri, G., and Angione, C. (2022). Clinical stratification improves the diagnostic accuracy of small omics datasets within machine learning and genome-scale metabolic modelling methods. Comput. Biol. Med. *151*, 106244.
19. Kang, M., Ko, E., and Mersha, T.B. (2022). A roadmap for multi-omics data integration using deep learning. Briefings Bioinf. *23*, bbab454.
20. Smedley, N.F., El-Saden, S., and Hsu, W. (2020). Discovering and interpreting transcriptomic drivers of imaging traits using neural networks. Bioinformatics *36*, 3537–3548.
21. Verma, S., Razzaque, M.A., Sangtongdee, U., Arpnikanondt, C., Tassaneetrithep, B., and Hossain, A. (2021). Digital diagnosis of hand, foot, and mouth disease using hybrid deep neural networks. IEEE Access *9*, 143481–143494.
22. Jiang, S., Suriawinata, A.A., and Hassanpour, S. (2023). Mhattnsurv: Multi-head attention for survival prediction using whole-slide pathology images. Comput. Biol. Med. *158*, 106883.
23. Chicco, D., Cumbo, F., and Angione, C. (2023). Ten quick tips for avoiding pitfalls in multi-omics data integration analyses. PLoS Comput. Biol. *19*, e1011224.
24. Ellen, J.G., Jacob, E., Nikolaou, N., and Markuzon, N. (2023). Autoencoder-based multimodal prediction of non-small cell lung cancer survival. Sci. Rep. *13*, 15761.
25. Liu, H., Shi, Y., Li, A., and Wang, M. (2024). Multi-modal fusion network with intra-and inter-modality attention for prognosis prediction in breast cancer. Comput. Biol. Med. *168*, 107796.
26. Zhang, Y., Sun, B., Yu, Y., Lu, J., Lou, Y., Qian, F., Chen, T., Zhang, L., Yang, J., Zhong, H., et al. (2024). Multimodal fusion of liquid biopsy and ct enhances differential diagnosis of early-stage lung adenocarcinoma. npj Precis. Oncol. *8*, 50.
27. Doan, L.M.T., Angione, C., and Occhipinti, A. (2022). Machine learning methods for survival analysis with clinical and transcriptomics data of breast cancer. In Computational biology and machine learning for metabolic engineering and synthetic biology (Springer), pp. 325–393.
28. Wang, P., Li, Y., and Reddy, C.K. (2019). Machine learning for survival analysis: A survey. ACM Comput. Surv. *51*, 1–36.
29. Occhipinti, A., Verma, S., and Angione, C. (2024). Mechanism-aware and multimodal ai: Beyond model-agnostic interpretation. Trends Cell Biol. *34*, 85–89.
30. Park, J., Lee, J.W., and Park, M. (2023). Comparison of cancer subtype identification methods combined with feature selection methods in omics data analysis. BioData Min. *16*, 18–24.
31. Bhadra, T., Mallik, S., Hasan, N., and Zhao, Z. (2022). Comparison of five supervised feature selection algorithms leading to top features and gene signatures from multi-omics data in cancer. BMC Bioinf. *23*, 153.
32. Sun, D., Wang, M., and Li, A. (2018). A multimodal deep neural network for human breast cancer prognosis prediction by integrating multi-dimensional data. IEEE ACM Trans. Comput. Biol. Bioinf *16*, 841–850.
33. Sun, T., Wei, Y., Chen, W., and Ding, Y. (2020). Genome-wide association study-based deep learning for survival prediction. Stat. Med. *39*, 4605–4620.
34. Zampieri, G., Vijayakumar, S., Yaneske, E., and Angione, C. (2019). Machine and deep learning meet genome-scale metabolic modeling. PLoS Comput. Biol. *15*, e1007084.
35. Katzman, J.L., Shaham, U., Cloninger, A., Bates, J., Jiang, T., and Kluger, Y. (2018). Deepsurv: Personalized treatment recommender system using a cox proportional hazards deep neural network. BMC Med. Res. Methodol. *18*, 24.

CellPress
OPEN ACCESS

36. Ching, T., Zhu, X., and Garmire, L.X. (2018). Cox-nnet: An artificial neural network method for prognosis prediction of high-throughput omics data. PLoS Comput. Biol. *14*, e1006076.
37. Magazzù, G., Zampieri, G., and Angione, C. (2021). Multimodal regularized linear models with flux balance analysis for mechanistic integration of omics data. Bioinformatics *37*, 3546–3552.
38. Nagpal, C., Yadlowsky, S., Rostamzadeh, N., and Heller, K. (2021). Deep cox mixtures for survival regression. In Machine Learning for Healthcare Conference, pp. 674–708.
39. Hao, J., Kosaraju, S.C., Tsaku, N.Z., Song, D.H., and Kang, M. (2019). Page-net: Interpretable and integrative deep learning for survival analysis using histopathological images and genomic data. In Pacific Symposium on Biocomputing 2020, pp. 355–366.
40. Hsu, T.-C., and Lin, C. (2023). Learning from small medical data—robust semi-supervised cancer prognosis classifier with bayesian variational autoencoder. Bioinform. Adv. *3*, vbac100.
41. Arya, N., Saha, S., Mathur, A., and Saha, S. (2023). Improving the robustness and stability of a machine learning model for breast cancer prognosis through the use of multi-modal classifiers. Sci. Rep. *13*, 4079.
42. Yan, T., Yan, Z., Liu, L., Zhang, X., Chen, G., Xu, F., Li, Y., Zhang, L., Peng, M., Wang, L., et al. (2022). Survival prediction for patients with glioblastoma multiforme using a cox proportional hazards denoising autoencoder network. Front. Comput. Neurosci. *16*, 916511.
43. Wu, X., and Fang, Q. (2022). Stacked Autoencoder Based Multi-Omics Data Integration for Cancer Survival Prediction. Preprint at arXiv. https://doi.org/10.48550/arXiv.2207.04878.
44. Torkey, H., Atlam, M., El-Fishawy, N., and Salem, H. (2021). A novel deep autoencoder based survival analysis approach for microarray dataset. PeerJ. Comput. Sci. *7*, e492.
45. Loureiro, H., Becker, T., Bauer-Mehren, A., Ahmidi, N., and Weberpals, J. (2021). Artificial intelligence for prognostic scores in oncology: A benchmarking study. Front. Artif. Intell. *4*, 625573.
46. Hira, M.T., Razzaque, M.A., Angione, C., Scrivens, J., Sawan, S., and Sarker, M. (2021). Integrated multi-omics analysis of ovarian cancer using variational autoencoders. Sci. Rep. *11*, 6265.
47. Bakr, S., Gevaert, O., Echegaray, S., Ayers, K., Zhou, M., Shafiq, M., Zheng, H., Benson, J.A., Zhang, W., Leung, A.N.C., et al. (2018). A radiogenomic dataset of non-small cell lung cancer. Sci. Data *5*, 180202–180209.
48. Albertina, B., Watson, M., Holback, C., Jarosz, R., Kirk, S., Lee, Y., and Lemmerman, J. (2016). Radiology Data from the Cancer Genome Atlas Lung Adenocarcinoma, *10* (The Cancer Imaging Archive), p. K9, [tcga-luad] collection.
49. Kirk, S., Lee, Y., Kumar, P., Filippini, J., Albertina, B., Watson, M., and Lemmerman, J. (2016). Radiology Data from the Cancer Genome Atlas Lung Squamous Cell Carcinoma [tcga-Lusc] Collection (The Cancer Imaging Archive).
50. Lundberg, S.M., and Lee, S.-I. (2017). A unified approach to interpreting model predictions. In Proceedings of the 31st International Conference on Neural Information Processing Systems, pp. 4768–4777.
51. Ghosal, S., and Shah, P. (2021). A deep-learning toolkit for visualization and interpretation of segmented medical images. Cell Rep. Methods *1*, 100107.
52. Steck, H., Krishnapuram, B., Dehing-Oberije, C., Lambin, P., and Raykar, V.C. (2008). On ranking in survival analysis: Bounds on the concordance index. Adv. Neural Inf. Process. Syst., 1209–1216.
53. Milletari, F., Navab, N., and Ahmadi, S.-A. (2016). V-net: Fully convolutional neural networks for volumetric medical image segmentation. In 2016 Fourth International Conference on 3D Vision (3DV), pp. 565–571.
54. Bradski, G., and Kaehler, A. (2008). Learning Opencv: Computer Vision with the Opencv Library (O'Reilly Media, Inc.).
55. Therneau, T.M., and Lumley, T. (2015). Package 'survival'. R Top Doc *128*, 28–33.
56. Kvamme, H., Borgan, Ø., and Scheel, I. (2019). Time-to-event prediction with neural networks and cox regression. J. Mach. Learn. Res. *20*, 1–30.
57. Kalainayakan, S.P., FitzGerald, K.E., Konduri, P.C., Vidal, C., and Zhang, L. (2018). Essential roles of mitochondrial and heme function in lung cancer bioenergetics and tumorigenesis. Cell Biosci. *8*, 56.
58. He, Y., Sun, M.M., Zhang, G.G., Yang, J., Chen, K.S., Xu, W.W., and Li, B. (2021). Targeting pi3k/akt signal transduction for cancer therapy. Signal Transduct. Targeted Ther. *6*, 425.
59. Zhao, Y., Guo, S., Deng, J., Shen, J., Du, F., Wu, X., Chen, Y., Li, M., Chen, M., Li, X., et al. (2022). Vegf/vegfr-targeted therapy and immunotherapy in non-small cell lung cancer: Targeting the tumor microenvironment. Int. J. Biol. Sci. *18*, 3845–3858.
60. Cao, W., Tang, Q., Zeng, J., Jin, X., Zu, L., and Xu, S. (2023). A review of biomarkers and their clinical impact in resected early-stage non-small-cell lung cancer. Cancers *15*, 4561.
61. Zhang, R., Chen, C., Dong, X., Shen, S., Lai, L., He, J., You, D., Lin, L., Zhu, Y., Huang, H., et al. (2020). Independent validation of early-stage non-small cell lung cancer prognostic scores incorporating epigenetic and transcriptional biomarkers with gene-gene interactions and main effects. Chest *158*, 808–819.
62. Priest, K., Le, A., Gebregzabheir, A., Nijmeh, H., Reis, G.B., Mandell, M., Davies, K.D., Lawrence, C., O'Donnell, E., Doebele, R.C., et al. (2023). Evolution of acquired resistance in a ros1+ kras g12c+ nsclc through the mapk pathway. npj Precis. Oncol. *7*, 9.
63. Liu, K., Wang, L., Lou, Z., Guo, L., Xu, Y., Qi, H., Fang, Z., Mei, L., Chen, X., Zhang, X., et al. (2023). E2f8 exerts cancer-promoting effects by transcriptionally activating rrm2 and e2f8 knockdown synergizes with wee1 inhibition in suppressing lung adenocarcinoma. Biochem. Pharmacol. *218*, 115854.
64. Wu, X., Song, W., Cheng, C., Liu, Z., Li, X., Cui, Y., Gao, Y., and Li, D. (2023). Small molecular inhibitors for kras-mutant cancers. Front. Immunol. *14*.
65. Sun, H., Zhang, H., Yan, Y., Li, Y., Che, G., Zhou, C., Nicot, C., and Ma, H. (2022). Ncapg promotes the oncogenesis and progression of non-small cell lung cancer cells through upregulating lgals1 expression. Mol. Cancer *21*, 55.
66. Yan, L.-D., Yang, L., Li, N., Wang, M., Zhang, Y.-H., Zhou, W., Yu, Z.-Q., Peng, X.-C., and Cai, J. (2022). Prognostic role of multiple abnormal genes in non-small-cell lung cancer. World J. Clin. Cases *10*, 7772–7784.
67. Wu, T., Hu, E., Xu, S., Chen, M., Guo, P., Dai, Z., Feng, T., Zhou, L., Tang, W., Zhan, L., et al. (2021). Clusterprofiler 4.0: A universal enrichment tool for interpreting omics data. Innovation *2*, 100141.
68. Raimondi, V., Ciccarese, F., and Ciminale, V. (2020). Oncogenic pathways and the electron transport chain: A dangeros liaison. Br. J. Cancer *122*, 168–181.
69. Popov, L.-D. (2023). Mitochondria as intracellular signalling organelles. an update. Cell. Signal. *109*, 110794.
70. Liu, S.-Y.M., Zheng, M.-M., Pan, Y., Liu, S.-Y., Li, Y., and Wu, Y.-L. (2023). Emerging evidence and treatment paradigm of non-small cell lung cancer. J. Hematol. Oncol. *16*, 40.
71. Xiao, Y., Liu, P., Wei, J., Zhang, X., Guo, J., and Lin, Y. (2023). Recent progress in targeted therapy for non-small cell lung cancer. Front. Pharmacol. *14*, 399.
72. Wee, P., and Wang, Z. (2017). Epidermal growth factor receptor cell proliferation signaling pathways. Cancers *9*, 52.
73. Roskoski, R., Jr. (2019). Small molecule inhibitors targeting the egfr/erbb family of protein-tyrosine kinases in human cancers. Pharmacol. Res. *139*, 395–411.
74. Zhang, J., Xiang, Q., Wu, M., Lao, Y.-Z., Xian, Y.-F., Xu, H.-X., and Lin, Z.-X. (2023). Autophagy regulators in cancer. Int. J. Mol. Sci. *24*, 10944.
75. Yewale, C., Baradia, D., Vhora, I., Patil, S., and Misra, A. (2013). Epidermal growth factor receptor targeting in cancer: A review of trends and strategies. Biomaterials *34*, 8690–8707.

76. Muste Sadurni, M., and Saponaro, M. (2023). Deregulations of rna pol ii subunits in cancer. Applied Biosciences *2*, 459–476.

77. Khan, I., and Steeg, P.S. (2021). Endocytosis: A pivotal pathway for regulating metastasis. Br. J. Cancer *124*, 66–75.

78. Wang, J., Chen, S., Wang, H., Cao, J., Fan, X., Man, J., Li, Q., and Yang, L. (2022). Integrated molecular analyses of an interferon- based subtype with regard to outcome, immune characteristics, and immunotherapy in bladder cancer and experimental verification. Heliyon *8*, e12102.

79. Luo, Y., Li, J., Yu, P., Sun, J., Hu, Y., Meng, X., and Xiang, L. (2022). Targeting lncrnas in programmed cell death as a therapeutic strategy for non-small cell lung cancer. Cell Death Dis. *8*, 159.

80. Najafi, M., Ahmadi, A., and Mortezaee, K. (2019). Extracellular-signal-regulated kinase/mitogen-activated protein kinase signaling as a target for cancer therapy: An updated review. Cell Biol. Int. *43*, 1206–1222.

81. Shi, Z., Zhou, H., Pan, B., Lu, L., Wei, Z., Shi, L., Yao, X., Kang, Y., and Feng, S. (2017). Exploring the key genes and pathways of osteosarcoma with pulmonary metastasis using a gene expression microarray. Mol. Med. Rep. *16*, 7423–7431.

82. Guo, Z., Peng, G., Li, E., Xi, S., Zhang, Y., Li, Y., Lin, X., Li, G., Wu, Q., and He, J. (2017). Map kinase-interacting serine/threonine kinase 2 promotes proliferation, metastasis, and predicts poor prognosis in non-small cell lung cancer. Sci. Rep. *7*, 10612.

83. Hanahan, D., and Weinberg, R.A. (2000). The hallmarks of cancer. cell *100*, 57–70.

84. Baldi, A., De Luca, A., Esposito, V., Campioni, M., Spugnini, E.P., and Citro, G. (2011). Tumor suppressors and cell-cycle proteins in lung cancer. Pathol. Res. Int. *2011*, 605042.

85. Fennell, D.A. (2005). Caspase regulation in non–small cell lung cancer and its potential for therapeutic exploitation. Clin. Cancer Res. *11*, 2097–2105.

86. Eymin, B., and Gazzeri, S. (2010). Role of cell cycle regulators in lung carcinogenesis. Cell Adhes. Migrat. *4*, 114–123.

87. Hemmings, B.A., and Restuccia, D.F. (2012). Pi3k-pkb/akt pathway. Cold Spring Harbor Perspect. Biol. *4*, a011189.

88. Pérez-Ramírez, C., Cañadas-Garre, M., Molina, M.Á., Faus-Dáder, M.J., and Calleja-Hernández, M.Á. (2015). Pten and pi3k/akt in non-small-cell lung cancer. Pharmacogenomics *16*, 1843–1862.

89. Liang, J., and Slingerland, J.M. (2003). Multiple roles of the pi3k/pkb (akt) pathway in cell cycle progression. Cell Cycle *2*, 339–345.

90. Fumarola, C., Bonelli, M.A., Petronini, P.G., and Alfieri, R.R. (2014). Targeting pi3k/akt/mtor pathway in non small cell lung cancer. Biochem. Pharmacol. *90*, 197–207.

91. Tan, A.C. (2020). Targeting the pi3k/akt/mtor pathway in non-small cell lung cancer (nsclc). Thorac. Cancer *11*, 511–518.

92. Rangel, M.P., de Sá, V.K., Prieto, T., Martins, J.R.M., Olivieri, E.R., Carraro, D., Takagaki, T., and Capelozzi, V.L. (2018). Biomolecular analysis of matrix proteoglycans as biomarkers in non small cell lung cancer. Glycoconj. J. *35*, 233–242.

93. Park, P., Hayashida, K., Aquino, R., and Jinno, A. (2016). Proteoglycans. In Encyclopedia of cell biology, R.A. Bradshaw and P.D. Stahl, eds. (Academic Press), pp. 271–278. https://doi.org/10.1016/B978-0-12-394447-4.10032-X.

94. Aggarwal, B.B. (2003). Signalling pathways of the tnf superfamily: A double-edged sword. Nat. Rev. Immunol. *3*, 745–756.

95. Wang, S., Yan, Y., Cheng, Z., Hu, Y., and Liu, T. (2018). Sotetsuflavone suppresses invasion and metastasis in non-small-cell lung cancer a549 cells by reversing emt via the tnf-/nf-b and pi3k/akt signaling pathway. Cell death discovery *4*, 26.

96. Guo, Y., Feng, Y., Liu, H., Luo, S., Clarke, J.W., Moorman, P.G., Su, L., Shen, S., Christiani, D.C., and Wei, Q. (2019). Potentially functional genetic variants in the tnf/tnfr signaling pathway genes predict survival of patients with non-small cell lung cancer in the plco cancer screening trial. Mol. Carcinog. *58*, 1094–1104.

97. Zhao, J., and Guan, J.-L. (2009). Signal transduction by focal adhesion kinase in cancer. Cancer Metastasis Rev. *28*, 35–49.

98. Carelli, S., Zadra, G., Vaira, V., Falleni, M., Bottiglieri, L., Nosotti, M., Di Giulio, A.M., Gorio, A., and Bosari, S. (2006). Up-regulation of focal adhesion kinase in non-small cell lung cancer. Lung cancer *53*, 263–271.

99. Ji, H.-F., Pang, D., Fu, S.-B., Jin, Y., Yao, L., Qi, J.-P., and Bai, J. (2013). Overexpression of focal adhesion kinase correlates with increased lymph node metastasis and poor prognosis in non-small-cell lung cancer. J. Cancer Res. Clin. Oncol. *139*, 429–435.

100. Huncharek, M., Muscat, J., and Geschwind, J.-F. (1999). K-ras oncogene mutation as a prognostic marker in non-small cell lung cancer: A combined analysis of 881 cases. Carcinogenesis *20*, 1507–1510.

101. Odintsov, I., and Sholl, L.M. (2024). Prognostic and predictive biomarkers in non-small cell lung carcinoma. Pathology *56*, 192–204.

102. Yu, G., and He, Q.-Y. (2016). Reactomepa: An r/bioconductor package for reactome pathway analysis and visualization. Mol. Biosyst. *12*, 477–479.

103. Russo, A., Franchina, T., Ricciardi, G.R.R., Picone, A., Ferraro, G., Zanghì, M., Toscano, G., Giordano, A., and Adamo, V. (2015). A decade of egfr inhibition in egfr-mutated non small cell lung cancer (nsclc): Old successes and future perspectives. Oncotarget *6*, 26814–26825.

104. Amann, J., Kalyankrishna, S., Massion, P.P., Ohm, J.E., Girard, L., Shigematsu, H., Peyton, M., Juroske, D., Huang, Y., Stuart Salmon, J., et al. (2005). Aberrant epidermal growth factor receptor signaling and enhanced sensitivity to egfr inhibitors in lung cancer. Cancer Res. *65*, 226–235.

105. Xia, Y., Wei, K., Yang, F.-M., Hu, L.-Q., Pan, C.-F., Pan, X.-L., Wu, W.-B., Wang, J., Wen, W., He, Z.-C., et al. (2019). Mir-1260b, mediated by yy1, activates kit signaling by targeting socs6 to regulate cell proliferation and apoptosis in nsclc. Cell Death Dis. *10*, 112–114.

106. Shi, J., and Walker, M. (2007). Gene set enrichment analysis (gsea) for interpreting gene expression profiles. Curr. Bioinf. *2*, 133–137.

107. Liu, Z., Duan, T., Zhang, Y., Weng, S., Xu, H., Ren, Y., Zhang, Z., and Han, X. (2023). Radiogenomics: A key component of precision cancer medicine. Br. J. Cancer *129*, 741–753.

108. Stahlschmidt, S.R., Ulfenborg, B., and Synnergren, J. (2022). Multimodal deep learning for biomedical data fusion: A review. Briefings Bioinf. *23*, bbab569.

109. Adossa, N., Khan, S., Rytkönen, K.T., and Elo, L.L. (2021). Computational strategies for single-cell multi-omics integration. Comput. Struct. Biotechnol. J. *19*, 2588–2596.

110. Subramanian, V., Do, M.N., and Syeda-Mahmood, T. (2020). Multimodal fusion of imaging and genomics for lung cancer recurrence prediction. 2020 IEEE 17th International Symposium on Biomedical Imaging (ISBI), pp. 804–808.

111. Bakr, S., Gevaert, O., Echegaray, S., Ayers, K., Zhou, M., Shafiq, M., Zheng, H., Zhang, W., Leung, A., Kadoch, M., et al. (2017). Data for Nsclc Radiogenomics Collection. The Cancer Imaging Archive.

112. Huang, D.W., Sherman, B.T., and Lempicki, R.A. (2009). Systematic and integrative analysis of large gene lists using david bioinformatics resources. Nat. Protoc. *4*, 44–57. https://doi.org/10.1038/nprot.2008.211.

113. Huang, D.W., Sherman, B.T., and Lempicki, R.A. (2009). Bioinformatics enrichment tools: Paths toward the comprehensive functional analysis of large gene lists. Nucleic Acids Res. *37*, 1–13.

114. Tracy, S., Yuan, G.-C., and Dries, R. (2019). Rescue: Imputing dropout events in single-cell rna-sequencing data. BMC Bioinf. *20*, 388.

115. Li, W.V., and Li, J.J. (2018). An accurate and robust imputation method scimpute for single-cell rna-seq data. Nat. Commun. *9*, 997–999.

116. Ronneberger, O., Fischer, P., and Brox, T. (2015). U-net: Convolutional networks for biomedical image segmentation. International Conference on Medical Image Computing and Computer-Assisted Intervention, pp. 234–241.

CellPress
OPEN ACCESS

117. Simonyan, K., and Zisserman, A. (2015). Very deep convolutional networks for large-scale image recognition. 3rd International Conference on Learning Representations (ICLR 2015). Computational and Biological Learning Society, 1–14.

118. Culley, C., Vijayakumar, S., Zampieri, G., and Angione, C. (2020). A mechanism-aware and multiomic machine-learning pipeline characterizes yeast cell growth. Proc. Natl. Acad. Sci. USA *117*, 18869–18879.

119. Higgins, I., Matthey, L., Pal, A., Burgess, C., Glorot, X., Botvinick, M., Mohamed, S., and Lerchner, A. (2017). Beta-vae: Learning basic visual concepts with a constrained variational framework. International Conference on Learning Representations (ICLR).

120. Fan, Z., Jiang, Z., Liang, H., and Han, C. (2023). Pancancer survival prediction using a deep learning architecture with multimodal representation and integration. Bioinform. Adv. *3*, vbad006.

121. Hao, L., Kim, J., Kwon, S., and Ha, I.D. (2021). Deep learning-based survival analysis for high-dimensional survival data. Mathematics *9*, 1244.

122. Vaswani, A., Shazeer, N., Parmar, N., Uszkoreit, J., Jones, L., Gomez, A.N., Kaiser, Ł., and Polosukhin, I. (2017). Attention is all you need. Adv. Neural Inf. Process. Syst. *30*.

123. Maleki, F., Muthukrishnan, N., Ovens, K., Reinhold, C., and Forghani, R. (2020). Machine learning algorithm validation: From essentials to advanced applications and implications for regulatory certification and deployment. Neuroimaging Clinics *30*, 433–445.

124. Parcalabescu, L., and Frank, A. (2023). Mm-shap: A performance-agnostic metric for measuring multimodal contributions in vision and language models & tasks. In Proceedings of the 61st Annual Meeting of the Association for Computational Linguistics (Volume 1: Long Papers), pp. 4032–4059.

STAR★METHODS

KEY RESOURCES TABLE

REAGENT or RESOURCE	SOURCE	IDENTIFIER
Deposited data		
NSCLC radiogenomics	TCIA Bakr et al.[111]	GEO: GSE103584; https://www.cancerimagingarchive.net/collection/nsclc-radiogenomics
TCGA-LUAD	TCIA, TCGA Albertina et al.[48].	https://www.cancerimagingarchive.net/collection/tcga-luad/
TCGA-LUSC	TCIA, TCGA Kirk et al.[49]	https://www.cancerimagingarchive.net/collection/tcga-lusc/
KEGG and Reactome pathways	Huang et al.[112,113]	https://david.ncifcrf.gov/tools.jsp
Preprocessed CT-scan images	This paper	https://figshare.com/articles/dataset/Preprocessed_CT-scan_images_ROI_extracted_from_CT_Scan_images_/26037169 figshare: https://doi.org/10.6084/m9.figshare.26037169
Software and algorithms		
clusterprofiler R package	Tracy et al.[114]	https://www.bioconductor.org/packages/release/bioc/html/clusterProfiler.html
ReactomePA	Yu et al.[102]	https://bioconductor.org/packages/release/bioc/html/ReactomePA.html
Source code	This paper	zenodo: https://doi.org/10.5281/zenodo.11650343

RESOURCE AVAILABILITY

Lead contact

Further information and requests for resources should be directed to and will be fulfilled by the lead contact, Claudio Angione (C.Angione@tees.ac.uk).

Materials availability

This study did not generate unique reagents.

Data and code availability

- This paper analyzes existing, publicly available data. The accession numbers for the datasets are listed in the key resources table.
- Source codes are available: https://github.com/Angione-Lab/NSCLC-Survival-Prediction-models. All original code has been deposited at Zenodo and is publicly available as of the date of publication. DOIs are listed in the key resources table.
- Resource Availability: Any additional information required to reanalyze the data reported in this work paper is available from the lead contact upon request

METHOD DETAILS

Two sparse variational autoencoder-based architectures were developed: the Hierarchical Variational Autoencoder Cox model (H-VAE-Cox) and the cross-attention-based sparse Variational Autoencoder Cox model (XAT-VAE-Cox). Within these, radiological images (CT scan images) were integrated with RNA-seq data, biological pathways, and clinical data for survival prediction.

Data collection and preprocessing

For our experiment, we used NSCLC radiogenomics (i.e., a combination of radiological images and transcriptomics data) and clinical data. Specifically, CT scan images and clinical information for 211 patients were obtained from TCIA,[111] while the corresponding RNA-seq data available for 130 patients was downloaded from GEO (GSE103584).[111] The clinical data contains 38 features including gender, smoking status, EGFR mutation status, KRAS mutation status, ALK translocation status, survival status, and time to death. The RNA-seq data contains log normalised expression values of 22126 genes for 130 patients, namely 96 male patients with an average age of 69 years and 34 female patients with an average age of 64 years. Additionally, we extracted biological pathway information from DAVID,[112,113] where we focused on the KEGG and Reactome pathway databases. We only considered the pathways that included the genes available in our RNA-seq dataset.

The raw RNA-seq count dataset contained NA values and underexpressed genes, which needed to be preprocessed prior to being used for the model. Genes with NA values in more than 70% of the samples were filtered out, resulting in 11,175 genes.

The next stage was to address dropout events in which a gene expressed even at a relatively high level may be undetected because of some technical limitations, including reverse transcription inefficiency.[114] We imputed the dropouts or underexpressed genes using scImpute[115] in R, a statistical method to accurately and robustly impute the dropouts. In our case, we detected gene expression values impacted by dropout events, and performed imputation only on those imputed values without impacting or introducing any bias to the remaining data. Using ScImpute, we also detected the outliers and removed them during the imputation process.[115]

CT scan images from 144 patients (out of the initial 211 patients) had labelled tumor segments, which could be used as ground truth for the U-Net model.[116] Further, each sample had a varying number of CT scan slices, but only a few of them were tumorous slices. The number of slices for each sample varied depending on the region and position of the undertaken CT scan. The number of tumorous slices also differed for each sample, depending on the size and location of the tumor. Therefore, only the CT scan image slices having their respective labelled segments were considered. Thus, we considered only 2358 tumorous slices and segment labels from 144 samples for the next step.

We then designed and trained a model based on the U-Net architecture[116] to segment the tumor from CT scan images. We paired the 2358 CT scan images and their corresponding binary mask segments (labels). Specifically, the pre-trained VGG-16 model[117] was used to construct the contraction path (encoder), while the expansion path (decoder) of the U-Net architecture was constructed using transposed convolutional layers. The model was trained using tumorous CT scan images and their respective labelled segments. The trained model was then used to predict the segment of unlabelled CT scan images so that those images could be used along with RNA-seq data. During the segmentation process, we used the Dice loss[53] as a loss function, and the intersection over union (Jaccard index) as an evaluation metric to measure the contact or overlap ratio between the predicted segment (PS) and ground truth (GT). Equation 1 reports the Dice coefficient formula:

$$DiceCoeff = \frac{2|PS \cap GT|}{|PS| \cup |GT|}, \quad \text{(Equation 1)}$$

where PS represents the predicted segment and GT represents the ground truth. $|PS \cap GT|$ represents the common elements between predicted segments and the ground truth. For binary image segmentation, GT is considered to be a set of foreground labelled pixels. The Dice coefficient can range from 0 (the PS does not overlap with the GT) to 1 (perfect agreement/overlap between the PS and GT). As a loss function to be minimized while training the model, the dice loss between two binary volumes 0 and 1 was computed as:

$$DiceLoss = 1 - \frac{2\sum_{i}^{N} P_i T_i + \epsilon}{\sum_{i}^{N} P_i^2 + \sum_{i}^{N} T_i^2 + \epsilon}, \quad \text{(Equation 2)}$$

where P_i is the predicted pixel value, T_i is the true pixel value, N is the total number of pixels in the image and ϵ is a small smoothing constant and was set to 1. In our experiment, $T_i \in \{0, 1\}$ and $0 < P_i < 1$.

After segmenting the tumor using the U-Net-based model, we used the OpenCV library to crop the tumorous region from CT scan images and resize the images to 224 x 224 pixels. Figure S1 illustrates the tumor segmentation process and the extraction of the Region of Interest (RoI). Then the segmented images, clinical data and RNA-seq data from 130 common patients were selected to train and validate the proposed survival prediction models.

Data for external validation

To evaluate the robustness of the trained models and their performance on unseen external datasets, two additional cohort datasets were collected from the TCGA repository: TCGA-LUAD and TCGA-LUSC.[48,49] The TCGA-LUAD dataset contains 28 joint samples of CT scan images, gene expression and clinical data. Among these samples, 19 are from female patients with an average age of 67.2 years, and 9 are from male patients with an average age of 69.22 years. Similarly, the TCGA-LUSC dataset contains 34 joint samples, comprised of 16 female patients with an average age of 71.56 years and 18 male patients with an average age of 64.11 years.

H-VAE-Cox: Hierarchical Variational Autoencoder-based Cox model

H-VAE-Cox is based on multimodal hierarchical integration approaches for combining multiple single-type models. Hierarchical and multimodal techniques are integration approaches that aim to compute higher-level classification or regression by integrating multiple modalities computed separately over distinct data types. H-VAE-Cox is a modular architecture in which a separate autoencoder was trained with each data type independently to generate low-dimensional features, minimising the information loss during the dimensionality reduction. This architecture is composed of two low-level autoencoders (see Low-level autoencoder: Extraction of pathway-guided latent features from RNA-seq data and Low-level autoencoder: Extraction of features from radiological (CT Scan) images) to extract the latent features from gene expression and images separately. The resulting latent features along with clinical data are then assembled in a high-level variational autoencoder (see High-level variational autoencoder: Cox model) to generate a vector of integrated latent features, which is then used to estimate the prognostic index (PI) for survival analysis.

Hence, H-VAE-Cox estimates the prognostic index from radiological images, gene expression, and clinical data in three steps (Figure 2). First, the high-dimensional gene expression was encoded to a pathway-guided lower-dimension latent vector using a sparse

autoencoder. Then, a supervised convolutional autoencoder encoded the multi-dimensional images to lower-dimension latent vectors. Finally, these latent features were concatenated to form an integrated input, which was fed into the high-level β-VAE along with the clinical data to estimate the prognostic index (PI), as discussed in the section High-level variational autoencoder: Cox model.

Low-level autoencoder: Extraction of pathway-guided latent features from RNA-seq data

The preprocessed RNA-seq data was a high-dimensional and low-sample size gene expression data, which constitutes one of the main challenges when working with omics data in the context of multimodal machine learning.[118] To overcome the curse of dimensionality and focus on biologically relevant genes based on pathway knowledge, we designed a supervised autoencoder that reduces the dimension of gene expression data in a way that generates pathway-guided latent features (Figure 2, step 1). The encoder of the gene sparse autoencoder consists of (i) an input layer, (ii) a gene layer, (iii) a pathway layer, and (iv) a latent feature layer Z_1. The preprocessed gene expression data g_0, with N samples and m genes (features), was used as the encoder input. The second layer of the encoder is the pathway layer with q nodes, where each node represents the biological pathway associated with the input genes.

To add biological knowledge to the network and implement the sparse connection between the gene and pathway layer, a pathway mask based on KEGG and Reactome was introduced. This was encoded as a binary matrix vector A of dimension $m \times q$, where m is the number of genes and q is the number of pathways. Each element of the pathway matrix was set equal to 1 if the gene is associated with the corresponding pathway, and equal to 0 otherwise. The neurons in the gene layers were sparsely connected to the neurons in the pathway layer (see Sparse connection between the gene and pathway layers for more details on the pathway mask). Thus, the pathway layer incorporates biological knowledge, and the autoencoder can learn from these biologically interpretable features.

On the other side, the decoder was constructed with three layers, where the latent features were fed as input to reconstruct the original gene expression data. Similar to the encoder part, a pathway matrix was introduced into the pathway layer. A Cox regression component was connected to the autoencoder bottleneck (latent) layer to predict the prognostic index and generate the latent features associated with survival prediction while having the capability to be used to reconstruct the original data. As a result of the Cox regression approach, the latent representation was further regularised through the Cox negative log likelihood loss function:

$$C_{loss} = \sum_{i=1}^{N} \delta_i \left\{ X_i'\gamma - \log\left[\sum_{j \in R(t_i)} e^{(X_j'\gamma)} \right] \right\} - P_\lambda(\gamma), \quad \text{(Equation 3)}$$

where $P_\lambda(\gamma)$ is a network-constrained penalty function on the coefficients γ, N is the number of samples, t_i is the survival times and δ_i is the censoring indicator for each sample ($\delta_i = 1$ if the survival time is observed and $\delta_i = 0$ if the survival time is censored), $R(t_i)$ is the risk set at time t_i, namely the set of all patients who still survived prior to time t_i. The function is used to estimate the PI for each individual, which is the linear predictor $X_j'\gamma$, where X_j' represents the weights of the linear combination of neurons in the previous layer. The Mean Squared Error (MSE) was used as the reconstruction loss:

$$\mathcal{L}_g MSE = \frac{1}{N} \sum_{i=1}^{N} \left(\hat{g}_0 - g_0\right)^2, \quad \text{(Equation 4)}$$

where N represents the number of samples, g_0 represents the input gene expression value and $\hat{g}_0$ represents the reconstructed gene expression value. An $\mathcal{L}_2$ regularisation loss was added to the Cox regression component to regularise the model and avoid overfitting. $\mathcal{L}_2$ is proportional to the squared magnitude of the coefficients (w_i), and it was included as a penalty term added to the loss function:

$$\mathcal{L}_2 = \lambda \sum_{i=1}^{N} {w_i}^2, \quad \text{(Equation 5)}$$

where λ is the regularisation coefficient set during the hyperparameter tuning phase. As a result, the total loss we used for the model is given by Equation 6:

$$\mathcal{L}_{total} = C_{loss} + \mathcal{L}_g MSE + \mathcal{L}_2. \quad \text{(Equation 6)}$$

This low-level supervised gene sparse autoencoder was used for two purposes: (i) to generate lower-dimensional latent features from gene expression data, and (ii) to perform survival prediction with gene expression data only.

Low-level autoencoder: Extraction of features from radiological (CT scan) images

To encode the multi-dimensional images into a low-dimension representation Z_2, we designed and trained a supervised convolutional autoencoder (Figure 2, step 2). Supervised dimensionality reduction aims to reduce higher or multi-dimensional data to lower dimensions in order to make classification and regression algorithms more effective. Our goal was to obtain an autoencoder that reduced the dimension of tumourous CT scan images such that the latent features Z_2 could: (i) be related to survival prediction, and (ii) reconstruct the original data with minimal error. The encoder and decoder parts were constructed using convolutional layers. Similar to the gene sparse autoencoder (Figure 2, step 1), a Cox regression component was connected to the autoencoder latent layer to predict the prognostic index. As a result of the Cox regression, the latent representation was further regularised with the Cox negative log likelihood loss function, as shown in Equation 3. An $\mathcal{L}_2$ regularisation loss was added to the Cox regression component to regularise further the model and avoid overfitting.

Let $D_i = (T_i, t, e)$ be the image dataset, where T_i represents cropped tumourous images, t is the time, and e is the event indicator (i.e., censored or uncensored). Let E and D be the encoder and decoder of the supervised convolutional autoencoder. The encoder encodes tumourous images as:

$$Z_2, pred\_hz = E_{\theta^e}(T_i, t, e), \quad \text{(Equation 7)}$$

where θ^e is the encoder weight matrix and $pred\_hz$ is the predicted risk score. However, in this process, the images would gradually lose information. To avoid this information loss, the decoder D reconstructs the images from the latent representation Z_2. The reconstruction of the images by the decoder can be represented as:

$$\widehat{T}_i = D_{\theta^d}(Z_2), \quad \text{(Equation 8)}$$

where θ^d is the weight matrix of the decoder.

We used the mean squared error loss as image reconstruction loss:

$$\mathcal{L}_{MSE} = \frac{1}{N}\sum_{i=1}^{N}\left(\widehat{T}_i - T_i\right)^2, \quad \text{(Equation 9)}$$

where N represents the number of samples, T_i represents the cropped tumourous images and $\widehat{T}_i$ represents the reconstructed images. The reconstruction loss is computed by comparing the pixel intensities of the original and reconstructed images.

An $\mathcal{L}_2$ regularisation loss was used to further regularise the model. Thus, the total loss for the image autoencoder is:

$$\mathcal{L}_{total} = \mathcal{C}_{loss} + \mathcal{L}_{MSE} + \mathcal{L}_2. \quad \text{(Equation 10)}$$

As a result, with minimal loss of information from the images, this supervised convolutional autoencoder generates latent features associated with survival prediction. This low-level autoencoder was used for two purposes: (i) to generate lower-dimensional latent features from multi-dimensional CT scan images; and (ii) to perform survival prediction using images only.

High-level variational autoencoder: Cox model

To integrate the latent features generated from the low-level autoencoders (i.e., the gene sparse autoencoder and the image autoencoder) for survival prediction, we designed a high-level β-variational autoencoder (β-VAE), based on the Cox regression. A β-VAE, unlike standard autoencoders, encodes the input as a distribution over a latent space rather than as a single point.[119] The latent features z_1 and z_z from the gene sparse autoencoder and image autoencoder, each of d-dimensions with N samples, were merged as an input (of dimension $2d$) for the encoder. We note that, besides the important information extracted from radiogenomics data, clinical data also plays a vital role in precise survival analysis and treatment planning. Hence, a separate clinical layer was introduced to the autoencoder to capture the clinical effect for survival prediction. The architecture of our β-VAE is therefore composed of the three components as outlined below: encoder, decoder, and Cox regression component.

Encoder

The latent features encoded from the gene expression autoencoder (see Low-level autoencoder: Extraction of pathway-guided latent features from RNA-seq data) and the image autoencoder (see Low-level autoencoder: Extraction of features from radiological (CT Scan) images) were concatenated and further encoded to form integrated radiogenomics low-dimensional features. Each latent variable z_i was encoded by the encoder using a latent distribution $p_\theta(\mathrm{z})$. The final hidden layer of the encoder was connected to two output layers, which represent the mean (μ) and the standard deviation (σ) of the Gaussian distribution $\mathrm{N}(\mu, \sigma)$ of the latent variable z_i given the input sample x, which corresponds to the variational distribution $q_\varphi(\mathrm{z|x})$. To estimate the posterior latent distribution and solve the intractability of the real posterior $p_\theta(\mathrm{z|x})$, the encoder inserts a variational distribution $q_\varphi(\mathrm{z|x})$, where φ is the encoder set of learnable parameters.[46] To make the sampling process differentiable and suitable for backpropagation, the reparametrisation trick was applied in the bottleneck layer as shown in Equation 11:

$$\mathbf{z} = \boldsymbol{\mu} + \boldsymbol{\sigma\epsilon}, \quad \text{(Equation 11)}$$

where $\mathbf{z}$ represents the latent feature vector, μ and σ represent the mean and standard deviation of the Gaussian distribution, respectively, and ϵ is a random variable sampled from $N(0,1)$.

Decoder

The points sampled from a conditional distribution $p_\theta(\mathrm{x|z})$, where θ is the decoder's set of learnable parameters, are decoded by the decoder, which reconstructs the input x as x'. Here, the β-VAE estimates the loss or error using a loss function composed of two losses, namely the reconstruction loss and the regularisation loss. To regularise the latent space, the reconstruction loss computes the loss for the reconstruction of input x' compared to the original input x, while the regularisation loss quantifies the distance between the estimated posterior $q_\varphi(\mathrm{z|x})$ and true posterior $p_\theta(\mathrm{z|x})$. The regularisation loss in a conventional VAE is the Kullback-Leibler divergence (Equation 12). However, for a β-VAE, the regularisation loss is multiplied by β (the regularisation coefficient, where $\beta > 1$). β constrains the capacity of the latent information channel Z and puts implicit independence pressure on the learnt posterior due to

the isotropic nature of the Gaussian prior $p_\theta(\mathrm{z})$. In our implementation, the encoder and decoder were jointly optimised using the following loss function, which relies on the traditional evidence lower bound (ELBO) criterion:

$$\mathcal{L}_{vae} = \mathbb{E}_{\mathrm{z}\sim q_\varphi(\mathrm{z}|\mathrm{x})} \log p_\theta(\mathrm{x}|\mathrm{z}) - \beta(D_{\mathrm{KL}}(q_\varphi(\mathrm{z}|\mathrm{x})\|p_\theta(\mathrm{z}|\mathrm{x})) - \epsilon), \quad \text{(Equation 12)}$$

where $\mathbb{E}_{\mathrm{z}\sim q_\varphi(\mathrm{z}|\mathrm{x})} \log p_\theta(\mathrm{x}|\mathrm{z})$ corresponds to the ELBO contribution and D_{KL} is the Kullback-Leibler (KL) divergence between two probability distributions.

Cox regression component

For the survival prediction using the integrated latent features in H-VAE-Cox, the β-VAE was combined with a Cox regression component. Clinical features contain relevant information for survival analysis, thus we introduced a 2-layer fully connected neural network to extract the features from the clinical dataset. Then, to estimate the PI, the encoder output vector μ was concatenated with the clinical layer's output, and connected to the Cox regression component. The final output layer (i.e., PI layer) of the Cox regression component is a single-neuron, non-linear hazard function parameterised by the weights of a linear combination of neurons in the previous layer.[120,121]

As part of the Cox regression, the latent representation was further regularised with the Cox negative log likelihood loss function, as shown in Equation 3. With the regularisation of the Cox regression component, the model is encouraged to acquire latent representations that can not only properly reconstruct the input sample, but also predict the hazard ratio for survival analysis. An $\mathcal{L}_2$ regularisation loss was added to the Cox regression component to regularise the model and avoid overfitting. Thus, the total loss function for H-VAE-Cox was composed of reconstruction loss, regularisation loss and Cox loss as follows:

$$\mathcal{L}_{total} = \mathcal{L}_{vae} + \mathcal{C}_{loss} + \mathcal{L}_2, \quad \text{(Equation 13)}$$

where $\mathcal{L}_{vae}$ is the reconstruction loss and regularisation loss obtained from Equation 12, and $\mathcal{C}_{loss}$ is the Cox loss presented in Equation 3. $\mathcal{L}_2$ is the squared magnitude of the coefficients as a penalty term added to the loss function.

XAT-VAE-Cox: Cross-attention-based sparse Variational Autoencoder Cox model

The second architecture we designed to integrate radiological images, gene expression, and clinical data and estimate Cox proportional hazard ratio is the Cross-attention-based sparse Variational Autoencoder Cox model (XAT-VAE-Cox). As shown in Figure 3, high-level representations of multiple data sources, e.g., multi-dimensional cropped tumourous images (T_i) and high-dimensional gene expression data (g_0 with N samples and m genes), were transformed into a single latent representation by learning to reconstruct these multiple data sources starting from a common latent representation. XAT-VAE-Cox, like the high-level autoencoder architecture of H-VAE-Cox, is composed of an encoder, a decoder, and a Cox regression component. In XAT-VAE-Cox, however, rather than low-level latent features from low-level autoencoders, both images and gene expression data were directly introduced into the model as input and reconstructed by the β-VAE.

In the encoder phase, two different modalities, namely imaging and transcriptomics, were introduced to overcome the integration challenges between multi-dimensional images and high-dimensional gene expression data, incorporating biological information into the network. The imaging modality, constructed using a single-head self-attention layer and pre-trained VGG-19, in the encoder is responsible for extracting the features from tumourous images T_i. In particular, the imaging modality consists of a convolutional layer, a single-head self-attention layer and four layers from the pre-trained VGG-19 model. The multi-dimensional features from the images are then transformed into a low-dimensional feature vector (of dimension q).

Similarly, the transcriptomics-specific gene modality is the encoder's second input, where the first layer is used to introduce the gene expression data g_0. The pathway layer is the second layer in this modality, with q nodes representing the biological pathways associated with the m input genes. Before this layer, a pathway mask based on KEGG and Reactome databases was introduced to add biological knowledge to the model and implement a sparse connection between the gene and pathway layers (see Sparse connection between the gene and pathway layers). This is a binary matrix A of dimension $m \times q$, where m is the number of genes, and q is the number of pathways. $A(i,j) = 1$ if the i-th gene is related to the j-th pathway. The neurons in the gene layer were therefore sparsely connected to the neurons in the pathway layer. The node values in the pathway layer reflect the associated pathways as high-level representations for the survival model, which allows the autoencoder to learn biologically interpretable features. To generate a high-quality latent representation from the gene modality and highlight the features relevant to survival prediction, a multi-head self-attention mechanism was implemented within the gene modality. The pathway-guided low-dimensional features of size q from this modality were considered query, key and value for the multi-head self-attention layer (see 1 for details on the attention mechanism implemented).

Furthermore, to learn cross-modal interactions between images and gene expression, two layers of the multi-head cross-attention mechanism were implemented. In particular, the first cross-attention layer was constructed considering the latent representation of size q from the imaging modality as query, and the output of the self-attention layer of size q from the gene modality as key and value. Similarly, the second cross-attention layer was constructed considering the output of the self-attention layer of size q from the gene modality as query, and the latent representation of size q from imaging modality as key and value. Finally, the outputs of these two cross-attention layers, each of size q, were concatenated to form $N\times2q$ dimensional vectors.

This concatenated layer was then connected to two output layers. In the Gaussian distribution $\mathrm{N}(\mu,\sigma)$ of the latent feature z, given the input samples $\mathrm{T_i}$ and $\mathrm{g_0}$, these two layers represent the mean μ and the standard deviation σ. A reparameterisation

method was used in the bottleneck layer i.e., $z = \mu + \sigma\epsilon$, where ϵ is a random variable sampled from the unit normal distribution $N(\mathbf{0}, \mathbf{1})$ to make the sampling process differentiable and appropriate for backpropagation during the training phase. The sampled latent feature vector is the low-dimensional representation of the integrated features from the cropped tumourous images and gene expression data.

In the decoder phase, the points sampled from a conditional distribution $p_\theta((\mathrm{T_i}, \mathrm{g_0})|\mathrm{z})$ were decoded to reconstruct the input images T_i and gene expression g_0, where θ is the decoder's set of learnable parameters. The β-VAE uses a loss function composed of two losses, reconstruction loss and regularisation loss, to estimate the error. The reconstruction loss computes the loss for the image and gene expression reconstruction (T_i' and g_0', respectively). In particular, the sampled points were split into two branches that produce individual reconstructions of input images and gene expression. The final reconstruction loss was obtained by combining two different reconstruction loss functions for images and gene expression. The regularisation loss quantifies the distance between the estimated posterior $q_\varphi(\mathrm{z}|(\mathrm{T_i}, \mathrm{g_0}))$ and true posterior $p_\theta(\mathrm{z}|(\mathrm{T_i}, \mathrm{g_0}))$. The regularisation loss, Kullback-Leibler divergence (Equation 12), was multiplied by β, the regularisation coefficient $(\beta > 1)$. As for H-VAE-Cox, the loss function of the β-VAE is composed of three losses: image reconstruction loss, gene reconstruction loss and β-regularisation loss (D_{KL}), as illustrated in Equation 14:

$$\mathcal{L}_{x-vae} = \mathcal{L}_T MSE + \mathcal{L}_g MSE - \beta(D_{\mathrm{KL}}(q_\varphi(z|(T_i, g_0))\|p_\theta(z)) - \epsilon), \quad \text{(Equation 14)}$$

where $\mathcal{L}_T MSE$ is the image reconstructed loss, $\mathcal{L}_g MSE$ is the gene expression reconstruction loss, D_{KL} is the Kullback-Leibler (KL) divergence between two probability distributions, and β is the regularisation coefficient.

The latent vector μ from the β-VAE was then integrated into a Cox regression component. As done for H-VAE-Cox, the encoder's output latent vector μ was concatenated with the clinical layer's output and then linked to the subsequent Cox regression component. As a result of the Cox regression, the latent representation was further regularised with a Cox negative log likelihood loss function (Equation 3). Therefore, the total loss function for XAT-VAE-Cox was composed of image reconstruction loss, gene expression reconstruction loss, regularisation loss, and Cox loss, as illustrated in Equation 15:

$$\mathcal{L}_{total} = \mathcal{L}_{x-vae} * \mathcal{K}_{vl} + (\mathcal{C}_{loss} + \mathcal{L}_2) * \mathcal{K}_{cl}, \quad \text{(Equation 15)}$$

where $\mathcal{L}_{x-vae}$ is the reconstruction and regularisation loss in Equation 14, and $\mathcal{C}_{loss}$ is the Cox negative log likelihood loss in Equation 3. $\mathcal{L}_2$ is the squared magnitude of the coefficients used as penalty term added to the loss function. $\mathcal{K}_{vl}$ and $\mathcal{K}_{cl}$ are the regularisation weights of the autoencoder loss and Cox loss, respectively.

Sparse connection between the gene and pathway layers

Let us consider the gene expression input feature vector $g_0 \in R^m$, the gene layer G, and the pathway layer P with ReLU activation function $\sigma_{\mathrm{R}}(x) = \max(0, x)$. Let the number of neurons in the gene and pathway layers be m and q, respectively. Initially, the two layers were fully connected, where the number of connections is quadratic in the number of neurons. The forward pass for fully connected layers can be represented by a matrix as:

$$f_{g_0} = \sigma_{\mathrm{R}}\left(\mathrm{W}^T . \sigma_{\mathrm{R}}\left(\mathrm{W_0}^T . g_0 + \mathrm{b_0}\right) + \mathrm{b}\right), \quad \text{(Equation 16)}$$

where $\mathrm{W_0}$ is the weight matrix of dimensions $\mathrm{m} \times \mathrm{m}$ for the gene layer, $\mathrm{b_0} \in \mathbb{R}^m$ is the bias vector for the gene layer, W is the weight matrix of dimensions $\mathrm{m} \times \mathrm{q}$ for the pathway layer, and $\mathrm{b} \in \mathbb{R}^q$ is the bias vector for the pathway layer. Hence, the network function f_{g_0} is parameterised by weight matrices $\mathrm{W_0}$, W and biases b0, b. The weights and biases are randomly initialised and are then optimised during the training phase using the backpropagation on fully connected layers.

In our architecture, in order to force sparse connections between the gene layer and the pathway layer, the weight of the pathway layer (W) was multiplied by a binary matrix $A \in \mathbb{R}^{m \times q}$ to incorporate the information of membership gene-pathway taken from KEGG and Reactome databases. The sparse connection was created during the training process by updating the weight of neurons in the pathway layer after each training epoch. The output of the sparse network function was therefore computed as:

$$f_{g_0} = \sigma_{\mathrm{R}}\left((\mathrm{W} \bigstar \mathrm{A})^T . \sigma_{\mathrm{R}}\left(\mathrm{W_0}^T . g_0 + \mathrm{b_0}\right) + \mathrm{b}\right), \quad \text{(Equation 17)}$$

where ★ represents the element-wise matrix multiplication.

Attention mechanism

The attention mechanism is implemented in the encoder of the XAT-VAE-Cox model, which enables the model to focus attention on input features during output generation.[122] In particular, the self-attention implemented in the imaging and gene modalities enables intra-modality communication and focuses on important input features from each modality relevant to the output. The self-attention matrix is calculated as:

$$\mathrm{Attention}(Q, K, V) = \mathrm{softmax}\left(\frac{QK^T}{\sqrt{d_k}}\right)V, \quad \text{(Equation 18)}$$

where Attention(Q, K, V) defines the function that computes a weighted sum of the value vectors V, where the weights are determined by the similarity between the query vector Q and the key vectors K. The similarity is measured by the dot product of Q and K, scaled by the inverse square root of the key dimension d_k. The softmax function normalises the dot products into a probability distribution. For modality-specific self-attention, the query, key, and value vectors are all derived from the same input modality, i.e., image and gene modality.

The latent representations from the image and gene modalities are connected within the encoder via a multi-head cross-attention mechanism. In particular, the multi-head cross-attention mechanism is a technique that allows the model to learn from two different modalities, imaging modality and gene modality, by attending to both of them simultaneously. It is similar to self-attention, but instead of using the single modality for query, key, and value, it uses one modality as query and another modality as key and value. This enables the encoder to capture the cross-modal relations and to align the features from both modalities.

In order to establish two-way communication between two modalities, two multi-head cross-attention layers were constructed. First, the cross-attention layer was constructed considering the latent representation from the imaging modality as query Q of dimension q, and the latent representation from the gene modality as key K and value V of dimension q. Then the second multi-head cross-attention layer was constructed considering the latent representation from the gene modality as query Q and the latent representation from the imaging modality as key K and value V. These two cross-attention layers were then concatenated to form a single latent representation layer for the encoder (Figure 3).

The multi-head cross-attention layer is mathematically represented as:

$$\text{MultiHead}(Q, K, V) = \text{Concat}(\text{head}_1, \ldots, \text{head}_h)W^O, \quad \text{(Equation 19)}$$

where

$$\text{head}_i = \text{Attention}\left(QW_i^Q, KW_i^K, VW_i^V\right). \quad \text{(Equation 20)}$$

Here, Q, K, and V are the query, key, and value matrices from different modalities, W_i^Q, W_i^K, W_i^V, and W^O are learnable weight matrices, h is the number of heads which is selected via hyperparameter tuning, and Concat is the concatenation operation.

Experimental design and model evaluation

The first step of our analysis was to identify and segment the tumor and extract the region of interest from CT scan images. We trained the U-Net-based model (see Data collection and preprocessing for details of the U-Net model) for 100 epochs using the Dice coefficient as a loss function (Equation 1). We adopted a K-fold validation approach to train, test and evaluate the model using 2358 labelled CT images from 144 samples. The pre-trained model was then used to segment the tumor from unlabelled/unsegmented samples. To avoid the disturbance of other organs, the tumor region was cropped to 224 x 224 pixels using the OpenCV python library, with the tumor centered in each image. These cropped tumor regions were used in the H-VAE-Cox and XAT-VAE-Cox models, along with gene expression and clinical data for the same samples.

To determine the effect of including each data type, the predictive performance of survival outcomes of the H-VAE-Cox and XAT-VAE-Cox models were evaluated and compared in five input scenarios: (i) the cropped tumor regions from CT scan images, gene expression, biological pathway, and clinical data; (ii) CT scan images only; (iii) CT scan images along with clinical data; (iv) gene expression data only; and (v) gene expression with clinical data. We used the concordance index (C-index)[52] to assess the predictive performance of the models including censored data. The C-index is a rank correlation metric that counts concordant pairs between the predicted scores and the observed survival times. The C-index ranges between 0 and 1, with 1 indicating an ideal prediction, and 0.5 indicating a random prediction.

To achieve high accuracy and avoid overfitting, deep learning architectures normally require large datasets. However, one of the challenges in our case was the modest size of the NSCLC dataset, with only 130 samples containing all three data types (images, gene expression, and clinical data). In principle, to eliminate bias in the model, the test dataset should be used only once, after separation from the training set (holdout validation). However, using holdout validation with a small dataset often leads to overfitting and makes the model pessimistically biased.[123] To overcome this limitation and make the model robust, we adopted a nested cross-validation approach to randomly split the dataset into smaller folds (see Figure 1F). Nested cross-validation is a technique that involves training a model and tuning the hyperparameters on a subset of data, and then validating the trained model with the best hyperparameters on the remaining data. The process is repeated multiple times (on different folds) and the average of the validation errors is computed to estimate the model generalisation performance. Since the test data is never used during each training process, the entire dataset can be used to estimate the PI, hence reducing the bias. The nested cross-validation has two loops (outer loop and inner loop), where the inner loop is used for hyperparameter tuning and training the model with the best hyperparameter, while the outer loop is used for validation and survival prediction.

To assess the reproducibility and performance of the proposed models, we repeated the experiment five times on different combinations of the omics data: (a) the cropped tumor regions from CT scan images, gene expression, biological pathway, and clinical data; (b) images data only; (c) images along with clinical data; (d) gene expression data only; (e) gene expression with clinical data; (f) clinical data only. For each experiment, the outer loop was split into 5-folds with stratification based on the survival status, ensuring the same percentage of censored and uncensored data in each fold, while the inner loop was used for hyperparameter tuning and

training the model with the best hyperparameters (see Section 'model tuning and hyperparameter optimisation'). The evaluation of the trained model with the best hyperparameter was performed on the outer fold.

Finally, in order to evaluate the robustness of the proposed models, for each outer loop, the performance was evaluated on two additional datasets from external cohorts, TCGA-LUAD and TCGA-LUSC. For each outer fold, we estimated the PI, and evaluated the performance of all models using the C-index, the C-index IPCW, and the cumulative dynamic AUC, and identified high- and low-risk patients (see Survival prediction with H-VAE-Cox and XAT-VAE-Cox outperforms other models).

Model interpretation

To interpret the models and identify important features contributing towards high-risk patients, we used the SHAP library to evaluate Shapley values (see Results section). As we adopted the nested cross-validation approach to train and validate the model, the SHAP values for images, gene expression, and clinical data were computed for high-risk samples identified from each outer loop validation dataset.

We then investigated the contribution of each modality towards the predictive performance of both models. Hence, the multimodality score for each modality was computed based on SHAP values to quantify the proportions of the contribution of each modality.[124] Equation 21 defines the imaging contribution Φ_I, gene expression contribution Φ_G, and clinical contribution Φ_C towards the prediction, where the SHAP value for each data modality is expressed as an absolute sum. The magnitude of the SHAP values was studied as we were interested in quantifying whether the features from each modality are actively contributing towards PI estimation, irrespective of the direction of contribution.

$$\Phi_I = \sum_j^{N_I} |\varphi_j|; \quad \Phi_G = \sum_j^{N_G} |\varphi_j|; \quad \Phi_C = \sum_j^{N_C} |\varphi_j|, \qquad \text{(Equation 21)}$$

where Φ_I, Φ_G and Φ_{CL} are the image, gene expression and clinical contributions towards the prediction, expressed as the absolute sum of SHAP values for each data modality (image, gene expression and clinical data). N_I, N_G and N_C represent the number of imaging, gene and clinical features.

To assess the extent to which each modality was contributing to the final prediction, we then calculated the multimodality scores. The multimodality score for each data modality is estimated as the proportion of the contribution of each data modality to the total contribution:

$$\begin{aligned} IM - score &= \frac{\Phi_I}{\Phi_I + \Phi_G + \Phi_C}, \\ GE - score &= \frac{\Phi_G}{\Phi_I + \Phi_G + \Phi_C}, \\ CL - score &= \frac{\Phi_C}{\Phi_I + \Phi_G + \Phi_C}, \end{aligned} \qquad \text{(Equation 22)}$$

where $IM - score$, $GE - score$ and $CL - score$ represent the multimodality score for image, gene expression and clinical features, expressed as a proportion of data modality contribution.

To further interpret the imaging modality of both models, we used Grad-CAM to visualise a heatmap concentrating on the significant regions contributing to the estimation of PI. To create the heatmap that visualises the important regions of the input image, Grad-CAM uses the output-specific gradient information, and feeds it into the self-attention layer for the XAT-VAE-Cox model and the final convolutional layer for the H-VAE-Cox model. The mathematical description of Grad-CAM is as follows.

Given an image I, let $f^k(I)$ be the activation map for the last CNN layer k for the H-VAE-Cox model and self-attention layer k for XAT-VAE-Cox model, and Y be the PI from the output layer. The gradient of Y with respect to f^k, denoted as $\frac{\partial Y}{\partial f^k}$, captures the importance of the activations for the estimated PI. These gradients are global-average-pooled to obtain the neuron importance weights α_k:

$$\alpha_k = \frac{1}{Z}\sum_i\sum_j \frac{\partial Y}{\partial f_{ij}^k}, \qquad \text{(Equation 23)}$$

where Z is the number of pixels in the activation map, and i,j index these pixels. The Grad-CAM heatmap $L_{\text{Grad}-\text{CAM}}$ is then a weighted combination of forward activation maps, followed by a ReLU function to only consider features with a positive influence on the estimation of PI:

$$L_{\text{Grad}-\text{CAM}} = \text{ReLU}\left(\sum_k \alpha_k f^k\right). \qquad \text{(Equation 24)}$$

This heatmap was then overlaid on the original image to show the discriminative regions used by the CNN to estimate the PI. The resulting heatmaps therefore highlight the important regions contributing to the PI estimation in both models.

Model tuning and hyperparameter optimisation

To tune the hyperparameters and avoid overfitting, we used a robust hyperparameter optimisation approach. The hyperparameters identified for our models are the learning rate, dropout rate, $\mathcal{L}_2$ regularisation, $\mathcal{K}_{vl}$, $\mathcal{K}_{cl}$, and the number of neurons in the Cox regression component. To train the model, a scheduled learning rate was chosen, with the learning rate decaying exponentially to meet the global minimum. The Keras tuner's Bayesian Optimisation was used to select the best hyperparameters for H-VAE-Cox and XAT-VAE-Cox. Each generated model was trained for 20 epochs during the optimisation or tuning process, with a maximum of three trials and two executions per trial. Although, in general, 20 epochs are insufficient to generate a well-trained model, they were sufficient in our case to generate a model for comparison. At the end of the optimisation, the model was built with the optimal hyperparameters and was then trained for 100 epochs to obtain the most optimised model.

We used the ReLU activation function for the encoder and decoder networks in both the proposed models as it ensured the lowest reconstruction loss, and we used the tanh activation function in the Cox regression component and linear activation function in the Cox output layer, as tanh produced the highest C-index compared to the ReLU activation function. The entire hyperparameter tuning process was performed within a nested cross-validation approach, where the inner loop was used for hyperparameter tuning while the outer loop validation data was used for validating the tuned model. The hyperparameters Capacity max iter, gamma, max capacity, and kld weight, responsible for determining the value of tanh in a β-VAE for both H-VAE-Cox and XAT-VAE-Cox models were set to 1e5, 1000, 25, and 0.005, respectively, based on previous experiments.[119]

QUANTIFICATION AND STATISTICAL ANALYSIS

To assess the statistical significance of the C-index for various input combinations, paired and pairwise t-tests were calculated for: (a) images only, (b) image and clinical data, (c) gene expression data only, (d) gene expression and clinical data (e) integrated features of images, gene expression and clinical data for H-VAE-Cox, (f) integrated features of images, gene expression and clinical data for XAT-VAE-Cox, (g) integrated features of images, gene expression and clinical data for DCM, and (h) integrated features of images, gene expression and clinical data for DeepSurv (Figure 4H).

At a 5% significance level, the adjusted p-value with Bonferroni correction was used to analyze the statistical difference. All the models were trained five times, using nested cross-validation with five outer loops, and the statistical significance of the C-index was compared. The integration of imaging, gene expression and clinical data using H-VAE-Cox and XAT-VAE-Cox demonstrated significant improvement in the survival prediction with p-value <0.05. The average C-index by the H-VAE-Cox and XAT-VAE-Cox models for three data modalities integration is higher than single and two data modalities, indicating that the integration of imaging, gene expression and clinical data significantly improves the accuracy of the survival prediction.

Cell Reports Methods, Volume *4*

Supplemental information

Cross-attention enables deep learning on limited omics-imaging-clinical data of 130 lung cancer patients

Suraj Verma, Giuseppe Magazzù, Noushin Eftekhari, Thai Lou, Alex Gilhespy, Annalisa Occhipinti, and Claudio Angione

Cross-attention enables deep learning on limited omics-imaging-clinical data of 130 lung cancer patients

Suraj Verma[1], Giuseppe Magazzù[2], Noushin Eftekhari[3], Thai Lou[4], Alex Gilhespy[5], Annalisa Occhipinti[1,6,7], and Claudio Angione *[1,6,7]

[1]School of Computing, Engineering & Digital Technologies, Teesside University, Tees Valley TS1 3BA, UK
[2]York St John University, York YO31 7EX, UK
[3]The Alan Turing Institute, London NW1 2DB, UK
[4]Gateshead Health NHS Foundation Trust, Gateshead NE9 6SX, UK
[5]South Tyneside and Sunderland NHS Foundation Trust, Sunderland SR4 7TP, UK
[6]Centre for Digital Innovation, Teesside University, Campus Heart, Tees Valley TS1 3BX, UK
[7]National Horizons Centre, Darlington DL1 1HG, UK

June 16, 2024

*Corresponding author

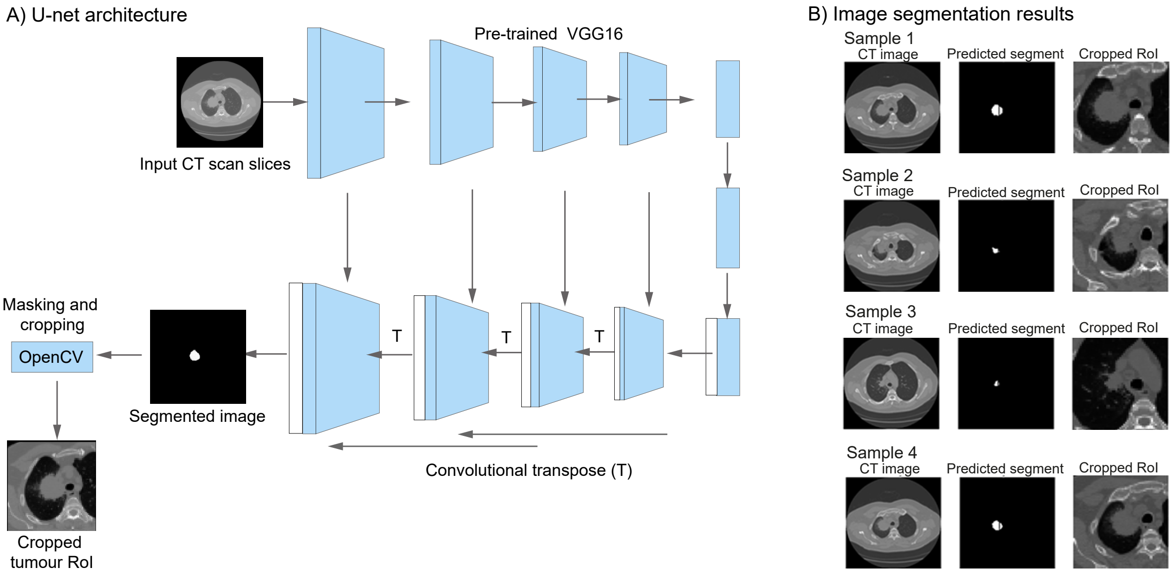

Figure S1: **Preprocessing of CT-scan images to extract regions of interest, related to STAR Method.** (A) A U-net-based architecture was designed using a pre-trained VGG16 model in the contraction path. Transpose convolutional layers were implemented in the expansion path. Using the OpenCV library, the predicted segment was masked with a tumour image and the tumour region was cropped to extract the Region of Interest (RoI). (B) Examples of tumour regions segmented using the U-Net model and RoI extracted using the OpenCV library after the segmentation pipeline.

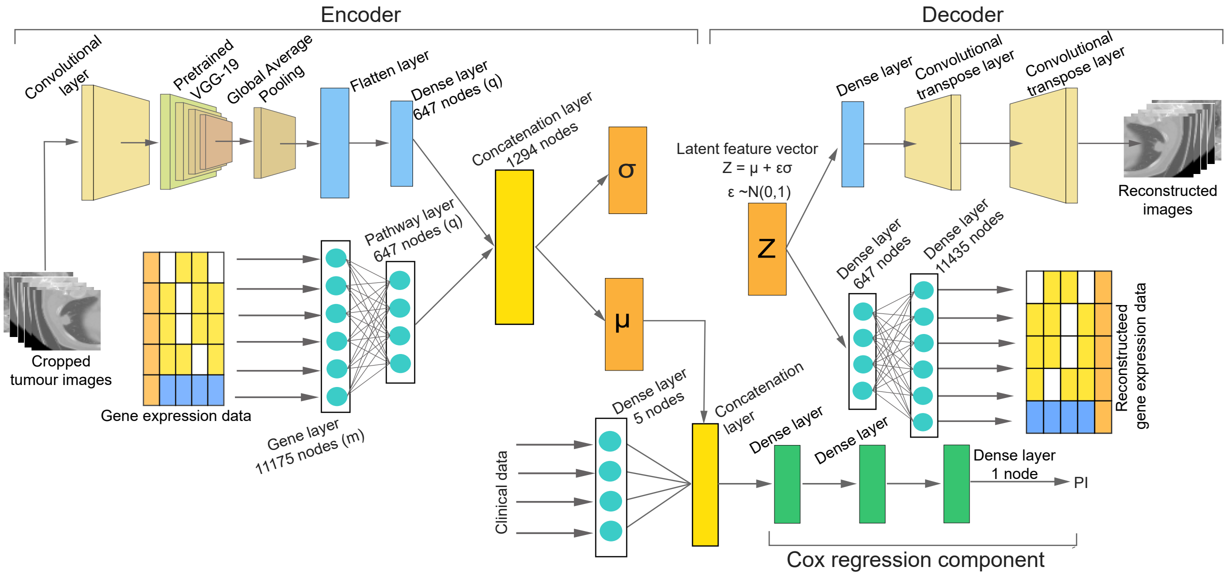

Figure S2: **Baseline-VAE-Cox: Variational Autoencoder-based Cox model, related to Figure 3.** The β-Variational Autoencoder architecture consists of encoder and decoder phases made from a convolutional layer, pre-trained VGG-19 layers, and dense layers for image and gene expression data. The latent vector μ is linked to the Cox regression component, which concatenates the latent vector and clinical features to estimate the Prognostic Index (PI).

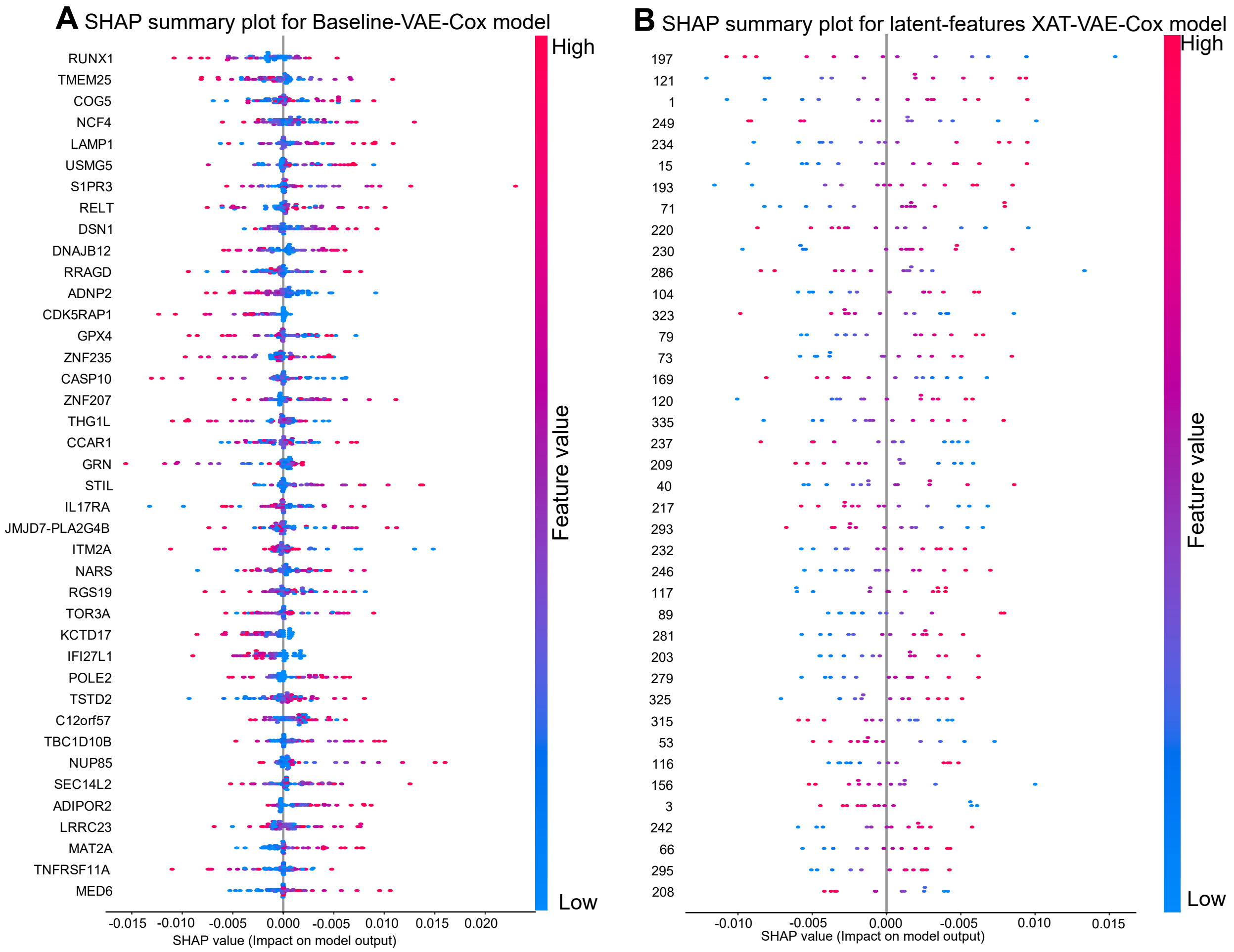

Figure S3: **Feature importance by SHAP summary plot, related to Figure 5** (A) Summary plot for top 40 important genes for Baseline-VAE-Cox model. (B) Summary plot for top 40 latent features μ generated by the integration of imaging and gene expression in the XAT-VAE-Cox model. As the XAT-VAE-Cox model generates a single latent representation from both image and gene modalities, the interpretation of these latent features did not provide any insights into the contribution of each modality towards the estimation of the prognostic index.

Figure S4: **Grad-CAM-highlighted CT scan images validated by the radiologists, related to Figure 5.** The following four figures report the labelling performed by two radiologists, who validated the tumour areas highlighted by the Grad-CAM heatmaps. Each image was evaluated based on three questions. The first question was whether the heatmap matched the tumour region, with a yes or no answer. The second and third questions asked for a rating from 1 to 4 on the quality and clarity of the heatmaps (see Main Manuscript).

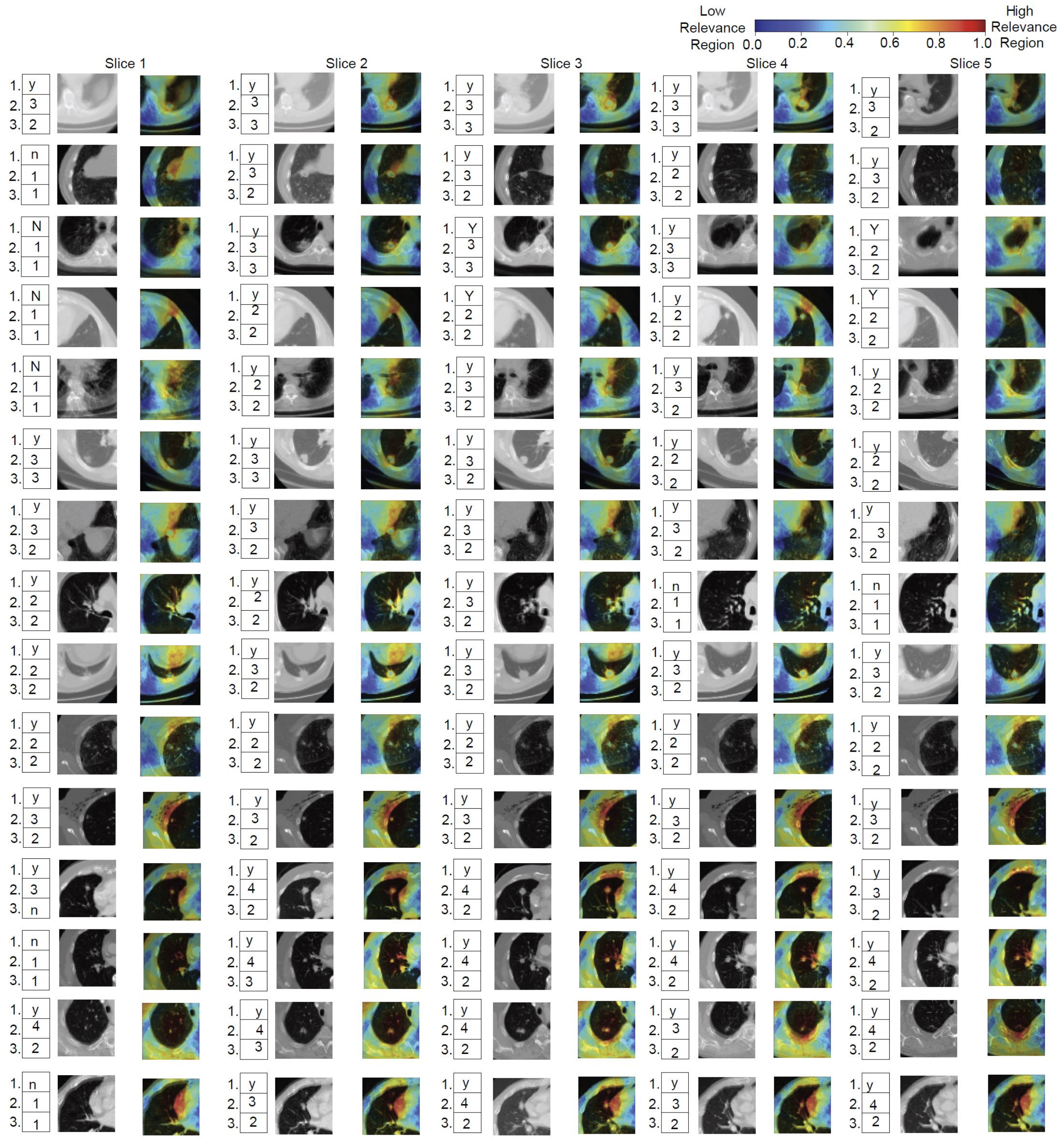

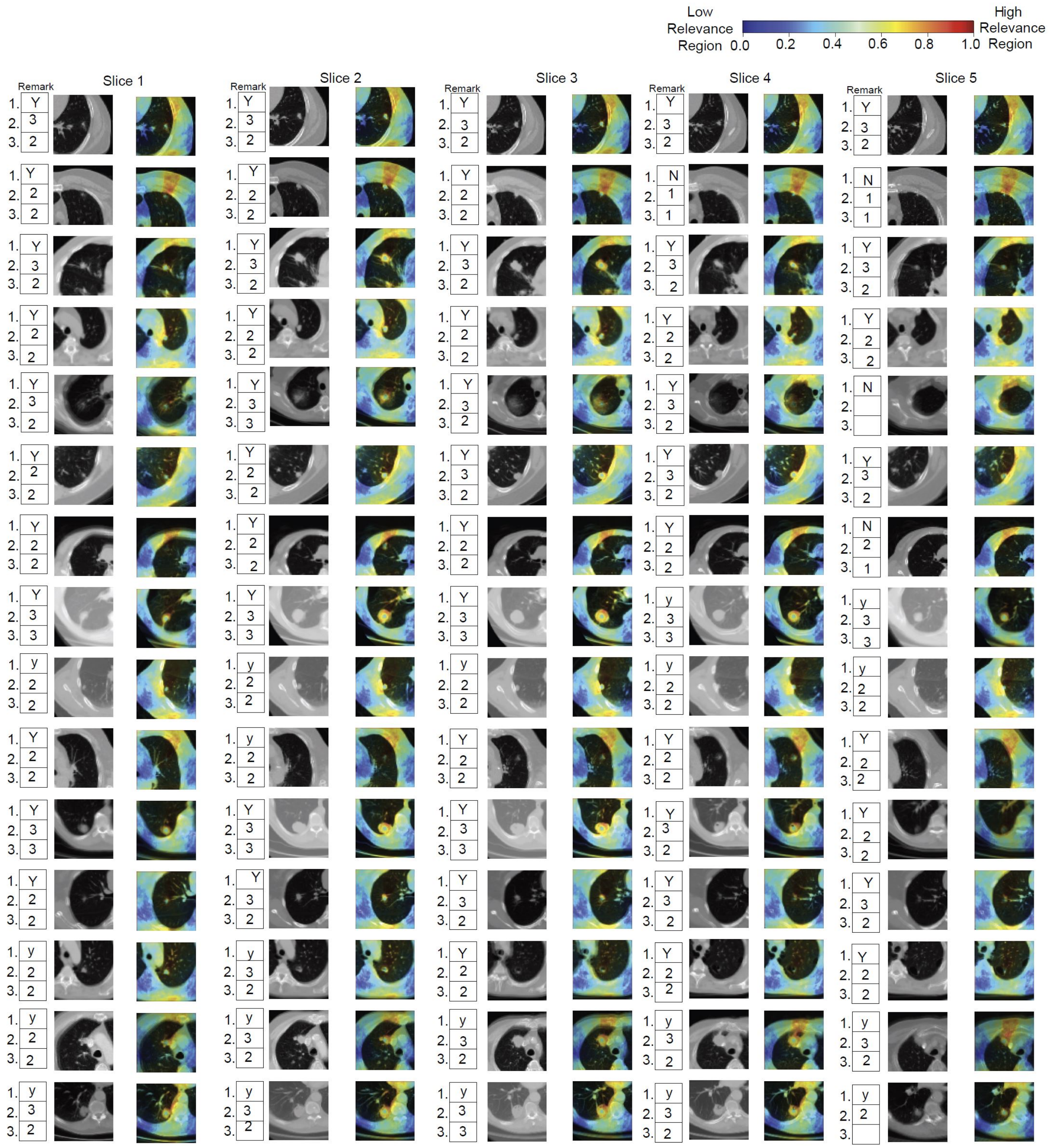
Low Relevance Region
0.0
0.2
0.4
0.6
0.8
1.0
High Relevance Region
Remark
Slice 1
Slice 2
Slice 3
Slice 4
Slice 5

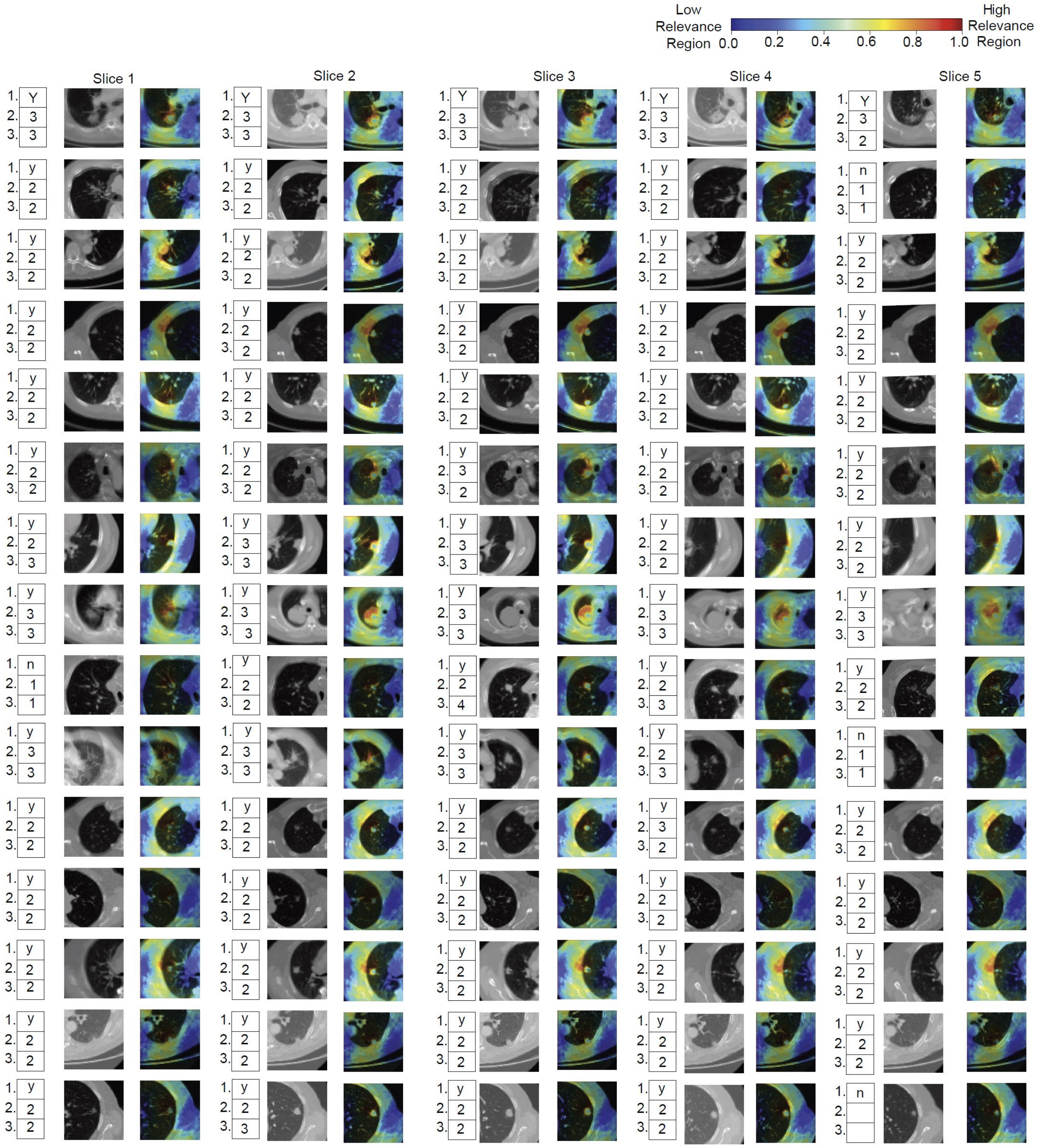
Low Relevance Region
0.0
0.2
0.4
0.6
0.8
1.0
High Relevance Region
Slice 1
Slice 2
Slice 3
Slice 4
Slice 5

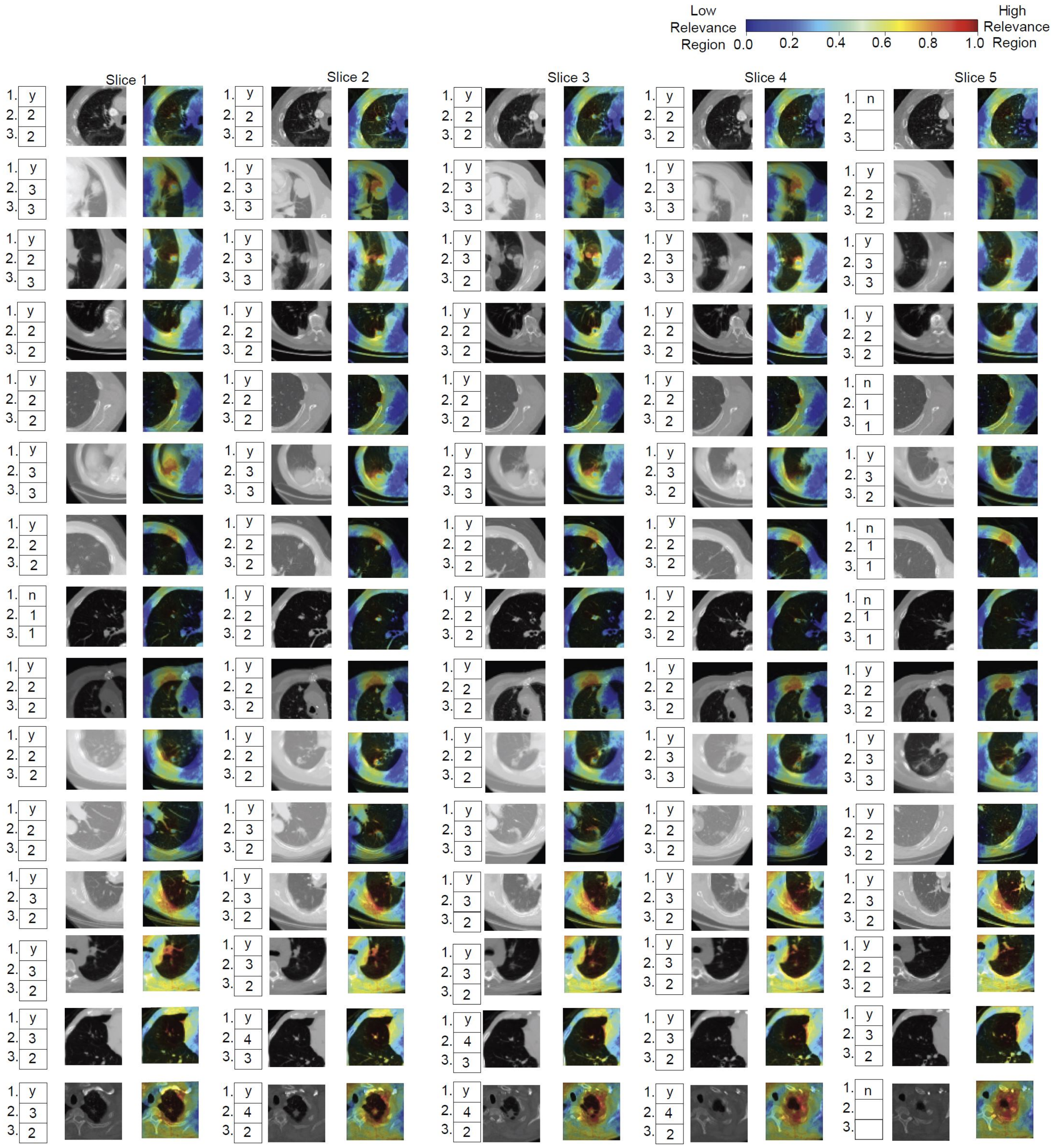
Low Relevance Region
0.0
0.2
0.4
0.6
0.8
1.0
High Relevance Region
Slice 1
Slice 2
Slice 3
Slice 4
Slice 5

Supplementary Tables

Models	Dataset	C-index IPCW (mean ± s.d)	Cumulative dynamic AUC (mean ± s.d)
H-VAE Cox	NSCLC-Radiogenomics	0.74 ± 0.09	0.73 ± 0.09
H-VAE Cox	TCGA-LUAD	0.71 ± 0.08	0.69 ± 0.09
H-VAE Cox	TCGA-LUSC	0.67 ± 0.10	0.68 ± 0.14
XAT-VAE-Cox	NSCLC-Radiogenomics	**0.77 ± 0.06**	**0.76 ± 0.06**
XAT-VAE-Cox	TCGA-LUAD	0.68 ± 0.03	0.69 ± 0.04
XAT-VAE-Cox	TCGA-LUSC	0.68 ± 0.05	0.71 ± 0.05
DeepSurv	NSCLC-Radiogenomics	0.60 ± 0.11	0.59 ± 0.13
DeepSurv	TCGA-LUAD	0.52 ± 0.31	0.49 ± 0.21
DeepSurv	TCGA-LUSC	0.53 ± 0.12	0.52 ± 0.02
DCM	NSCLC-Radiogenomics	0.64 ± 0.12	0.71 ± 0.14
DCM	TCGA-LUAD	0.57 ± 0.14	0.55 ± 0.11
DCM	TCGA-LUSC	0.52 ± 0.16	0.61 ± 0.13

Table S1: **Performance results of the proposed models when evaluated on NSCLC-Radiogenomics, TCGA-LUAD and TCGA-LUSC datasets in terms of C-index IPCW and Cumulative dynamic AUC (mean and standard deviation), related to Figure 4.** For each model, the nested cross-validation was repeated five times and the overall performance was presented in terms of mean and standard deviation. The values in bold show the best performance for each metric. Overall, the optimal performance was obtained by the XAT-VAE-Cox model, with a C-index IPCW of 77% and cumulative dynamic AUC of 76%.

Dataset	C-index (mean ± s.d)	C-index IPCW (mean ± s.d)	Cumulative dynamic AUC (mean ± s.d)
NSCLC-Radiogenomics	0.59 ± 0.08	0.63 ± 0.07	0.61 ± 0.11
TCGA-LUAD	0.58 ± 0.02	0.60 ± 0.02	0.60 ± 0.01
TCGA-LUSC	0.52 ± 0.05	0.58 ± 0.04	0.61 ± 0.05

Table S2: **Performance evaluation of the baseline-VAE-Cox model in terms of C-index, C-index IPCW and Cumulative dynamic AUC, related to Figure 4**
